# Functionalisation of homopropargyl boronic esters *via* hydrozirconation followed by Pd-catalysed cross coupling reaction

**DOI:** 10.1039/d6ra02170k

**Published:** 2026-03-24

**Authors:** Patrick Schäfer, Uli Kazmaier

**Affiliations:** a Organic Chemistry, Saarland University P. O. Box 151150 66041 Saarbrücken Germany u.kazmaier@mx.uni-saarland.de

## Abstract

Hydrozirconation of homopropargyl boronic esters accessible *via* Matteson homologation allows their selective functionalisation while retaining the boronic ester functionality. In this study, reactions of vinyl zirconium reagents derived from boronic esters in Negishi coupling, in particular with benzyl halides and aryl iodides, are presented. Different synthesis routes for the stereoselective assembly of (*E*)- and (*Z*)-alkenes are discussed.

## Abstract 2

Hydrozirconation of homopropargyl boronic esters accessible *via* Matteson homologation allows their selective functionalisation while retaining the boronic ester functionality.

## Graphical abstract

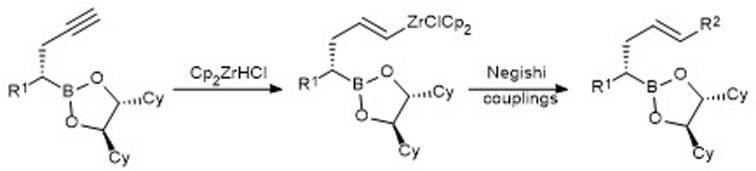

## Introduction

**Scheme 1:**
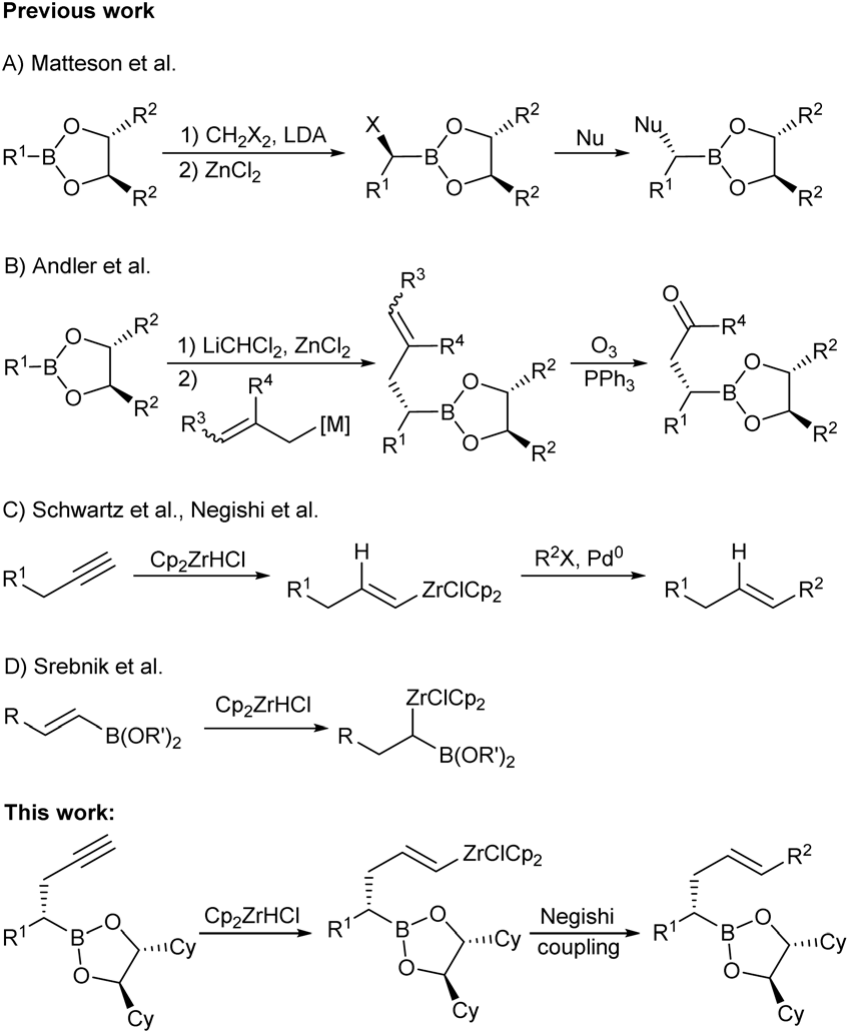
Previous and new work on Matteson homologation and hydrozirconation.

Polyketides form a wide-spread and diverse class of natural products with various biological activities.^1^ Therefore, the asymmetrical overall synthesis of the members of this class is of great importance, and it is often anything but trivial.^2^ In most cases, the polyketide chain is synthesized *via* aldol reactions^3^ or allylations/crotylations followed by ozonolysis.^4^ While these methods are suitable for multiple hydroxylated polyketide chains, specially substituted alkyl chains can often be better obtained *via* the Matteson homologation approach.^5^ This protocol was first described in the 1980s by Donald Matteson *et al.* in its asymmetrical version.^6^ Using alkylboronic esters of chiral diols, a carbon chain can be highly stereoselectively extended by reacting with a halogenated lithium carbenoid.^7^ The resulting α-haloboronic ester can then be subjected to a variety of *C*-, *O*- and *N*-nucleophiles such as Grignard^6*b*,8^ or organolithium reagents,^9^ alkoxides^10^ and azides (Scheme 1A).^11^ This allows each carbon atom of the chain to be substituted individually without getting into matched/mismatched situations, as the configuration of newly formed stereocentres is almost exclusively controlled by the chiral boronic ester. Functional groups that are not compatible with the homologation conditions, *e.g.* carbonyl groups, can be inserted *via* placeholders, which can later on be converted into the desired functionality (Scheme 1B).^12^ Therefore, Matteson homologation is increasingly used in natural product synthesis^13^ and for the synthesis of pharmaceuticals,^14^ whereby it also attracted the attention of our group.^15^

**Fig. 1:**
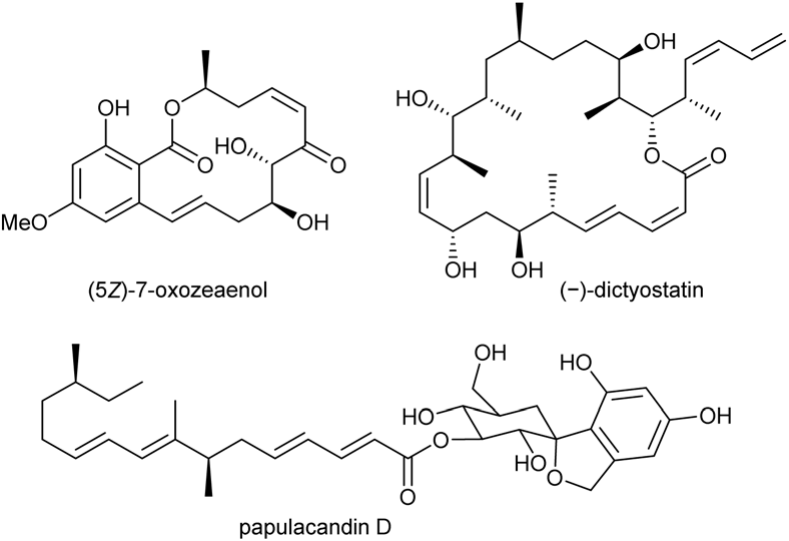
Unsaturated natural products.

While saturated alkyl chains can be easily obtained using the Matteson protocol, the stereoselective introduction of double bonds or even conjugated double bonds, such as those found in the natural products like (5*Z*)-7-oxozeaenol,^16^ papulacandin D^17^ and (−)-dictyostatin^18^ (Fig. 1) is not possible.

To solve this problem, cross-coupling reactions offer a good alternative to extend the carbon chain by introducing a double bond. In this context, zirconium is the metal of choice for this approach because it provides readily reactive vinyl nucleophiles *via* hydrozirconation of alkynes, has functional group tolerance and is non-toxic (Scheme 1C).^19^ In addition, Srebnik *et al.* were able to demonstrate the compatibility of these two different metals in the same molecule by hydrozirconation of vinyl boranes^20^ and vinyl boronic esters (Scheme 1D).^21^ The vinyl zirconium reagents can be functionalised either by reactions with electrophiles such as protons, halides and acid chlorides^22^ or by Ni- or Pd-catalysed Negishi couplings (Scheme 1C).^23^ Negishi couplings allow for the introduction of various aryl,^24^ vinyl,^25^ allyl^26^ and even alkyl groups.^27^ Therefore, these reactions have become common tools for the synthesis of natural products.^28^

Herein, we report an application of this approach using selective functionalisations of homopropargyl boronic esters *via* hydrozirconation and subsequent Negishi couplings.

## Results and discussion

**Scheme 2:**
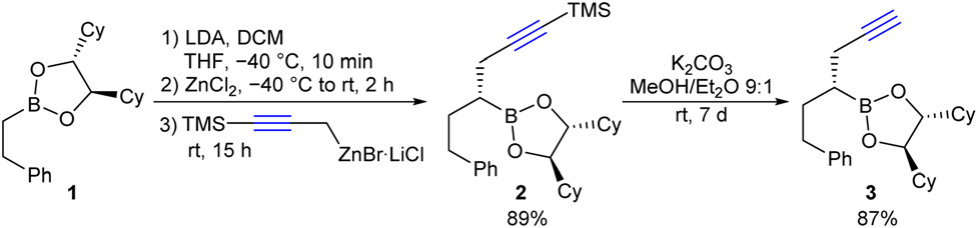
Synthesis of homopropargyl boronic ester 3.

Initially, the homopropargyl boronic ester 3 required for hydrozirconation was synthesised as a model compound starting from boronic ester 1 (Scheme 2). For this purpose, 1 was first reacted in a Matteson homologation with LiCHCl_2_ and the α-chloroboronic ester formed was immediately subjected to a nucleophilic substitution with the TMS-protected propargylzinc reagent.^15*f*^ The primary formed boronic ester 2 was then deprotected with K_2_CO_3_ in methanol/ether, which proved to be the method of choice (see SI).^29^ The addition of diethyl ether was necessary because of the poor solubility of boronic ester 2 in methanol. It should be mentioned that commonly used fluoride-containing cleavage reagents such as TBAF or KF were not compatible with the boronic ester functionality because the fluoride binds to boron rather than silicon, resulting in at least partial protodeboration.

**Scheme 3:**
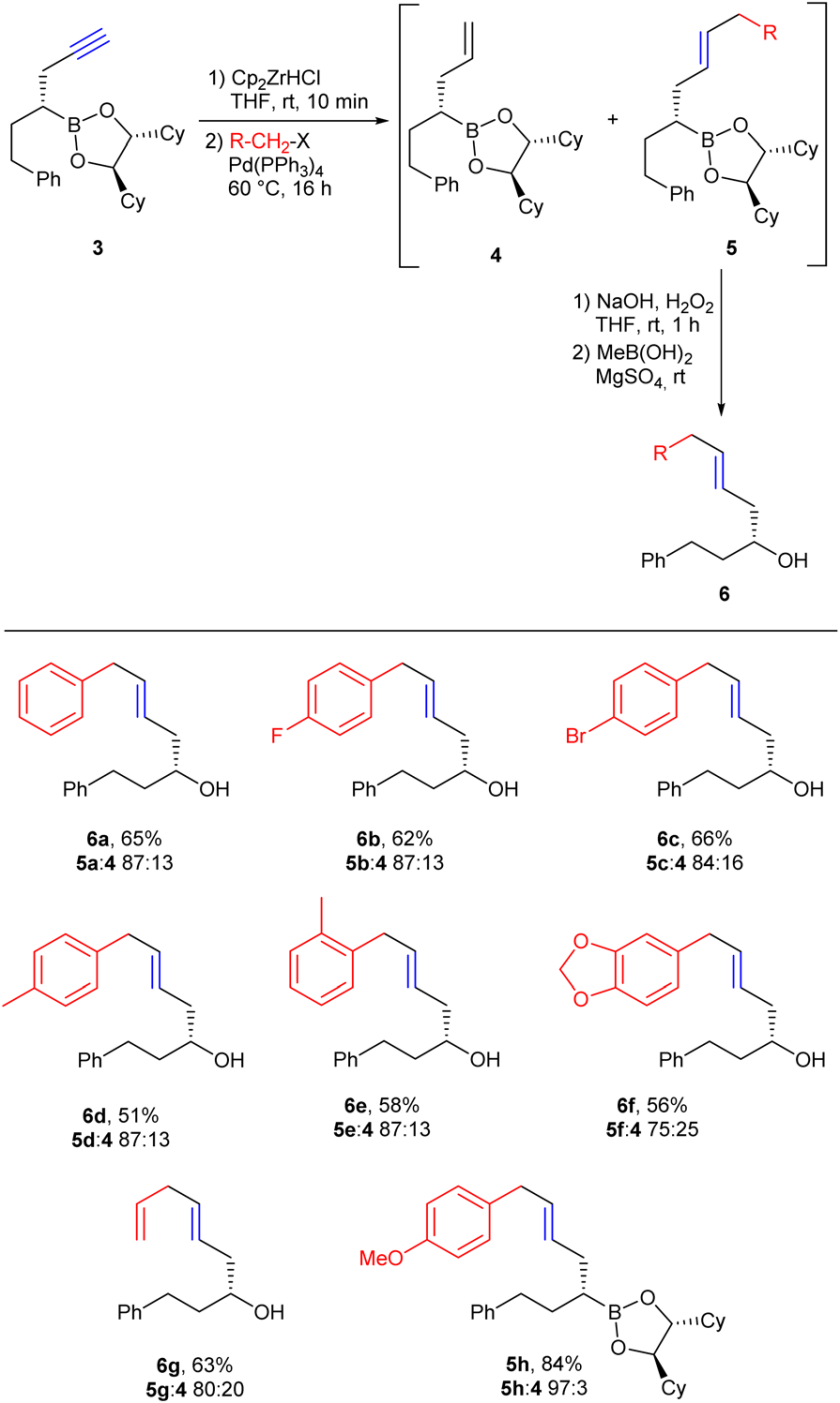
Negishi couplings of the vinyl zirconium reagent prepared from 3 with benzyl and allyl halides.

Starting from 3, Negishi couplings with benzyl halides and allyl bromide were first investigated (Scheme 3).^30^ The hydrozirconation of 3 went smoothly, but no complete turnover could be observed in subsequent cross couplings. Thus, after workup, the desired coupling product 5 was usually contaminated with allyl-substituted boronic ester 4.^12^ It originates from the vinyl zirconium intermediate formed *via* hydrozirconation of 3, which did not react in the following cross coupling, and was thus hydrolysed during aqueous workup. Changes in the reaction conditions as minimisation of the excess of Schwartz reagent to 1.0 eq. or increasing the amount of benzyl halide as well as extended reaction times did not improve the turnover of the cross-coupling reaction. Since 4 could not be separated from the desired substitution product 5 due to almost equal chromatographic retention, the mixture of boronic esters 4 and 5 was directly oxidised to the corresponding alcohols, which could be easily separated by column chromatography. Methylboronic acid was added after the workup to remove the chiral auxiliary.^15*a*^ The corresponding chiral methylboronic ester formed can easily be separated and reused in Matteson reactions. The yield given in Scheme 3 for the desired alcohol 6 corresponds to the isolated and purified alcohol after 4 steps.

The ratio 4 : 5 was determined by ^1^H NMR spectroscopy of the crude product mixture. In addition to the unsubstituted benzyl residue (6a), benzyl residues containing both electron-withdrawing (6b and 6c) and electron-donating (6g, 6e, 6f and 5h) substituents were also tested. The electronic properties of the respective substituents showed no significant influence on the results of the reactions, nor did different positions of the substituents on the aromatic ring. If allyl bromide was used instead of a benzyl halide, 1,4-dienes (6g) became available. In the case of the *p*-methoxy-substituted derivative 5h, the boronic ester could be obtained in high yield by chromatography, since 4 was formed only in trace amounts in this case.

**Scheme 4:**
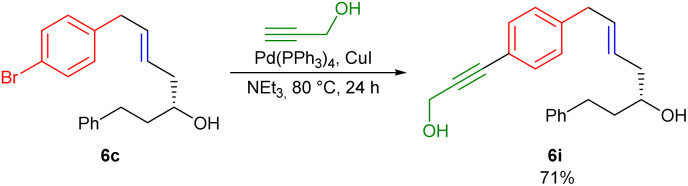
Subsequent Sonogashira coupling of 6c.

As illustrated with 6c, *p*-bromobenzyl bromide reacts regioselectively at the benzylic position, which allows further modifications of the aryl bromide, *e.g.* by Sonogashira coupling (Scheme 4).

**Scheme 5:**
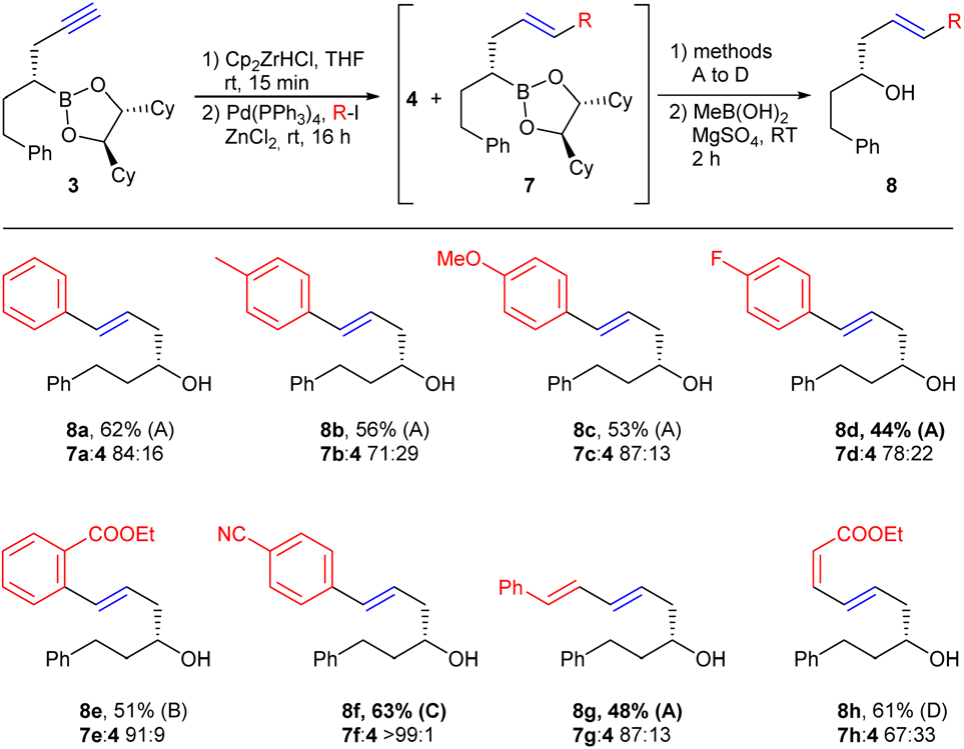
Negishi couplings of the vinyl zirconium reagent prepared from 3 with aryl- and vinyliodides. Method A: NaOH, H_2_O_2_, THF, H_2_O, rt, 1 h; method B: Na_2_CO_3_, H_2_O_2_, THF, H_2_O, rt, 1 h; method C: NaOH, NaBO_3_·4H_2_O, THF, H_2_O, rt, 1 h; method D: NaBO_3_·4H_2_O, THF, Sørensen phosphate buffer pH 8, rt, 5 h.

Although benzyl halides are obviously more reactive than aryl halides, these can still be used in Negishi couplings (Scheme 5). However, since the vinyl zirconium reagent formed *in situ* was not reactive enough to enable coupling with vinyl and aryl halides, transmetallation to zinc was necessary in this case.^23,24*b*^ The addition of zinc chloride should take place last, since transmetallation in the absence of a Pd catalyst leads to a partial decomposition of the respective boronic ester.

Under the optimised conditions, Negishi couplings were performed with different aryl iodides, which carried electron-donating and -withdrawing groups in *ortho* and *para* position, respectively, as well as an electron-rich (8g) and an electron-poor vinyl iodide (8h). As already observed in the synthesis of boronic esters 5, allylboronic ester 4 was also obtained as a by-product in most cases. The products could be easily separated after oxidation by flash chromatography, whereby also here the yields given refer to the complete reaction sequence (from 3). Depending on the substituents on the aromatic ring, slightly different oxidation protocols were used. Interestingly, in the case of the nitrile-substituted derivative 8f, complete turnover and no formation of 4 was observed.

Due to the highly regioselective *syn*-hydrozirconation, a large number of (*E*)-configured unsaturated alcohols and boronic esters could be obtained without any problems. However, direct access to the corresponding (*Z*)-alkenes is not possible *via* this protocol. However, this can be achieved by reversing the order of the synthetic steps. If the substituent is introduced at the alkyne level *via* Sonogashira coupling, the subsequent hydrozirconation of 9 represents an alternative to the Lindlar hydrogenation of internal alkynes (Table 1). For easier purification, the boronic esters 10 were again oxidised to the corresponding alcohols 11.

**Table 1:** Synthesis of (*Z*)-alkenes *via* Sonogashira coupling, hydrozirconation and oxidation

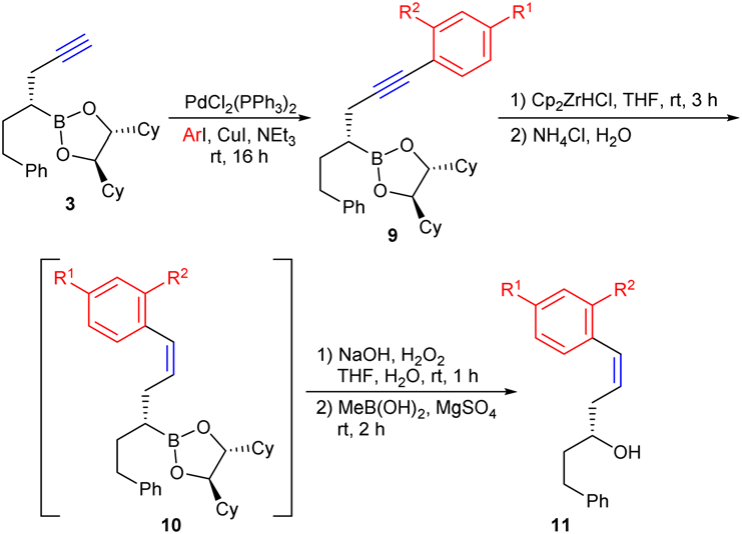							
Entry	R^1^	R^2^	9	9 [yield]	10 [yield]	11 [yield]	Overall yield
1	H	H	9a	99%	88%	66%	57% 11a
2	Me	H	9b	100%	84%	76%	64% 11b
3	OMe	H	9c	100%	71%	90%	64% 11c
4	F	H	9d	97%	87%	76%	64% 11d
5	H	OMe	9e	78%	39% (62%)^a^	n.i.^c^	30% 10e (48%)^a^
6	NO_2_	H	9f	92%	—b	—	92% 9f

a Base on recovered starting material.

b No conversion.

c Not investigated.

As shown in Table 1, a number of different aryl substituents, both with electron-donating and -withdrawing groups, were introduced *via* the Sonogashira coupling, but no significant influence on the yields was observed. The introduction of an *ortho*-substituent was possible without problems, whereby the steric interaction between the *o*-MeO group and the zirconocene group during hydrozirconation only leads to an incomplete transformation. Here, unreacted alkyne 9e could be recovered, since 9e and 10e could be separated by flash chromatography.

Only in case of 9f no hydrozirconation was observed, which might be caused by the strong electron-withdrawing effect of the nitro group, which obviously makes the alkyne too electron-poor for coordinating to the Schwartz reagent. Interestingly, however, the nitro group was also not reduced.

Therefore, by simply changing the order of hydrozirconation and cross coupling both, (*E*)- and (*Z*)-alkenes become available.

**Scheme 6:**
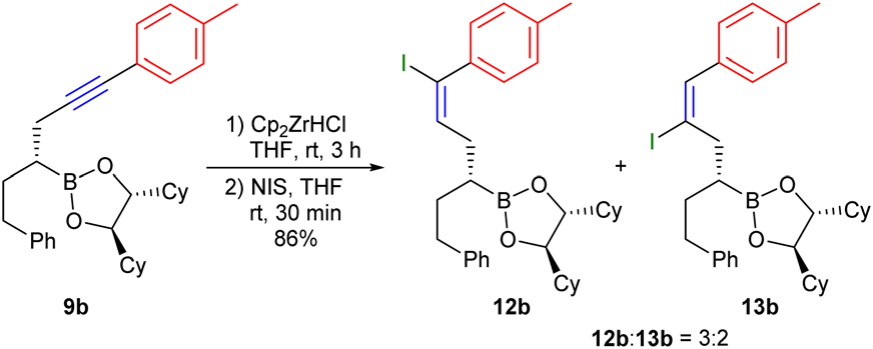
Investigation of the regioselectivity of hydrozirconation of 9b.

Concerning the yields obtained, the presented method will certainly not be able to compete with Lindlar hydrogenation, but it would be an interesting approach if the hydrozirconation is regioselective and the formed disubstituted vinyl zirconium intermediate can be converted into triple-substituted alkenes *via* cross couplings. Therefore, the hydrozirconation was investigated in more detail using the aryl-substituted alkyne 9b. The formed vinyl zirconium compound was converted with *N*-iodosuccinimide into the corresponding vinyl iodide.^31^ According to the mechanism of hydrozirconation of internal alkynes, as proposed by Schwartz *et al.*,^32^ Cp_2_ZrHCl was used in excess to achieve better regioselectivity. But nevertheless, in case of 9b, the regioisomeric iodides 12b and 13b were obtained only as a 3 : 2 mixture, but in a high yield (Scheme 6). Therefore, optimisations of regioselectivity could also open up new synthetic possibilities here.

## Conclusions

In summary, we have shown that the hydrozirconation of homopropargyl boronic esters enables the selective functionalisation of the carbon chain established by Matteson homologation. The respective organozirconium reagents can react with electrophiles such as halides or participate in Negishi couplings with either benzyl halides or aryl and vinyl iodides. Further applications of this method in total syntheses are currently under investigation.

## Conflicts of interest

There are no conflicts to declare.

## Supplementary Material

RA-016-D6RA02170K-s001

## Data Availability

Supplementary information (SI): copies of ^1^H and ^13^C NMR spectra and experimental details. See DOI: https://doi.org/10.1039/d6ra02170k.

## References

[cit1] Staunton J., Weissman K. J. (2001). Polyketide biosynthesis: a millennium review. Nat. Prod. Rep..

[cit2] Paterson I., Lam N. Y. S. (2018). Challenges and discoveries in the total synthesis of complex polyketide natural products. J. Antibiot..

[cit3] Steinmetz H., Gerth K., Jansen R., Dehn R., Reinecke S., Kirschning A., Müller R. (2011). Elansolid A, a unique macrolide antibiotic from *Chitinophaga sancti* isolated as two stable atropisomers. Angew. Chem., Int. Ed..

[cit4] Roush W. R., Palkowitz A. D., Ando K. (1990). Acyclic diastereoselective synthesis using tartrate ester-modified crotylboronates. Double asymmetric reactions with α-methyl chiral aldehydes and synthesis of the C(19)-C(29) segment of rifamycin S. J. Am. Chem. Soc..

[cit5] Matteson D. S. (1989). α-Halo boronic esters: intermediates for stereodirected synthesis. Chem. Rev..

[cit6] Matteson D. S., Majumdar D. (1980). α-Chloro boronic esters from homologation of boronic esters. J. Am. Chem. Soc..

[cit7] Matteson D. S., Sadhu K. M., Peterson M. L. (1986). 99% Chirally selective synthesis via pinanediol boronic esters: insect pheromones, diols, and an amino alcohol. J. Am. Chem. Soc..

[cit8] Matteson D. S., Yang J. J. (1997). Stereoselective chain extension
of (*R*,*R*)- or (*S*,*S*)-1,2-dicyclohexylethane-1,2-diol trityloxymethylboronate to compounds having three stereogenic centers. Tetrahedron: Asymmetry.

[cit9] Hiscox W. C., Matteson D. S. (2000). Asymmetric synthesis of the Japanese beetle pheromone via boronic esters. J. Organomet. Chem..

[cit10] Matteson D. S., Peterson M. (1987). Synthesis of L-(+)-ribose via (*S*)-pinanediol (α*S*)-α-bromoboronic esters. J. Org. Chem..

[cit11] Matteson D. S., Beedle E. C. (1987). A directed chiral synthesis of amino acids from boronic esters. Tetrahedron Lett..

[cit12] Andler O., Kazmaier U. (2021). Allylzinc reagents: versatile nucleophiles in Matteson homologations. Org. Lett..

[cit13] Matteson D. S., Man H. W., Ho O. C. (1996). Asymmetric synthesis of stegobinone via boronic ester chemistry. J. Am. Chem. Soc..

[cit14] Gorovoy A. S., Gozhina O., Svendsen J. S., Tetz G. V., Domorad A., Tetz V. V., Lejon T. (2013). Syntheses and anti-tubercular activity of β-substituted and α,β-disubstituted peptidyl β-aminoboronates and boronic acids. J. Pept. Sci..

[cit15] Gorges J., Kazmaier U. (2018). Matteson homologation-based total synthesis of lagunamide A. Org. Lett..

[cit16] Ayers S., Graf T. N., Adcock A. F., Kroll D. J., Matthew S., Carcache De Blanco E. J., Shen Q., Swanson S. M., Wani M. C., Pearce C. J., Oberlies N. H. (2011). Resorcylic acid lactones with cytotoxic and NF-κB inhibitory activities and their structure-activity relationships. J. Nat. Prod..

[cit17] Traxler P., Gruner J., Auden J. A. L. (1977). Papulacandins, a new family of antibiotics with antifungal activity – I. fermentation, isolation, chemical and biological characterization of papulacandins A, B, C, D and E. J. Antibiot..

[cit18] Pettit G. R., Cichacz Z. A., Gao F., Boyd M. R., Schmidt J. M. (1994). Isolation and structure of the cancer cell growth inhibitor dictyostatin 1. J. Chem. Soc. Chem. Commun..

[cit19] (d) Song Z. and Takahashi T., Hydrozirconation of Alkenes and Alkynes, Elsevier Ltd, 2014

[cit20] Zheng B., Srebnik M. (1993). Preparation and selective cleavage reactions of boron-zirconium 1,1-bimetalloalkanes. Tetrahedron Lett..

[cit21] Zheng B., Srebnik M. (1994). Synthesis of a new class of bidentate Lewis acids based on boronic esters and zirconocene. J. Organomet. Chem..

[cit22] Hart D. W., Schwartz J. (1974). Hydrozirconation. Organic synthesis via organozirconium intermediates. Synthesis and rearrangement of alkylzirconium(IV) complexes and their reaction with electrophiles. J. Am. Chem. Soc..

[cit23] Negishi E. I., Okukado N., King A. O., Van Horn D. E., Spiegel B. I. (1978). Selective carbon-carbon bond formation via transition metal catalysts. 9. Double metal catalysis in the cross-coupling reaction and its application to the stereo- and regioselective synthesis of trisubstituted olefins. J. Am. Chem. Soc..

[cit24] Negishi E. I., Van Horn D. E. (1977). Selective carbon-carbon bond formation via transition metal catalysis. 4. A novel approach to cross-coupling exemplified by the nickel-catalyzed reaction of alkenylzirconium derivatives with aryl halides. J. Am. Chem. Soc..

[cit25] Okukado N., Van Horn D. E., Klima W. L., Negishi E. I. (1978). A highly stereo-, regio-, and chemoselective synthesis of conjugated dienes by the palladium-catalyzed reaction of (*E*)-1-alkenylzirconium derivatives with alkenyl halides. Tetrahedron Lett..

[cit26] Hayasi Y., Riediker M., Temple J. S., Schwartz J. (1981). Ligand control in palladium-catalyzed coupling reactions between organozirconium compounds and allylic species. Tetrahedron Lett..

[cit27] Wiskur S. L., Korte A., Fu G. C. (2004). Cross-couplings of alkyl electrophiles under “ligandless” conditions: Negishi reactions of organozirconium reagents. J. Am. Chem. Soc..

[cit28] Riediker M., Schwartz J. (1981). A new synthesis of 25-hydroxycholesterol. Tetrahedron Lett..

[cit29] Navickas V., Rink C., Maier M. E. (2011). Synthetic studies towards leiodermatolide: rapid stereoselective syntheses of key fragments. Synlett.

[cit30] Xu S., Holst H. M., McGuire S. B., Race N. J. (2020). Reagent control enables selective and regiodivergent opening of unsymmetrical phenonium ions. J. Am. Chem. Soc..

[cit31] O'Rourke N. F., Davies K. A., Wulff J. E. (2012). Cascading radical cyclization of bis-vinyl ethers: mechanistic investigation reveals a 5-exo/3-exo/retro-3-exo/5-exo pathway. J. Org. Chem..

[cit32] Hart D. W., Blackburn T. F., Schwartz J. (1975). Hydrozirconation. III. Stereospecific and regioselective functionalization of alkylacetylenes via vinylzirconium(IV) intermediates. J. Am. Chem. Soc..

