# Supplementary material for: Functionalisation of homopropargyl boronic esters via hydrozirconation followed by Pd-catalysed cross coupling reaction
Source: RSC Adv. 2026 Mar 24;16(18):16310–5. doi: 10.1039/d6ra02170k (PMC13010875; doi:10.1039/d6ra02170k)
Supplement: RA-016-D6RA02170K-s001 [file RA-016-D6RA02170K-s001.pdf]

Supplementary Information (SI) for RSC Advances.
This journal is © The Royal Society of Chemistry 2026

Supplementary information

Functionalisation of Homopropargyl Boronic Esters via Hydrozirconation followed by Pd-catalysed Cross-Coupling Reaction

Patrick Schäfer[a] and Uli Kazmaier[a]*

Organic Chemistry I, Saarland University, Campus, Building C 4.2

D-66123 Saarbrücken, Germany

E-Mail: u.kazmaier@mx.uni-saarland.de

Table of contents

General information S2

General procedures S3

Test reactions and optimizations S5

Deprotection of alkyne 2 .... S5

Synthesis of compounds S6

Copies of NMR spectra S28

References S63

General information

All air and moisture sensitive reactions were conducted in dried glassware (>100 °C) under a nitrogen atmosphere if not stated otherwise. Dried solvents were distilled prior to use: THF was distilled from sodium/benzophenone and *N*,*N*-diisopropylamine was dried over CaH_2 before distillation. All other anhydrous solvents were purchased from *Thermo Scientific* and stored under nitrogen atmosphere over molecular sieves. Pentane and ethyl acetate were distilled prior to use. Column chromatography was performed using the *Büchi Pure C-815 Flash* system with *Teledyne Isco RediSep Rf Silver Silica* normal-phase columns (30-70 µm) with mixtures of cyclohexane and ethyl acetate as eluent. Reverse-phase chromatography was performed using the *Büchi Reveleris Prep* system with *Büchi FlashPure Select C18* 30 µm spherical cartridges with mixtures of water and acetonitrile as eluent. For analytical TLC, the precoated silica-gel plates *Polygram Sil G/UV*$_{254}$ by *Macherey-Nagel* were used. Detection was accomplished with UV light (λ = 254 nm) and cerium(IV)/ammonium molybdate solution. ^{1}H-, ^{13}C- and ^{19}F-NMR spectra were measured on a *Bruker Advance II* 400 MHz spectrometer (^{1}H: 400 MHz, ^{13}C: 100 MHz, ^{19}F: 400 MHz), a *Bruker Advance I* 500 MHz spectrometer (^{1}H: 500 MHz, ^{13}C: 125 MHz) or a *Bruker Advance Neo* 500 MHz spectrometer (^{1}H: 500 MHz, ^{13}C: 125 MHz). Chemical shifts (δ) are reported in parts per million (ppm) relative to the internal solvent signal (7.26 ppm for $CDCl_3$ as NMR solvent) for ^{1}H- and ^{13}C-NMR and to a tertiary reference (−61.62 ppm for benzotrifluoride or −75.39 ppm for trifluoroacetic acid) for ^{19}F-NMR. The peaks were assigned using ^{1}H,^{1}H-COSY, ^{1}H,^{13}C-HSQC and ^{1}H,^{13}C-HMBC spectra. NMR spectra were evaluated using ACD Labs NMR Processor Version 12.01 or *Bruker Top Spin* Version 4.3.0. Mass spectra were recorded with a *Finnigan MAT95* spectrometer (quadrupol) using the CI technique or an *Orbitrap Q* exactive mass spectrometer, equipped with a heated ESI source and a quadrupole-orbitrap coupled mass detector and an *Ultimate3000* HPLC (Thermo Finnigan, San Jose, CA). The MS detection was carried out at a spray voltage of 3.8 kV in positive ionisation mode, a nitrogen sheath gas pressure of $4.0 \cdot 10^{-5}$ Pa, an auxiliary gas pressure of $1.0 \cdot 10^{-5}$ Pa and a capillary temperature of 300 °C. All samples were injected by autosampler with an injection volume of 15 µL. A *RP Nucleoshell Phenyle-hexyle*® (50-2, 3.0µm) column (Macherey-Nagel GmbH, Düren, Germany) was used as stationary phase. The solvent system consisted of formic acid 0.1% (A) and acetonitrile with formic acid 0.1% (B). HPLC method: flow rate 600 µL/min. The percentage of B started at an initial of 1%, was kept at 1% for 1.5 min, then rapidly increased to 100 % during 4.0 min, then kept at 100% until 1.5 min and flushed back to the initial 1 %. Xcalibur software was used for data acquisition and plotting. Optical rotations were measured with a *Krüss P8000-T* polarimeter in a thermostat-controlled cuvette (20.0 ± 0.1 °C) using a sodium vapour lamp (λ = 589 nm) as radiation source. $[\alpha]_D^{20}$ values are given in 10^{-1} deg·cm^2·g^{-1} and c is given in g·cm^{-3}.

General procedures

GP-1: Matteson homologation

To a solution of *N*,*N*-diisopropylamine (1.35 eq.) in anhydrous THF (0.2 ml/mmol) was added *n*-BuLi (1.25 eq., 1.6 M in hexanes) dropwise at a temperature between −40 °C and −50 °C. The reaction mixture was stirred for 20 min at room temperature. The freshly prepared LDA solution was added slowly at a temperature between −40 °C and −50 °C to a solution of the boronic ester (1.0 eq.) and anhydrous DCM (3.0 eq.) in anhydrous THF (1.4 ml/mmol).[1] After 10 min of stirring at the same temperature, a solution of $ZnCl_2$ (2.0–3.0 eq., flame dried *in vacuo*) in anhydrous THF (0.6 ml/mmol $ZnCl_2$) was added and the reaction was stirred for 2 h at room temperature before adding the nucleophile solution according to the respective variant.

After completion, saturated NH_4Cl solution was added to the reaction mixture. The biphasic mixture was stirred for 5 min before separating the phases. The aqueous phase was extracted twice with pentane. The combined organic phases were dried over Na_2SO_4 and filtered. The solvent was removed under reduced pressure. The crude product was purified by column chromatography.

Variant A) Substitution with organozinc reagent

Organozinc reagent was prepared by suspending LiCl (1.1 eq., flame dried *in vacuo*) and zinc dust (2.0 eq.) in anhydrous THF (1.0 M) and adding 1,2-dibromoethane (2 mol-%). The reaction mixture is carefully heated with a heat gun to gentle boiling. After cooling to room temperature, trimethylsilyl chloride (5 mol-%) is added and the reaction mixture is again heated with a heat gun to gentle boiling. After cooling to room temperature, a solution of the alkyl bromide (1.0 eq.) in anhydrous THF (1.0 M) was added dropwise. An exothermic reaction could be observed. After 1 h excess zinc dust was allowed to settle and the supernatant solution was directly used in the next step. The concentration of the organozinc solution was determined by iodometric titration.[1]

The reaction mixture resulting from homologation was cooled to 0 °C and the freshly prepared organozinc solution was added dropwise. The reaction mixture was stirred at room temperature until completion indicated by ^{1}H-NMR analysis.

Variant B) Substitution with Grignard reagent

The reaction mixture was cooled to 0 °C and the solution of the Grignard reagent (2.5 eq.) was added slowly. The reaction mixture was stirred at room temperature until completion indicated by ^{1}H-NMR analysis.

Variant C) Substitution with sodium alcoholate

To a suspension of sodium hydride (1.3 eq., 60% in mineral oil) in anhydrous THF (0.5 ml/mmol) and anhydrous DMSO (1.3 ml/mmol) was added the respective alcohol (1.4 eq.). the reaction mixture was stirred for 6 – 7 h at room temperature.

The reaction mixture resulting from homologation was cooled to 0 °C and the alcoholate solution was added slowly. The reaction mixture was stirred at room temperature until completion indicated by ^{1}H-NMR analysis.

---

[1] The LDA solution should be added very carefully by direct addition to the reaction mixture. If this is done too fast, the reaction mixture turns from non-transparently dark brown to black. In this case, the reaction mixture should be discarded. If done correctly, the reaction mixture turns yellow to orange. When adding the $ZnCl_2$ solution, the intensity decreases slightly and returns after 2 h of stirring at room temperature.

GP-2: Hydrozirconation and Negishi cross-coupling with benzyl halogenides

Boronic ester (1.0 eq.) and Schwartz reagent (1.0 eq.) were suspended in anhydrous THF (0.2 M) in the absence of light. The reaction mixture was stirred for 10 min until the reaction mixture became transparent indicating complete hydrozirconation. Then, $Pd(PPh_3)_4$ (3 mol-%) and benzyl halide (1.0 eq.) were added. The reaction mixture was stirred at 60 °C until completion indicated by ^{1}H-NMR analysis.

After cooling to room temperature, the solvent was removed under reduced pressure. The residue was dissolved in pentane/ethyl acetate (9:1 v/v) and filtered over Celite. The Celite was rinsed with pentane. The solvent was removed under reduced pressure and the residue was purified by column chromatography.

GP-3: Hydrozirconation and Negishi cross-coupling with aryl iodides

Boronic ester (1.0 eq.) and Schwartz reagent (1.0 eq.) were suspended in anhydrous THF (0.75 M) in the absence of light. The reaction mixture was stirred for 10 min until the reaction mixture became transparent indicating complete hydrozirconation. Then, $Pd(PPh_3)_4$ (6.6 mol-%), aryl iodide (2.0 eq.) and a solution of $ZnCl_2$ (1.3 eq., flame dried *in vacuo*) in anhydrous THF (1.95 M) were added. The reaction mixture was stirred at room temperature until completion indicated by ^{1}H-NMR analysis.

After completion, water was added to the reaction mixture and the phases were separated. The aqueous phase was extracted three times with Et_2O. The combined organic phases were washed with water. The organic phase was dried over Na_2SO_4 and filtered. The solvent was removed under reduced pressure and the crude product was purified by column chromatography.

GP-4: Oxidation of boronic esters to alcohols

At 0 °C H_2O_2 (5.0 eq., 33% in water) and a solution of NaOH (5.0 eq.) in water (2 ml/mmol) were added to a solution of the boronic ester (1.0 eq.) in THF (2 ml/mmol). The reaction mixture was warmed to room temperature and stirred at the same temperature until completion indicated by TLC. The reaction mixture was then washed with brine and the aqueous phase was extracted three times with Et_2O. the combined organic phases were dried over Na_2SO_4 and filtered. The solvent was removed under reduced pressure. To improve separation of the alcohol and DICHED, the crude product was diluted in Et_2O (5 ml/mmol) and methylboronic acid (1.2 eq.) was added with an excess of $MgSO_4$ and the reaction mixture was stirred for 2 h at room temperature. The reaction mixture was filtered and the solvent was removed under reduced pressure. The crude product was purified by column chromatography.

GP-5: Sonogashira cross-coupling

Boronic ester (1.0 eq.), $PdCl_2(PPh_3)_2$ (0.1 eq.), CuI (0.2 eq.) and aryl iodide (5.0 eq.) were dissolved in NEt_3 (0.15 M) under an argon atmosphere. The reaction mixture was stirred at room temperature until completion indicated by ^{1}H-NMR analysis.

The reaction mixture was diluted with pentane and filtered over Celite. The Celite was rinsed with pentane. The solvent was removed under reduced pressure. The crude product was purified by column chromatography.

Test reactions and optimisations

Deprotection of alkyne 2

Since the direct introduction of the unprotected propargyl substituent via Matteson homologation was not successful, the TMS protected substituent was used. To afford the terminal alkyne **3**, **2** had to be deprotected first. Fluoride-containing reagents as TBAF or KF are not compatible to the boronic ester functionality because the fluoride rather binds to the boron rather than the silicon leading to at least partial protodeboration.[2,3] The same could be observed when trying deprotection with TBAF (entry 1).[4] The attack on the boronic ester functionality was indicated by the formation of DICHED during the reaction. So, different deprotection conditions were tested. First, deprotection with potassium carbonate (entry 2)[5] was investigated which showed to be the best method despite the relatively long reaction time because of complete conversion and a high isolated yield (87%). When sodium methylate was used as base, no reaction could be observed (entry 3).[6] The conditions in entry 4[7] offered the shortest reaction time but also a low isolated yield.

Tab. S1. Tested conditions for the deprotection of alkyne **2**.

Entry	Reagent	Solvent	Temperature	Time	Commentary
**1**	TBAF	THF	RT	1 h	Attack on boronic ester
**2**	K_2CO_3	$MeOH/Et_2O$ 9:1	RT	7 d	Complete conversion
**3**	NaOMe	$MeOH/Et_2O$ 9:1	RT	24 h	No reaction
**4**	$AgNO_3$, 2,6-lutidine	$THF/H_2O/EtOH$ 1:1:1	RT	1 h	Complete conversion, low isolated yield (24%)

Synthesis of compounds

((*R*)-4-((4*R*,5*R*)-4,5-Dicyclohexyl-1,3,2-dioxaborolan-2-yl)-6-phenylhex-1-yn-1-yl)trimethylsilane (2)

According to GP-1, 1.72 ml (1.22 g, 12.1 mmol) *N*,*N*-diisopropylamine, 6.98 ml (11.2 mmol, 1.6 M) *n*-butyllithium and 1.79 ml anhydrous THF were used for the LDA solution, 2.44 g (17.9 mmol) zinc chloride and 10.7 ml anhydrous THF were used for the $ZnCl_2$ solution and 3.04 g (8.93 mmol) boronic ester **1**,[8] 1.73 ml (2.28 g, 26.8 mmol) anhydrous DCM and 12.5 ml anhydrous THF were used for the homologation.

The organozinc reagent was prepared according to variant A using 2.10 g (49.5 mmol) LiCl, 5.88 g (90.0 mmol) zinc dust, 78.0 µl (0.90 mmol) 1,2-dibromoethane, 288 µl (2.25 mmol) trimethylsilyl chloride, 45.0 ml anhydrous THF and a solution of 7.35 ml (45.0 mmol) (3-bromoprop-1-yn-1-yl)trimethylsilane in 45.0 ml anhydrous THF. The reaction mixture was worked up after 15 h. After column chromatography (SiO_2, CyH/EE 0-3%), the product **5** (3.65 g, 7.86 mmol, 88%) was obtained as a colourless oil. R_f (**2**) = 0.34 (pentane/EE 97:3). $[\alpha]_D^{20}$ = +49.4 (c = 1.0, $CHCl_3$).

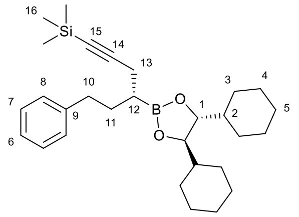

^{1}H-NMR (400 MHz, $CDCl_3$): δ = 7.30–7.26 (m, 2 H, 7-H), 7.21–7.16 (m, 3 H, 6-H, 8-H), 3.89–3.86 (m, 2 H, 1-H), 2.66 (m, 2 H, 10-H), 2.46–2.33 (m, 2 H, 13-H), 1.88–1.61 (m, 12 H, 3-Ha, 3-Hb, 4-Ha, 4-Hb, 5-Ha 11-H), 1.37–0.97 (m, 13 H, 2-H, 3-Hc, 3-Hd, 4-Hc, 4-Hd, 5-Hb, 12-H), 0.14 (s, 9 H, 16-H) ppm.

^{13}C-NMR (100 MHz, $CDCl_3$): δ = 142.8 (C-9), 128.4 (C-8), 128.2 (C-7), 125.6 (C-6), 107.2 (C-14), 84.7 (C-1), 83.5 (C-15), 43.0 (C-2), 34.9 (C-10), 32.3 (C-11), 28.3 (C-3a), 27.4 (C-3b), 26.5 (C-5), 26.0 (C-4a), 25.9 (C-4b), 21.1 (C-13), 0.2 (C-16) ppm.

The signal of C-12 could not be detected.

HRMS (CI) m/z calcd for $C_{29}H_{45}BO_2Si$ $[M]^+$: 464.3282, found: 464.3312.

(4*R*,5*R*)-4,5-Dicyclohexyl-2-((*R*)-1-phenylhex-5-yn-3-yl)-1,3,2-dioxaborolane (3)

To a solution of 2.58 g (5.55 mmol, 1.0 eq.) boronic ester **2** in 76.9 ml methanol/diethyl ether (9:1 v/v, 0.07 M) was added 1.53 g (11.1 mmol, 2.0 eq.) K_2CO_3. The reaction mixture was stirred at room temperature for 7 d.

To the reaction mixture, saturated NH_4Cl solution and diethyl ether were added and the phases were separated. The aqueous phase was extracted twice with diethyl ether. The combined organic phases were dried over Na_2SO_4 and filtered. The solvent was removed under reduced pressure and the residue was purified by column chromatography (SiO_2, CyH/EE 0-5%). The product **6** (1.89 g, 4.81 mmol, 87%) was obtained as colourless oil. R_f (**3**) = 0.30 (pentane/EE 97:3). $[\alpha]_D^{20}$ = +49.4 (c = 1.0, $CHCl_3$).

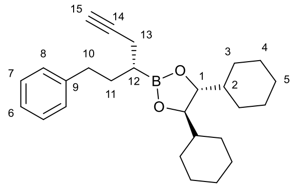

^{1}H-NMR (400 MHz, $CDCl_3$): δ = 7.29–7.24 (m, 2 H, 7-H), 7.23–7.16 (m, 3 H, 6-H, 8-H), 3.90–3.87 (m, 2 H, 1-H), 2.72–2.60 (m, 2 H, 10-H), 2.41–2.29 (m, 2 H, 13-H), 1.93 (t, *J* = 2.6 Hz, 1 H, 15-H), 1.94–1.66 (m, 12 H, 3-Ha, 3-Hb, 4-Ha, 4-Hb, 5-Ha, 11-H), 1.42–1.00 (m, 13 H, 2-H, 3-Hc, 3-Hd, 4-Hc, 4-Hd, 5-Hb, 12-H) ppm.

^{13}C-NMR (100 MHz, $CDCl_3$): δ = 142.7 (C-9), 128.4 (C-8), 128.2 (C-7), 125.6 (C-6), 84.3 (C-14), 85.5 (C-1), 68.6 (C-15), 43.0 (C-2), 35.0 (C-10), 32.4 (C-11), 28.4, 27.5, 26.5, 25.9, 19.8 (C-13) ppm.

The signal of C-12 could not be detected.

HRMS (CI) m/z calcd for $C_{26}H_{38}BO_2$ $[M+H]^+$: 393.2965, found: 393.2974.

(4*R*,5*R*)-4,5-dicyclohexyl-2-((*R*,*E*)-7-(4-methoxyphenyl)-1-phenylhept-5-en-3-yl)-1,3,2-dioxaborolane (5h)

According to GP-2, 113 mg (288 µmol) boronic ester **3**, 82.0 mg (316 µmol, 1.1 eq) Schwartz reagent and 1.44 ml anhydrous THF were used for the hydrozirconation. 10.0 mg (8.63 µmol) $Pd(PPh_3)_4$, 39,2 µl (45.1 mg, 288 µmol) 4-methoxybenzyl chloride and additional 0.58 ml anhydrous THF were used for the Negishi coupling. The rection was worked up after 24 h. After column chromatography (SiO_2, CyH/EE 0-5%), the product **5h** (131 mg, 242 µmol, 84%) was obtained as a colourless oil. R_f (**5h**) = 0.32 (pentane/EE 95:5). $[\alpha]_D^{20}$ = +17.9 (c = 1.0, $CHCl_3$).

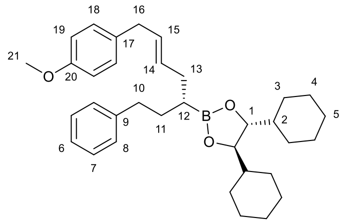

^{1}H-NMR (400 MHz, $CDCl_3$): δ = 7.28–7.24 (m, 2 H, 7-H), 7.18–7.14 (m, 3 H, 6-H, 8-H), 7.09–7.07 (m, 2 H, 18-H), 6.82–6.80 (m, 2 H, 19-H), 5.59–5.45 (m, 2 H, 14-H, 15-H), 3.25 (d, *J* = 6.0 Hz, 2 H, 16-H), 2.67–2.55 (m, 2 H, 10-H), 2.26–2.12 (m, 2 H, 13-H), 1.82 – 1.59 (m, 12 H, 3-Ha, 3-Hb, 4-Ha, 4-Hb, 5-Ha, 11-H), 1.33 – 0.97 (m, 13 H, 2 H, 3-Hc, 3-Hd, 4-Hc, 4-Hd, 12-H) ppm.

^{13}C-NMR (100 MHz, $CDCl_3$): δ = 157.8 (C-20), 143.1 (C-9), 133.1 (C-15), 131.0 (C-18), 129.9 (C-14), 129.4 (C-17), 128.4 (C-8), 128.2 (C-7), 125.5 (C-6), 113.7 (C-19), 83.4 (C-1), 55.2 (C-21), 43.1 (C-2), 38.2 (C-16), 35.5 (C-10), 34.0 (C-13), 33.0 (C-11), 28.5 (C-3a), 27.6 (C-3b), 26.5 (C-5), 26.0 (C-4a), 25.9 (C-4b) ppm.

The signal of C-12 could not be detected.

HRMS (ESI) m/z calcd for $C_{34}H_{48}BO_3$ $[M+H]^+$: 515.3691, found: 515.3672.

(*R*,*E*)-1,7-Diphenylhept-5-en-3-ol (6a)

According to GP-2, 104 mg (265 µmol) boronic ester **3**, 75.0 mg (265 µmol) Schwartz reagent and 1.32 ml anhydrous THF were used for the hydrozirconation. 9.2 mg $Pd(PPh_3)_4$ (7.94 µmol), 31.5 µl (45.2 mg, 261 µmol) benzyl bromide and additional 0.53 ml anhydrous THF were used for the Negishi coupling. The rection was worked up after 21.5 h. After column chromatography (SiO_2, CyH/EE 0-3%), the product **5a** (120 mg, 220 µmol, 83%) was obtained as a colourless oil.

110 mg (226 µmol) boronic ester **5a** was subjected to oxidation according to GP-5 using 452 µl THF, 103 µl (38.4 mg, 1.13 mmol, 33 %wt) aqueous H_2O_2 solution and a solution of 45.2 mg (1.13 mmol) NaOH in 452 µl water. The reaction was worked up after 1 h. To improve chromatographic separation, 16.2 mg (271 µmol) methylboronic acid and 1.13 ml diethyl ether were used. After column chromatography (SiO_2, CyH/EE 0-20%; C18-SiO_2, $H_2O \rightarrow$ MeCN), the product **6a** (47 mg, 177 µmol, 78%) was obtained as a colourless oil.

1 mmol scale:

According to GP-2, 505 mg (1.29 mmol) boronic ester **3**, 365 mg (1.42 mmol) Schwartz reagent and 6.43 ml anhydrous THF were used for the hydrozirconation. 44.7 mg (39.0 µmol) $Pd(PPh_3)_4$, 153 µl (220 mg, 1.29 mmol) benzyl bromide and additional 2.57 ml anhydrous THF were used for the Negishi coupling. The rection was worked up after 16 h. After column chromatography (SiO_2, CyH/EE 0-3%), the product **5a** (587 mg, 1.10 mmol, 86%) was obtained as a colourless oil.

549 mg (1.13 mmol) boronic ester **5a** was subjected to oxidation according to GP-4 using 2.26 ml THF, 516 µl (193 mg, 5.66 mmol, 33 %wt) aqueous H_2O_2 solution and a solution of 226 mg (5.66 mmol) NaOH in 2.26 ml water. The reaction was worked up after 1 h. To improve chromatographic separation, 81.0 mg (1.36 mmol) methylboronic acid and 5.66 ml diethyl ether were used. After column chromatography (SiO_2, CyH/EE 0-20%; C18-SiO_2, $H_2O \rightarrow$ MeCN), the product **6a** (248 mg, 931 µmol, 82%) was obtained as a colourless oil.

R_f (**6a**) = 0.32 (pentane/EE 8:2). $[\alpha]_D^{20}$ = +10.1 (c = 1.0, $CHCl_3$).

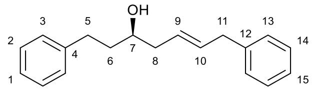

^{1}H-NMR (400 MHz, $CDCl_3$): δ = 7.30 – 7.24 (m, 4 H, 2-H, 14-H), 7.19 – 7.15 (m, 6 H, 1-H, 3-H, 13-H, 15-H), 5.74 – 5.67 (m, 1 H, 10-H), 5.54 – 5.47 (m, 1 H, 9-H), 3.67 – 3.61 (m, 1 H, 7-H), 3.36 (d, $^3J_{11,10}$ = 6.7 Hz, 2 H, 11-H), 2.83 – 2.76 (m, 1 H, 5-Ha), 2.71 – 2.63 (m, 1 H, 5-Hb), 2.32 – 2.26 (m, 1 H, 8-Ha), 2.19 – 2.12 (m, 1 H, 8-Hb), 1.80 – 1.74 (m, 2 H, 6-H), 1.63 (br s, 1 H, OH) ppm.

^{13}C-NMR (100 MHz, $CDCl_3$): δ = 142.1 (C-4), 140.4 (C-12), 133.1 (C-10), 128.4 (C-3), 128.4 (C-2, C-13), 128.4 (C-14), 127.2 (C-9), 126.0 (C-15), 125.8 (C-1), 70.2 (C-7), 40.7 (C-8), 39.1 (C-11), 38.4 (C-6), 32.0 (C-5) ppm.

HRMS (CI) m/z calcd for $C_{19}H_{21}$ $[M-OH]^+$: 249.1643, found: 249.1685.

(*R*,*E*)-7-(4-Fluorophenyl)-1-phenylhept-5-en-3-ol (6b)

According to GP-2, 114 mg (290 µmol) boronic ester **3**, 82.0 mg (318 µmol) Schwartz reagent and 1.45 ml anhydrous THF were used for the hydrozirconation. 10.0 mg (8.69 µmol) $Pd(PPh_3)_4$, 35.8 µl (54.7 mg, 290 µmol) 4-fluorobenzyl bromide and additional 0.58 ml

anhydrous THF were used for the Negishi coupling. The rection was worked up after 16 h. After column chromatography (SiO_2, CyH/EE 0-4%), the product **5b** (131 mg, 229 µmol, 79%) was obtained as a colourless oil.

113 mg (224 µmol) boronic ester **5b** was subjected to oxidation according to GP-4 using 449 µl THF, 102 µl (38.2 mg, 1.12 mmol, 33 %wt) aqueous H_2O_2 solution and a solution of 44.9 mg (1.12 mmol) NaOH in 449 µl water. The reaction was worked up after 1 h. To improve chromatographic separation, 16.1 mg (269 µmol) methylboronic acid and 1.12 ml diethyl ether were used. After column chromatography (SiO_2, CyH/EE 0-20%; C18-SiO_2, H_2O → MeCN), the product **6b** (50.1 mg, 176 µmol, 78%) was obtained as a colourless oil. R_f (**6b**) = 0.35 (pentane/EE 8:2). $[\alpha]_D^{20}$ = +7.7 (c = 1.0, $CHCl_3$).

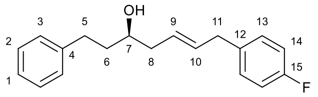

^{1}H-NMR (400 MHz, $CDCl_3$): δ = 7.31–7.27 (m, 2 H, 2-H), 7.20–7.17 (m, 3 H, 1-H, 3-H), 7.14–7.10 (m, 2 H, 13-H), 6.99–6.95 (m, 2 H, 14-H), 5.72–5.65 (m, 1 H, 9-H), 5.54–5.46 (m, 1 H, 10-H), 3.69–3.62 (m, 1 H, 7-H), 3.33 (d, *J* = 6.6 Hz, 2 H, 11-H), 2.84–2.77 (m, 1 H, 5-Ha), 2.72–2.64 (m, 1 H, 5-Hb), 2.33–2.27 (m, 1 H, 8-Ha), 2.20–2.13 (m, 1 H, 8-Hb), 1.81–1.75 (m, 2 H, 6-H), 1.55 (s, 1 H, OH) ppm.

^{13}C-NMR (100 MHz, $CDCl_3$): δ = 161.4 (d, *J* = 244.3 Hz, C-15), 142.0 (C-4), 136.0 (d, *J* = 2.9 Hz, C-12), 132.9 (C-9), 129.8 (d, *J* = 8.1 Hz, C-13), 128.4 (C-3), 128.4 (C-2), 127.4 (C-10), 125.8 (C-1), 115.2 (d, *J* = 21.3 Hz, C-14), 70.2 (C-7), 40. 7 (C-6), 38.4 (C-8), 38.2 (C-11), 32.0 (C-5).

^{19}F-NMR (400 MHz, $CDCl_3$): δ = −117.4 ppm.

HRMS (CI) m/z calcd for $C_{19}H_{19}FO$ $[M-2H]^+$: 282.1420, found: 282.1384.

(*R*,*E*)-7-(4-Bromophenyl)-1-phenylhept-5-en-3-ol (6c)

According to GP-2, 112 mg (285 µmol) boronic ester **3**, 73.5 mg (285 µmol) Schwartz reagent and 1.42 ml anhydrous THF were used for the hydrozirconation. 9.9 mg (8.55 µmol) $Pd(PPh_3)_4$, 71.2 mg (285 µmol) 1-bromo-4-(bromomethyl)benzene and additional 0.57 ml anhydrous THF were used for the Negishi coupling. The rection was worked up after 17 h. After column chromatography (SiO_2, CyH/EE 0-4%), the product **5c** (125 mg, 197 µmol, 69%) was obtained as a colourless oil.

105 mg (165 µmol) boronic ester **5c** subjected to oxidation according to GP-4 using 331 µl THF 75.0 µl (827 µmol, 33%wt) aqueous H_2O_2 solution and a solution of 33.1 mg (827 µmol) NaOH in 331 µl water. The reaction was worked up after 1 h. To improve chromatographic separation, 11.9 mg (198 µmol) methylboronic acid and 0.93 ml diethyl ether were used. After column chromatography (SiO_2, CyH/EE 0-20%; C18-SiO_2, H_2O → MeCN), the product **6c** (54.3 mg, 157 µmol, 95%) was obtained as a colourless oil. R_f (**6c**) = 0.34 (pentane/EE 8:2). $[\alpha]_D^{20}$ = +7.2 (c = 1.0, $CHCl_3$).

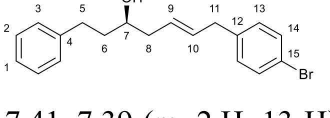

^{1}H-NMR (400 MHz, $CDCl_3$): δ = 7.41–7.39 (m, 2 H, 13-H), 7.31–7.17 (m, 2 H, 2-H), 7.21–7.18 (m, 2 H, 1-H, 3-H), 7.05–7.03 (m, 2 H, 14-H), 5.70–5.63 (m, 1 H, 10-H), 5.54–5.47 (m,

1 H, 9-H), 3.69–3.62 (m, 1 H, 7-H), 3.31 (d, J = 6.5 Hz, 11-H), 2.83–2.76 (m, 1 H, 5-Ha), 2.71–2.64 (m, 1 H, 5-Hb), 2.32–2.62 (m, 1 H, 8-Ha), 2.20–2.13 (m, 1 H, 8-Hb), 1.84–1.71 (m, 2 H, 6-H), 1.56 (s, 1 H, OH) ppm.

^{13}C-NMR (100 MHz, $CDCl_3$): δ = 142.0 (C-4), 139.4 (C-12), 132.4 (C-10), 131.5 (C-13), 130.2 (C-14), 128.4 (C-2, C-3), 127.8 (C-9), 125.9 (C-1), 119.8 (C-15), 70.2 (C-7), 40.7 (C-8), 38.4 (C-6), 38.4 (C-11), 32.0 (C-5) ppm.

HRMS (ESI) m/z calcd for $C_{19}H_{22}{}^{79}BrO$ $[M+H]^+$: 345.0849, found: 345.0835.

(*R*,*E*)-1-Phenyl-7-(*p*-tolyl)hept-5-en-3-ol (6d)

According to GP-2, 111 mg (282 µmol) boronic ester **3**, 72.7 mg (282 µmol) Schwartz reagent and 1.41 ml anhydrous THF were used for the hydrozirconation. 9.8 mg $Pd(PPh_3)_4$ (8.46 µmol), 52.2 mg (282 µmol) 4-methylbenzyl bromide and additional 0.56 ml anhydrous THF were used for the Negishi coupling. The rection was worked up after 16 h. After column chromatography (SiO_2, CyH/EE 0-3%), the product **5d** (121 mg, 220 µmol, 78%) was obtained as a colourless oil.

104 mg (208 µmol) boronic ester **5d** was subjected to oxidation according to GP-4 using 416 µl THF, 107 µl (35.3 mg, 1.04 mmol, 33 %wt) aqueous H_2O_2 solution and a solution of 41.6 mg (1.04 mmol) NaOH in 416 µl water. The reaction was worked up after 1 h. To improve chromatographic separation, 14.9 mg (249 µmol) methylboronic acid and 1.04 ml diethyl ether were used. After column chromatography (SiO_2, CyH/EE 0-20%; C18-SiO_2, H_2O → MeCN), the product **6d** (39 mg, 137 µmol, 66%) was obtained as a colourless oil. R_f (**6d**) = 0.36 (pentane/EE 8:2). $[\alpha]_D^{20}$ = +7.5 (c = 1.0, $CHCl_3$).

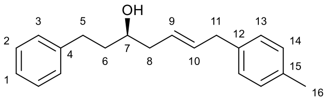

^{1}H-NMR (400 MHz, $CDCl_3$): δ = 7.31–7.27 (m, 2 H, 2-H), 7.21–7.18 (m, 3 H, 1-H, 3-H), 7.12–7.06 (m, 4 H, 13-H, 14-H), 5.75–5.67 (m, 1 H, 10-H), 5.54–5.46 (m, 1 H, 9-H), 3.69–3.62 (m, 1 H, 7-H), 3.34 (d, J = 6.7 Hz, 2 H, 11-H), 2.84–2.77 (m, 1 H, 5-Ha), 2.72–2.65 (m, 1 H, 5-Hb), 2.33 (s, 3 H, 16-H), 2.32–2.27 (m, 1 H, 8-Ha), 2.20–2.12 (m, 1 H, 8-Hb), 1.85–1.72 (m, 2 H, 6-H), 1.63 (br s, 1 H, OH) ppm.

^{13}C-NMR (100 MHz, $CDCl_3$): δ = 142.1 (C-4), 137.3 (C-12), 135.5 (C-15), 133.5 (C-10), 129.1 (C-), 128.4 (C-3), 128.4 (C-), 128.3 (C-2), 126.9 (C-9), 125.8 (C-1), 70.2 (C-7), 40.7 (C-8), 38.7 (C-6), 38.4 (C-11), 32.0 (C-5), 21.0 (C-16) ppm.

HRMS (CI) m/z calcd for $C_{20}H_{23}$ $[M-OH]^+$: 263.1800, found: 263.1843.

(*R*,*E*)-1-Phenyl-7-(*o*-tolyl)hept-5-en-3-ol (6e)

According to GP-2, 103 mg (261 µmol) boronic ester **3**, 67.4 mg (261 µmol) Schwartz reagent and 1.31 ml anhydrous THF were used for the hydrozirconation. 9.1 mg (7.84 µmol) $Pd(PPh_3)_4$, 35.0 µl (48.3 mg, 261 µmol) 1-(bromomethyl)-2-methylbenzene and additional 0.52 ml anhydrous THF were used for the Negishi coupling. The reaction was worked up after 15.5 h. After column chromatography (SiO_2, CyH/EE 0-3%), the product **5e** (116 mg, 207 µmol, 79%) was obtained as a colourless oil.

90 mg (180 µmol) boronic ester **5e** was subjected to oxidation according to GP-4 using 361 µl THF, 82.0 µl (902 µmol, 33 %wt) aqueous H_2O_2 solution and a solution of 36.1 mg (902 µmol)

NaOH in 361 µl water. The reaction was worked up after 1 h. To improve chromatographic separation, 13.0 mg (216 µmol) methylboronic acid and 0.90 ml diethyl ether were used. After column chromatography (SiO_2, CyH/EE 0-20%; C18-SiO_2, H_2O → MeCN), the product **6e** (37 mg, 132 µmol, 73%) was obtained as a colourless oil. R_f (**6e**) = 0.36 (pentane/EE 8:2). $[\alpha]_D^{20}$ = +11.0 (c = 1.0, $CHCl_3$).

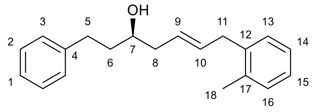

¹H-NMR (400 MHz, $CDCl_3$): δ = 7.31–7.27 (m, 2 H, 2-H, 7.21–7.13 (m, 7 H, 1-H, 3-H, 13-H, 14-H, 15-H, 16-H), 5.74–5.67 (m, 1 H, 10-H), 5.48–5.40 (m, 1 H, 9-H), 3.67–3.61 (m, 1 H, 7-H), 3.36 (d, J = 6.4 Hz, 2 H, 11-H), 2.84–2.77 (m, 1 H, 5-Ha), 2.71–2.64 (m, 1 H, 5-Hb), 2.32–2.26 (m, 4 H, 8-Ha, 18-H), 2.20–2.13 (m, 1 H, 8-Hb), 1.81–1.74 (m, 2 H, 6-H), 1.69 (br s, 1 H, OH) ppm.

¹³C-NMR (100 MHz, $CDCl_3$): δ = 142.1 (C-4), 138.5 (C-12), 136.1 (C-17), 132.2 (C-10), 130.1 (C-16), 128.9 (C-13), 128.4 (C-3), 128.3 (C-2), 127.0 (C-9), 126.2 (C-15), 126.0 (C-14), 125.7 (C-1), 70.2 (C-7), 40.7 (C-8), 38.4 (C-6), 36.6 (C-11), 32.0 (C-5), 19.3 (C-18) ppm.

HRMS (CI) m/z calcd for $C_{20}H_{23}$ $[M{-}OH]^+$: 263.1800, found: 263.1792.

(*R*,*E*)-7-(Benzo[d][1,3]dioxol-5-yl)-1-phenylhept-5-en-3-ol (6f)

According to GP-2, 104 mg (265 µmol) boronic ester **3**, 68.2 mg (271 µmol) Schwartz reagent and 1.32 ml anhydrous THF were used for the hydrozirconation. 9.2 mg (7.94 µmol) $Pd(PPh_3)_4$, 34.2 µl (265 µmol) 5-(chloromethyl)benzo[d][1.3]dioxole and additional 0.53 ml anhydrous THF were used for the Negishi coupling. The rection was worked up after 16 h. After column chromatography (SiO_2, CyH/EE 0-4%), the product **5f** (125 mg, 203 µmol, 77%) was obtained as a colourless oil.

113 mg (214 µmol) boronic ester **5f** subjected to oxidation according to GP-4 using 428 µl THF, 98.0 µl (1.07 mmol, 33%wt) aqueous H_2O_2 solution and a solution of 42.8 mg (1.07 mmol) NaOH in 428 µl water. The reaction was worked up after 1 h. To improve chromatographic separation, 15.4 mg (159 µmol) methylboronic acid and 1.07 ml diethyl ether were used. After column chromatography (SiO_2, CyH/EE 0-20%; C18-SiO_2, H_2O → MeCN), the product **6f** (48.3 mg, 156 µmol, 73%) was obtained as a colourless oil. R_f (**6f**) = 0.29 (pentane/EE 8:2). $[\alpha]_D^{20}$ = +5.6 (c = 1.0, $CHCl_3$).

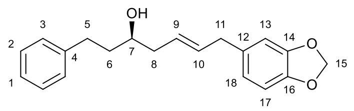

¹H-NMR (400 MHz, $CDCl_3$): δ = 7.30–7.27 (m, 2 H, 2-H), 7.21–7.17 (m, 3 H, 1-H, 3-H), 6.73 (d, J = 7.8 Hz, 1 H, 17-H), 6.66 (d, J = 1.2 Hz, 1 H, 13-H), 6.62 (dd, J_1 = 7.9 Hz, 1.2 Hz, 1 H, 18-H), 5.92 (s, 2 H, 15-H), 5.67 (dt, J = 15.2 Hz, 6.7 Hz, 1 H, 10-H), 5.49 (dt, J = 15.3 Hz, 7.3 Hz, 1 H, 9-H), 3.68–3.62 (m, 1 H, 7-H), 3.28 (d, J = 6.6 Hz, 2 H, 11-H), 2.84–2.77 (m, 1 H, 5-Ha), 2.72–2.64 (m, 1 H, 5-Hb), 2.32–2.26 (m, 1 H, 8-Ha), 2.19–2.12 (m, 1 H, 8-Hb), 1.81–1.74 (m, 2 H, 6-H), 1.58 (s, 1 H, OH) ppm.

¹³C-NMR (100 MHz, $CDCl_3$): δ = 147.6 (C-14), 145.8 (C-16), 142.1 (C-4), 134.3 (C-12), 133.3 (C-10), 128.4 (C-3), 128.4 (C-2), 127.1 (C-9), 125.8 (C-1), 121.1 (C-18), 108.9 (C-13), 108.2 (C-17), 100.8 (C-15), 70.2 (C-7), 40.7 (C-8), 38.8 (C-11), 38.4 (C-6), 32.0 (C-5) ppm.

HRMS (ESI) m/z calcd for $C_{20}H_{22}O_3$ $[M]^+$: 310.1563, found: 310.1553.

(*R*,*E*)-1-phenylnona-5,8-dien-3-ol (6g)

According to GP-2, 111 mg (282 µmol) boronic ester **3**, 72.8 mg (282 µmol) Schwartz reagent and 1.41 ml anhydrous THF were used for the hydrozirconation. 9.8 mg (8.47 µmol) $Pd(PPh_3)_4$, 24.4 µl (34.2 mg, 282 µmol) allyl bromide and additional 0.56 ml anhydrous THF were used for the Negishi coupling. The reaction was worked up after 17 h. After column chromatography (SiO_2, CyH/EE 0-3%), the product **5g** (122 mg, 239 µmol, 85%) was obtained as a colourless oil.

86.9 mg (200 µmol) boronic ester **5g** was subjected to oxidation according to GP-4 using 400 µl THF, 91.0 µl (1.00 mmol, 33 %wt) aqueous H_2O_2 solution and a solution of 40.0 mg (1.00 mmol) NaOH in 400 µl water. The reaction was worked up after 1 h. To improve chromatographic separation, 14.4 mg (240 µmol) methylboronic acid and 1.00 ml diethyl ether were used. After column chromatography (SiO_2, CyH/EE 0-20%; C18-SiO_2, H_2O → MeCN), the product **6g** (31.8 mg, 147 µmol, 74%) was obtained as a colourless oil. R_f (**6g**) = 0.41 (pentane/EE 8:2). $[\alpha]_D^{20}$ = +11.0 (c = 1.0, $CHCl_3$).

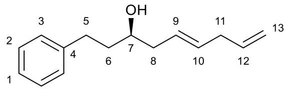

^{1}H-NMR (400 MHz, $CDCl_3$): δ = 7.31–7.27 (m, 2 H, 2-H), 7.22–7.17 (m, 3 H, 1-H, 3-H), 5.82 (ddt, *J* = 16.9 Hz, 10.3 Hz, 6.4 Hz, 1 H, 12-H), 5.61–5.54 (m, 1 H, 10-H), 5.49–5.42 (m, 1 H, 9-H), 5.06–4.99 (m, 2 H, 13-H), 3.66–3.60 (m, 1 H, 7-H), 2.85–2.77 (m, 3 H, 5-Ha, 11-H), 2.72–2.65 (m, 1 H, 5-Hb), 2.31–2.25 (m, 1 H, 8-Ha), 2.18–2.10 (m, 1 H, 8-Hb), 1.81–1.75 (m, 2 H, 6-H), 1.61 (br s, 1 H, OH) ppm.

^{13}C-NMR (100 MHz, $CDCl_3$): δ = 142.1 (C-4), 136.8 (C-12), 132.0 (C-10), 128.4 (C-3), 128.4 (C-2), 127.0 (C-9), 125.8 (C-1), 115.2 (C-13), 70.1 (C-7), 40.8 (C-8), 38.4 (C-6), 36.8 (C-11), 32.0 (C-5) ppm.

HRMS (CI) m/z calcd for $C_{15}H_{20}O$ $[M]^+$: 216.1514, found: 216.1525.

(*R*,*E*)-7-(4-(3-Hydroxyprop-1-yn-1-yl)phenyl)-1-phenylhept-5-en-3-ol (6i)

To a solution of 101 mg (291 µmol, 1.0 eq.) aryl bromide **11e** were added 5.60 mg (29 µmol, 0.1 eq.) CuI and 52.1 µl (49.0 mg, 874 µmol, 3.0 eq.) propargyl alcohol, followed by the addition of 16.8 mg (15 µmol, 0.05 eq.) $Pd(PPh_3)_4$. The reaction mixture was stirred at 80 °C for 24 h. after cooling to room temperature, sat. NH_4Cl solution was added. The phases were separated and the organic phase was extracted three times with ethyl acetate. The combined organic phases were washed with brine, dried over Na_2SO_4 and filtered. The solvent was removed under reduced pressure. The residue was purified by column chromatography (SiO_2, CyH/EE 0-40%; C18-SiO_2, H_2O → MeCN). The product **6i** (66.2 mg, 207 µmol, 71%) was obtained as a yellow oil. R_f (**6i**) = (pentane/EE). $[\alpha]_D^{20}$ = +5.6 (c = 1.0, $CHCl_3$).

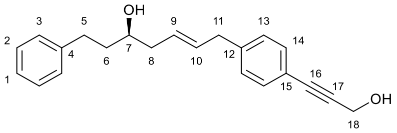

^{1}H-NMR (500 MHz, $CDCl_3$): δ = 7.37–7.35 (m, 2 H, 14-H), 7.30–7.27 (m, 2 H, 2-H), 7.20–7.18 (m, 3 H, 1-H, 3-H), 7.13–7.11 (m, 2 H, 13-H), 5.71–5.65 (m, 1 H, 10-H), 5.54–5.48 (m,

1 H, 9-H), 4.49 (s, 2 H, 18-H), 3.68–3.63 (m, 1 H, 7-H), 3.35 (d, *J* = 6.6 Hz, 11-H), 2.83–2.77 (m, 1 H, 5-Ha), 2.71–2.65 (m, 1 H, 5-Hb), 2.32–2.27 (m, 1 H, 8-Ha), 2.20–2.14 (m, 1 H, 8-Hb), 1.83–1.72 (m, 3 H, 6-H, 7-OH), 1.61 (br s, 1 H, 18-OH) ppm.

^{13}C-NMR (125 MHz, $CDCl_3$): δ = 142.0 (C-4), 141,1 (C-12), 132.3 (C-10), 131.8 (C-14), 128.5 (C-3), 128.4 (C-2), 128.4 (C-13),127.8 (C-9), 125.8 (C-1), 120.2 (C-15), 86.8 (C-17), 85.7 (C-16), 70.2 (C-7), 51.7 (C-18), 40.7 (C-8), 38.9 (C-11), 38.4 (C-6), 32.0 (C-5) ppm.

HRMS (ESI) m/z calcd for $C_{22}H_{23}O_2$ $[M-H]^-$: 319.1704, found: 319.1698.

(*R*,*E*)-1,6-Diphenylhex-5-en-3-ol (8a)

According to GP-3, 104 mg (265 µmol) boronic ester **3**, 68.4 mg (265 µmol) Schwartz reagent and 0.35 ml anhydrous THF were used for the hydrozirconation. 20.2 mg (18.0 µmol) $Pd(PPh_3)_4$, 59.2 µl (108 mg, 531 µmol) iodobenzene and 47.0 mg (345 µmol) $ZnCl_2$ in 0.18 ml anhydrous THF were used for the Negishi coupling. The reaction was worked up after 17.5 h. After column chromatography (SiO_2, CyH/EE 0-3%), the product **7a** (105 mg, 207 µmol, 78%) was obtained as a colourless oil.

91 mg (167 µmol) boronic ester **7a** was subjected to oxidation according to GP-4 using 385 µl THF, 88.0 µl (99.0 mg, 962 µmol, 33 %wt) aqueous H_2O_2 solution and a solution of 38.5 mg (962 µmol) NaOH in 385 µl water. The reaction was worked up after 1 h. To improve chromatographic separation, 13.8 mg (231 µmol) methylboronic acid and 0.96 ml diethyl ether were used. After column chromatography (SiO_2, CyH/EE 0-20%; C18-SiO_2, H_2O → MeCN), the product **8a** (39 mg, 153 µmol, 80%) was obtained as a colourless oil.

1 mmol scale:

According to GP-3, 501 mg (1.28 mmol) boronic ester **3**, 330 mg (1.28 mmol) Schwartz reagent and 1.70 ml anhydrous THF were used for the hydrozirconation. 97.0 mg (84.0 µmol) $Pd(PPh_3)_4$, 285 µl (521 mg, 2.56 mmol) iodobenzene and 226 mg (1.66 mmol) $ZnCl_2$ in 0.85 ml anhydrous THF were used for the Negishi coupling. The reaction was worked up after 16 h. After column chromatography (SiO_2, CyH/EE 0-3%), the product **7a** (464 mg, 917 µmol, 72%) was obtained as a colourless oil.

459 mg (976 µmol) boronic ester **7a** was subjected to oxidation according to GP-4 using 1.95 ml THF, 445 µl (503 mg, 4.88 mmol, 33 %wt) aqueous H_2O_2 solution and a solution of 195 mg (4.88 mmol) NaOH in 1.95 ml water. The reaction was worked up after 1 h. To improve chromatographic separation, 70.1 mg (1.71 mmol) methylboronic acid and 4.88 ml diethyl ether were used. After column chromatography (SiO_2, CyH/EE 0-20%; C18-SiO_2, H_2O → MeCN), the product **8a** (179 mg, 711 µmol, 73%) was obtained as a colourless oil.

R_f (**8a**) = 0.30 (pentane/EE 8:2). $[\alpha]_D^{20}$ = +7.9 (c = 1.0, $CHCl_3$).

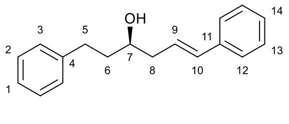

^{1}H-NMR (400 MHz, $CDCl_3$): δ = 7.37–7.35 (m, 2 H, 12-H), 7.32–7.27 (m, 4 H, 2-H, 13-H), 7.24–7.18 (m, 4 H, 1-H, 3-H, 14-H), 6.49 (d, *J* = 15.9 Hz, 10-H), 6.22 (dt, *J* = 15.7 Hz, 7.6 Hz, 1 H, 9-H), 3.80–3.72 (m, 1 H, 7-H), 2.88–2.81 (m, 1 H, 5-Ha), 2.76–2.68 (m, 1 H, 5-Hb), 2.51–2.44 (m, 1 H, 8-Ha), 2.39–2.31 (m, 1 H, 8-Hb), 1.87–1.81 (m, 2 H, 6-H), 1.64 (d, *J* = 4.3 Hz, 1 H, OH) ppm.

^{13}**C-NMR** (100 MHz, $CDCl_3$): δ = 142.0 (C-4), 137.1 (C-11), 133.4 (C-10), 128.5 (C-13), 128.5 (C-3), 128.4 (C-2), 127.3 (C-14), 126.1 (C-12), 126.0 (C-9), 125.8 (C-1), 70.4 (C-7), 41.3 (C-8), 38.5 (C-6), 32.1 (C-5) ppm.

HRMS (CI) m/z calcd for $C_{18}H_{21}O$ $[M+H]^+$: 253.1592, found: 253.1589.

(*R*,*E*)-1-Phenyl-6-(*p*-tolyl)hex-5-en-3-ol (8b)

According to GP-3, 107 mg (272 µmol) boronic ester **3**, 73.6 mg (285 µmol) Schwartz reagent and 0.36 ml anhydrous THF were used for the hydrozirconation. 20.7 mg (17.0 µmol) $Pd(PPh_3)_4$, 118 mg (543 µmol) 1-iodo-4-methylbenzene and 48.1 mg (353 µmol) $ZnCl_2$ in 0.18 ml anhydrous THF were used for the Negishi coupling. The reaction was worked up after 16 h. After column chromatography (SiO_2, CyH/EE 0-3%), the product **7b** (100 mg, 165 µmol, 61%) was obtained as a colourless oil.

81 mg (167 µmol) boronic ester **7b** was subjected to oxidation according to GP-4 using 334 µl THF, 76.0 µl (834 µmol, 33%wt) aqueous H_2O_2 solution and a solution of 33.4 mg (834 mmol) NaOH in 334 µl water. The reaction was worked up after 1 h. To improve chromatographic separation, 12.0 mg (185 µmol) methylboronic acid and 0.84 ml diethyl ether were used. After column chromatography (SiO_2, CyH/EE 0-20%; C18-SiO_2, H_2O → MeCN), the product **8b** (41 mg, 153 µmol, 92%) was obtained as a colourless oil. R_f (**8b**) = 0.39 (pentane/EE 8:2). $[\alpha]_D^{20}$ = +6.2 (c = 1.0, $CHCl_3$).

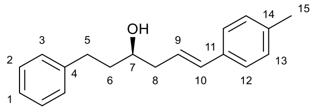

^{1}**H-NMR** (400 MHz, $CDCl_3$): δ = 7.30–7.16 (m, 7 H, 1-H, 2-H, 3-H, 12-H), 7.11–7.09 (m, 2 H, 13-H), 6.44 (d, *J* = 15.9 Hz, 1 H, 10-H), 6.15 (dt, *J* = 15.8 Hz, *J* = 7.6 Hz, 1 H, 9-H), 3.77–3.70 (m, 1 H, 7-H), 2.86–2.79 (m, 1 H, 5-Ha), 2.74–2.67 (m, 1 H, 5-Hb), 2.48–2.42 (m, 1 H, 8-Ha), 2.36–2.28 (m, 4 H, 8-Hb, 15-H), 1.85–1.79 (m, 2 H, 6-H), 1.64 (d, *J* = 4.2 Hz, 1 H, OH) ppm.

^{13}**C-NMR** (100 MHz, $CDCl_3$): δ = 142.0 (C-4), 137.1 (C-14), 134.3 (C-11), 133.3 (C-10), 129.2 (C-13), 128.5 (C-3), 128.4 (C-2), 126.0 (C-12), 125.8 (C-1), 124.9 (C-9), 70.4 (C-7), 41.3 (C-8), 38.5 (C-6), 32.1 (C-5), 21.1 (C-15) ppm.

HRMS (CI) m/z calcd for $C_{19}H_{23}O$ $[M+H]^+$: 267.1749, found: 267.1742.

(*R*,*E*)-6-(4-Methoxyphenyl)-1-phenylhex-5-en-3-ol (8c)

According to GP-3, 103 mg (262 µmol) boronic ester **3**, 67.4 mg (275 µmol) Schwartz reagent and 0.35 ml anhydrous THF were used for the hydrozirconation. 20.0 mg (17.0 µmol) $Pd(PPh_3)_4$, 123 mg (524 µmol) 1-iodo-4-methoxybenzene and 46.4 mg (341 µmol) $ZnCl_2$ in 0.18 ml anhydrous THF were used for the Negishi coupling. The reaction was worked up after 16 h. After column chromatography (SiO_2, CyH/EE 0-3%), the product **7c** (107 mg, 190 µmol, 73%) was obtained as a colourless oil.

77 mg (154 µmol) boronic ester **7c** was subjected to oxidation according to GP-4 using 308 µl THF, 70.2 µl (769 µmol, 33%wt) aqueous H_2O_2 solution and a solution of 30.8 mg (769 µmol) NaOH in 308 µl water. The reaction was worked up after 1 h. To improve chromatographic separation, 11.1 mg (185 µmol) methylboronic acid and 0.77 ml diethyl ether were used. After column chromatography (SiO_2, CyH/EE 0-20%; C18-SiO_2, H_2O → MeCN), the product **8c**

(32 mg, 112 µmol, 73%) was obtained as a colourless oil. R_f (**8c**) = 0.25 (pentane/EE 8:2). $[\alpha]_D^{20}$ = +7.6 (c = 1.0, $CHCl_3$).

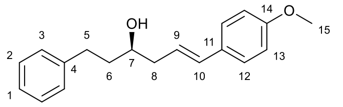

^{1}H-NMR (400 MHz, $CDCl_3$): δ = 7.31–7.27 (m, 4 H, 2-H, 12-H), 7.23–7.17 (m, 3 H, 1-H, 3-H), 6.86–6.84 (m, 2 H, 13-H), 6.43 (d, *J* = 15.8 Hz, 1 H, 10-H), 6.07 (dt, *J* = 15.9 Hz, 7.3 Hz, 1 H, 9-H), 3.81 (s, 3 H, 15-H), 3.76–3.71 (m, 1 H, 7-H), 2.88–2.80 (m, 1 H, 5-Ha), 2.75–2.68 (m, 1 H, 5-Hb), 2.48–2.42 (m, 1 H, 8-Ha), 2.35–2.28 (m, 1 H, 8-Hb), 1.86–1.80 (m, 2 H, 6-H), 1.66 (d, *J* = 3.5 Hz, 1 H, OH) ppm.

^{13}C-NMR (100 MHz, $CDCl_3$): δ = 159.0 (C-14), 142.0 (C-4), 132.8 (C-10), 130.0 (C-11), 128.4 (C-3), 128.4 (C-2), 127.2 (C-12), 125.8 (C-1), 123.7 (C-9), 113.9 (C-13), 70.4 (C-7), 55.3 (C-15), 41.3 (C-8), 38.5 (C-6), 32.1 (C-5) ppm.

HRMS (CI) m/z calcd for $C_{19}H_{23}O$ $[M+H]^+$: 267.1749, found: 267.1761.

(*R*,*E*)-6-(4-Fluorophenyl)-1-phenylhex-5-en-3-ol (8d)

According to GP-3, 106 mg (270 µmol) boronic ester **3**, 69.6 mg (270 µmol) Schwartz reagent and 0.36 ml anhydrous THF were used for the hydrozirconation. 20.6 mg (18.0 µmol) $Pd(PPh_3)_4$, 62.2 µl (540 µmol) 1-fluoro-4-iodobenzene, 47.8 mg (351 µmol) $ZnCl_2$ and additional 0.18 ml anhydrous THF were used for the Negishi coupling. The reaction was worked up after 15 h. After column chromatography (SiO_2, CyH/EE 0-3%), the product **7d** (105.9 mg, 186 µmol, 69%) was obtained as a colourless oil.

95.3 mg (195 µmol) boronic ester **7d** was subjected to oxidation according to GP-4 using 390 µl THF, 89.0 µl (975 µmol, 33%wt) aqueous H_2O_2 solution and a solution of 39.0 mg (975 µmol) NaOH in 390 µl water. The reaction was worked up after 1 h. To improve chromatographic separation, 14.0 mg (234 µmol) methylboronic acid and 0.98 ml diethyl ether were used. After column chromatography (SiO_2, CyH/EE 0-20%; C18-SiO_2, H_2O → MeCN), the product **8d** (33.8 mg, 125 µmol, 64%) was obtained as a colourless oil. R_f (**8d**) = 0.29 (pentane/EE 8:2). $[\alpha]_D^{20}$ = +8.7 (c = 1.0, $CHCl_3$).

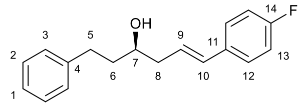

^{1}H-NMR (400 MHz, $CDCl_3$): δ = 7.33–7.18 (m, 7 H, 1-H, 2-H, 3-H, 12-H), 7.01–6.99 (m, 2 H, 13-H), 6.44 (d, *J* = 15.8 Hz, 1 H, 10-H), 6.13 (dt, *J* = 15.5 Hz, *J* = 7.5 Hz, 1 H, 9-H), 3.78–3.72 (m, 1 H, 7-H), 2.88–2.80 (m, 1 H, 5-Ha), 2.75–2.68 (m, 1 H, 5-Hb), 2.49–2.43 (m, 1 H, 8-Ha), 2.37–2.30 (m, 1 H, 8-Hb), 1.87–1.81 (m, 2 H, 6-H), 1.62 (br s, 1 H, OH) ppm.

^{13}C-NMR (100 MHz, $CDCl_3$): δ = 162.3 (*J* = 246.5 Hz, C-14), 141.9 (C-4), 133.3 (*J* = 3.7 Hz, C-11), 132.1 (C-10), 128.4 (C-3), 127.5 (*J* = 8.1 Hz, C-12), 125.8 (C-9), 125.8 (C-1), 115.3 (*J* = 22.0 Hz, C-13), 70.4 (C-7), 41.2 (C-8), 38.6 (C-6), 32.1 (C-5) ppm.

^{19}F-NMR (400 MHz, $CDCl_3$): δ = −114.8 ppm.

HRMS (CI) m/z calcd for $C_{18}H_{19}FO$ $[M]^+$: 270.1420, found: 270.1375.

Ethyl (*R*,*E*)-2-(4-hydroxy-6-phenylhex-1-en-1-yl)benzoate (8e)

According to GP-3, 113 mg (288 µmol) boronic ester **3**, 74.2 mg (288 µmol) Schwartz reagent and 0.38 ml anhydrous THF were used for the hydrozirconation. 21.9 mg (18.0 µmol) $Pd(PPh_3)_4$, 159 mg (575 µmol) ethyl 2-iodobenzoate and 51.0 mg (374 µmol) $ZnCl_2$ in 0.19 ml anhydrous THF were used for the Negishi coupling. The reaction was worked up after 17.5 h. After column chromatography (SiO_2, CyH/EE 0-3%), the product **7e** (148 mg, 254 µmol, 88%) was obtained as a colourless oil.

To a solution of 132 mg (243 µmol, 1.0 eq.) boronic ester **7e** in 486 µl THF were added 111 µmol (41.3 mg, 1.22 mmol, 33%wt, 5.0 eq.) aqueous H_2O_2 solution and a solution of 129 mg (1.22 mmol, 5.0 eq.) Na_2CO_3 in 486 µl water at 0 °C. The reaction mixture was warmed to room temperature and stirred for 1h. The reaction mixture was then washed with brine and the aqueous phase was extracted three times with Et_2O. The combined organic phases were dried over Na_2SO_4 and filtered. The solvent was removed under reduced pressure. To improve separation of **8e** and DICHED, the crude product was diluted in 1.22 ml Et_2O and 17.5 mg (292 µmol, 1.2 eq.) methylboronic acid was added with an excess of $MgSO_4$ and the reaction mixture was stirred for 2 h at room temperature. The reaction mixture was filtered and the solvent was removed under reduced pressure. The crude product was purified by column chromatography (SiO_2, CyH/EE 0-20%; C18-SiO_2, H_2O → MeCN). The product **8e** (45.7 mg, 141 µmol, 58%) was obtained as a colourless oil. R_f (**8e**) = 0.29 (pentane/EE 8:2). $[\alpha]_D^{20}$ = +23.5 (c = 1.0, $CHCl_3$).

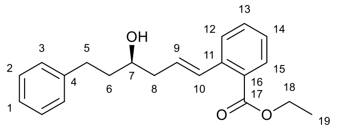

^{1}H-NMR (400 MHz, $CDCl_3$): δ = 7.89 (d, J = 8.1 Hz, 1 H, 15-H), 7.49–7.43 (m, 2 H, 13-H, 14-H), 7.32–7.27 (m, 3 H, 2-H, 12-H), 7.24–7.15 (m, 4 H, 1-H, 3-H, 10-H), 6.03 (ddd, J = 15.5 Hz, 8.3 Hz, 6.8 Hz, 1 H, 9-H), 4.36 (q, J = 7.1 Hz, 2 H, 18-H), 3.79–3.73 (m, 1 H, 7-H), 2.90–2.83 (m, 1 H, 5-Ha), 2.77–2.70 (m, 1 H, 5-Hb), 2.52–2.46 (m, 1 H, 8-Ha), 2.39–2.31 (m, 1 H, 8-Hb), 2.30 (s, 1 H, OH), 1.88–1.82 (m, 2 H, 6-H), 1.38 (t, J = 7.1 Hz, 3 H, 19-H) ppm.

^{13}C-NMR (100 MHz, $CDCl_3$): δ = 167.4 (C-17), 142.2 (C-4), 139.6 (C-11), 133.0 (C-10), 132.0 (C-13), 130.2 (C-15), 128.8 (C-16), 128.5 (C-3, C-9), 128.3 (C-2), 127.6 (C-14), 127.0 (C-12), 125.8 (C-1), 70.1 (C-18), 61.1 (C-7), 41.1 (C-8), 38.6 (C-6), 32.1 (C-5), 14.3 (C-19) ppm.

HRMS (CI) m/z calcd for $C_{21}H_{25}O_3$ $[M+H]^+$: 325.1804, found: 325.1807.

(*R*,*E*)-4-(4-Hydroxy-6-phenylhex-1-en-1-yl)benzonitrile (8f)

According to GP-3, 157 mg (399 µmol) boronic ester **3**, 103 mg (399 µmol) Schwartz reagent and 0.53 ml anhydrous THF were used for the hydrozirconation. 30.5 mg (26.0 µmol) $Pd(PPh_3)_4$, 183 mg (799 µmol) 4-iodobenzonitrile and 70.8 mg (519 µmol) $ZnCl_2$ in 0.27 ml anhydrous THF were used for the Negishi coupling. The reaction was worked up after 15 h. After column chromatography (SiO_2, CyH/EE 0-3%), the product **7f** (142 mg, 286 µmol, 72%) was obtained as a colourless oil.

To a solution of 117 mg (237 µmol, 1.0 eq.) boronic ester **7f** in 473 µl THF were added 40.1 mg (260 µmol, 1.1 eq.) $NaBO_3 \cdot 4\ H_2O$ and a solution of 10.4 mg (260 µmol, 1.1 eq.) NaOH in 473 µl water at 0 °C. The reaction mixture was warmed to room temperature and stirred for 1h. The reaction mixture was then washed with brine and the aqueous phase was extracted three times with Et_2O. The combined organic phases were dried over Na_2SO_4 and filtered. The solvent

was removed under reduced pressure. To improve separation of **7f** and DICHED, the crude product was dissolved in 1.19 ml Et_2O and 17.0 mg (284 µmol, 1.2 eq.) methylboronic acid was added with an excess of $MgSO_4$ and the reaction mixture was stirred for 2 h at room temperature. The reaction mixture was filtered and the solvent was removed under reduced pressure. The crude product was purified by column chromatography (SiO_2, CyH/EE 0-20%; C18-SiO_2, H_2O → MeCN). The product **7f** (58.1 mg, 209 µmol, 88%) was obtained as a colourless oil. R_f (**7f**) = 0.19 (pentane/EE 8:2). $[\alpha]_D^{20}$ = +8.4 (c = 1.0, $CHCl_3$).

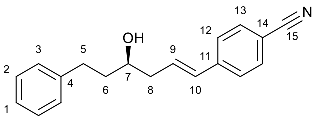

¹H-NMR (400 MHz, $CDCl_3$): δ = 7.58 (d, J = 8.3 Hz, 2 H, 13-H), 7.42 (d, J = 8.2 Hz, 12-H), 7.31–7.18 (m, 2 H, 2-H), 7.22–7.18 (m, 3 H, 1-H, 3-H), 6.48 (d, J = 16.0 Hz, 1 H, 10-H), 6.39 (dt, J = 15.8 Hz, 7.0 Hz, 1 H, 9-H), 3.83–3.76 (m, 1 H, 7-H), 2.87–2.80 (m, 1 H, 5-Ha), 2.75–2.68 (m, 1 H, 5-Hb), 2.53–2.47 (m, 1 H, 8-Ha), 2.43–2.36 (m, 1 H, 8-Hb), 1.87–1.81 (m, 2 H, 6-H), 1.58 (s, 1 H, OH) ppm.

¹³C-NMR (100 MHz, $CDCl_3$): δ = 141.7 (C-4), 141.6 (C-11), 132.4 (C-13), 131.5 (C-10), 130.6 (C-9), 128.5 (C-3), 128.4 (C-2), 126.6 (C-12), 126.0 (C-1), 119.0 (C-15), 110.4 (C-14), 70.4 (C-7), 41.3 (C-8), 38.7 (C-6), 32.0 (C-5) ppm.

HRMS (CI) m/z calcd for $C_{19}H_{20}NO$ $[M+H]^+$: 278.1545, found: 278.1577.

(*R*,5*E*,7*E*)-1,8-diphenylocta-5,7-dien-3-ol (8g)

According to GP-3, 101 mg (295 µmol) boronic ester **3**, 66.2 mg (257 µmol) Schwartz reagent and 0.34 ml anhydrous THF were used for the hydrozirconation. 19.6 mg (17.0 µmol) $Pd(PPh_3)_4$, 69.5 µl (118 mg, 514 µmol) (*E*)-(2-iodovinyl)benzene[9] and 45.5 mg (334 µmol) $ZnCl_2$ in 0.17 ml anhydrous THF were used for the Negishi coupling. The reaction was worked up after 18 h. After column chromatography (SiO_2, CyH/EE 0-3%), the product **7g** (100 mg, 185 µmol, 72%) was obtained as a yellow oil.

80.8 mg (150 µmol) boronic esters **7g** was subjected to oxidation according to GP-4 using 325 µl THF, 74.2 µl (27.7 mg, 814 µmol, 33%wt) aqueous H_2O_2 solution and a solution of 32.5 mg (814 µmol) NaOH in 325 µl water. The reaction was worked up after 1 h. To improve chromatographic separation, 11.7 mg (195 µmol) methylboronic acid and 0.82 ml diethyl ether were used. After column chromatography (SiO_2, CyH/EE 0-20%; C18-SiO_2, H_2O → MeCN), the product **8g** (28.1 mg, 101 µmol, 67%) was obtained as a colourless oil. R_f (**8g**) = 0.31 (pentane/EE 8:2). $[\alpha]_D^{20}$ = +1.4 (c = 1.0, $CHCl_3$).

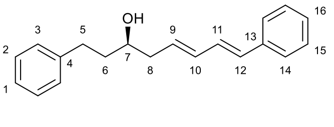

¹H-NMR (400 MHz, $CDCl_3$): δ = 7.39–7.37 (m, 2 H, 14-H), 7.35–7.27 (m, 4 H, 2-H, 15-H), 7.23–7.18 (m, 4 H, 1-H, 3-H, 16-H), 6.76 (dd, J = 15.6 Hz, 10.4 Hz, 1 H, 11-H), 6.49 (d, J = 15.6 Hz, 1 H, 12-H), 6.30 (dd, J = 15.1 Hz, 10.5 Hz, 1 H, 10-H), 5.81 (dt, J = 15.2 Hz, 7.6 Hz, 1 H, 9-H), 3.75–3.68 (m, 1 H, 7-H), 2.86–2.79 (m, 1 H, 5-Ha), 2.74–2.67 (m, 1 H, 5-Hb), 2.44–2.38 (m, 1 H, 8-Ha), 2.33–2.25 (m, 1 H, 8-Hb), 1.84–1.78 (m, 2 H, 6-H), 1.59 (d, J = 4.3 Hz, 1 H, OH) ppm.

^{13}C-NMR (100 MHz, $CDCl_3$): δ = 142.0 (C-4), 137.3 (C-13), 134.0 (C-12), 131.3 (C-11), 130.4 (C-9), 128.7 (C-10), 128.6 (C-15), 128.4 (C-3), 128.4 (C-2), 127.4 (C-16), 126.2 (C-14), 125.9 (C-1), 70.4 (C-7), 41.1 (C-8), 38.5 (C-6), 32.1 (C-5) ppm.

HRMS (ESI) m/z calcd for $C_{20}H_{23}O$ $[M+H]^+$: 279.1743, found: 279.1731.

Ethyl (*R*,2*Z*,4*E*)-7-hydroxy-9-phenylnona-2,4-dienoate (8h)

107 mg (273 µmol, 1.0 eq.) boronic ester **3**, 77.0 mg (273 µmol, 1.1 eq.) Schwartz reagent were suspended in 0.82 ml anhydrous THF at room temperature in the absence of light. The reaction mixture was stirred for 10 min until it became clear. To a solution of 15.8 mg (14.0 µmol, 5 mol-%) $Pd(PPh_3)_4$ in 0.55 ml anhydrous THF was added a solution of 41.9 µl (74.0 mg, 327 µmol, 1.2 eq.) ethyl (*Z*)-3-iodoacrylate in 0.55 ml anhydrous THF. The mixture was stirred for 5 min at room temperature before the solution of the freshly prepared vinylzirconium reagent is added. A solution of 74.3 mg (287 µmol, 2.0 eq.) $ZnCl_2$ in 0.55 ml anhydrous THF was added and the reaction mixture was stirred for 16 h at room temperature. Saturated NH_4Cl solution is added and the phases were separated. The aqueous phase was extracted three times with diethyl ether. The combined organic phases were washed with saturated $NaHCO_3$ solution, dried over Na_2SO_4 and filtered. The solvent was removed under reduced pressure and the residue was purified by column chromatography (SiO_2, CyH/EE 0-3%). The product **7h** (116 mg, 185 µmol, 68%) was obtained as a colourless oil.

To a solution of 99,6 mg (202 µmol, 1.0 eq.) boronic esters **7h** in 404 µl THF were added 34.2 mg (222 µmol, 1.1 eq.) $NaBO_3 \cdot 4\ H_2O$ and 404 µl Sørensen phosphate buffer pH 8 at 0 °C. The reaction mixture was warmed to room temperature and stirred for 5 h. The reaction mixture was then washed with brine and the aqueous phase was extracted three times with Et_2O. The combined organic phases were dried over Na_2SO_4 and filtered. The solvent was removed under reduced pressure. To improve separation of **7h** and DICHED, the crude product was dissolved in 1.01 ml Et_2O and 14.5 mg (243 µmol, 1.2 eq.) methylboronic acid was added with an excess of $MgSO_4$ and the reaction mixture was stirred for 2 h at room temperature. The reaction mixture was filtered and the solvent was removed under reduced pressure. The crude product was purified by column chromatography (SiO_2, CyH/EE 0-20%; C18-SiO_2, H_2O → MeCN). The product **8h** (38.8 mg, 141 µmol, 89%) was obtained as a colourless oil. R_f (**8h**) = 0.31 (pentane/EE 8:2). $[\alpha]_D^{20}$ = +1.7 (c = 1.0, $CHCl_3$).

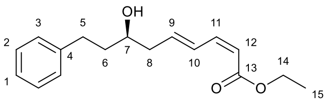

^{1}H-NMR (400 MHz, $CDCl_3$): δ = 7.49–7.42 (m, 1 H, 10-H), 7.31–7.27 (m, 2 H, 2-H), 7.21–7.17 (m, 3 H, 1-H, 3-H), 6.55 (dd, *J* = 11.3 Hz, 1 H, 11-H), 6.07 (dt, *J* = 15.3 Hz, 7.5 Hz, 1 H, 9-H), 5.61 (d, *J* = 11.4 Hz, 1 H, 12-H), 4.18 (q, *J* = 7.1 Hz, 2 H, 14-H), 3.79–3.72 (m, 1 H, 7-H), 2.85–2.77 (m, 1 H, 5-Ha), 2.73–2.65 (m, 1 H, 5-Hb), 2.48–2.33 (m, 2 H, 8-H), 1.83–1.77 (m, 2 H, 6-H), 1.52 (d, J = 4.5 Hz, 1 H, OH), 1.29 (t, *J* = 7.1 Hz, 3 H, 15-H) ppm.

^{13}C-NMR (100 MHz, $CDCl_3$): δ = 166.4 (C-13), 144.4 (C-11), 141.8 (C-4), 140.3 (C-9), 129.6 (C-10), 128.4 (C-3), 128.4 (C-2), 125.9 (C-1), 116.7 (C-12), 70.4 (C-7), 60.0 (C-14), 41.1 (C-8), 38.7 (C-6), 32.0 (C-5), 14.3 (C-15) ppm.

HRMS (CI) m/z calcd for $C_{17}H_{21}O_3$ $[M-H]^+$: 273.1491, found: 273.1490.

(4*R*,5*R*)-4,5-Dicyclohexyl-2-((R)-1,6-diphenylhex-5-yn-3-yl)-1,3,2-dioxaborolane (9a)

According to GP-5, 150 mg (382 µmol) boronic ester **3**, 26.8 mg (38.0 µmol) $PdCl_2(PPh_3)_2$, 14.5 mg (76.0 µmol) CuI, 213 µl (389 mg, 1.91 mmol) iodobenzene and 2.54 ml NEt_3 were used. The reaction mixture was worked up after 16 h. After column chromatography (SiO_2, CyH/EE 0-3%), the product **9a** (178 mg, 379 µmol, 99%) was obtained as a yellow oil. R_f (**9a**) = 0.30 (pentane/EE 97:3). $[\alpha]_D^{20}$ = +34.0 (c = 1.0, $CHCl_3$).

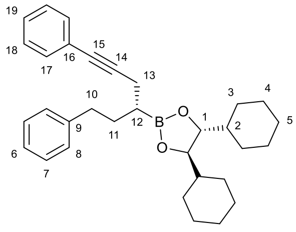

^{1}H-NMR (400 MHz, $CDCl_3$): δ = 7.37–7.35 (m, 2 H, 17-H), 7.29–7.16 (m, 8 H, 6-H, 7-H, 8-H, 18-H, 19-H), 3.90–3.87 (m, 2 H, 1-H), 2.72–2.68 (m, 2 H, 10-H), 2.57 (d, *J* = 6.9 Hz, 2 H, 13-H), 1.94–1.84 (m, 2 H, 11-H), 1.81–1.61 (m, 10 H, 3-Ha, 3-Hb, 4-Ha, 4-Hb, 5-Ha), 1.44–0.95 (m, 13 H, 2-H, 3-Hc, 3-Hd, 4-Hc, 4-Hd, 5-Hb, 12-H) ppm.

^{13}C-NMR (100 MHz, $CDCl_3$): δ = 142.8 (C-9), 131.6 (C-17), 128.4 (C-8), 128.3 (C-7), 128.1 (C-19), 127.3 (C-18), 125.6 (C-6), 124.1 (C-16), 90.1 (C-14), 83.5 (C-1), 81.0 (C-15), 43.0 (C-2), 35.1 (C-10), 32.7 (C-11), 28.4 (C-3a), 27.4 (C-3b), 26.4 (C-5), 26.0 (C-4a), 25.9 (C-4b), 20.9 (C-13) ppm.

The signal of C-12 could not be detected.

HRMS (CI) m/z calcd for $C_{32}H_{41}BO_2$ $[M]^+$: 468.3200, found: 468.3208.

(4*R*,5*R*)-4,5-dicyclohexyl-2-((*R*)-1-phenyl-6-(*p*-tolyl)hex-5-yn-3-yl)-1,3,2-dioxaborolane (9b)

According to GP-5, 149 mg (379 µmol) boronic ester **3**, 26.6 mg (36.0 µmol) $PdCl_2(PPh_3)_2$, 14.4 mg (76.0 µmol) CuI, 413 mg (1.90 mmol) 1-iodo-4-methylbenzene and 2.53 ml NEt_3 were used. The reaction mixture was worked up after 16 h. After column chromatography (SiO_2, CyH/EE 0-3%), the product **9b** (183 mg, 379 µmol, 100%) was obtained as a yellow oil. R_f (**9b**) = 0.29 (pentane/EE 97:3). $[\alpha]_D^{20}$ = +33.6 (c = 1.0, $CHCl_3$).

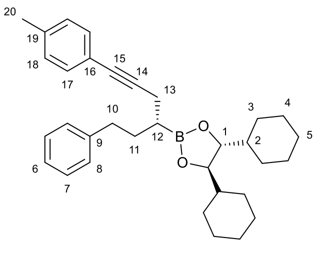

^{1}H-NMR (400 MHz, $CDCl_3$): δ = 7.29–7.25 (m, 4 H, 7-H, 17-H), 7.22–7.15 (m, 3 H, 6-H, 8-H), 7.08–7.06 (m, 2 H, 18-H), 3.90–3.87 (m, 2 H, 1-H), 2.74–2.65 (m, 2 H, 10-H), 2.56 (d, *J* = 6.7 Hz, 2 H, 13-H), 2.32 (s, 3 H, 20-H), 1.97–1.86 (m, 2 H, 11-H), 1.84–1.61 (m, 10 H, 3-Ha, 3-Hb, 4-Ha, 4-Hb, 5-Ha), 1.43–0.95 (m, 13 H, 2-H, 3-Hc, 3-Hd, 4-Hc, 4-Hd, 5-Hb, 12-H) ppm.

^{13}C-NMR (100 MHz, $CDCl_3$): δ = 142.8 (C-9), 137.3 (C-19), 131.4 (C-17), 128.8 (C-18), 128.5 (C-8), 128.3 (C-7), 125.6 (C-6), 121.1 (C-16), 89.2 (C-14), 83.5 (C-1), 81.0 (C-15), 43.0 (C-2),

35.1 (C-10), 32.6 (C-11), 28.4 (C-3a), 27.4 (C-3b), 26.4 (C-5), 26.0 (C-4a), 25.9 (C-4b), 21.4 (C-20), 20.9 (C-13) ppm.

The signal of C-12 could not be detected.

HRMS (CI) m/z calcd for $C_{33}H_{42}BO_2$ [M−H]$^+$: 481.3278, found: 481.3230.

(4*R*,5*R*)-4,5-Dicyclohexyl-2-((*R*)-6-(4-methoxyphenyl)-1-phenylhex-5-yn-3-yl)-1,3,2-dioxaborolane (9c)

According to GP-5, 160 mg (406 µmol) boronic ester **3**, 28.5 mg (41.0 µmol) $PdCl_2(PPh_3)_2$, 15.5 mg (81.0 µmol) CuI, 476 mg (2.03 mmol) 1-iodo-4-methoxybenzene and 2.71 ml NEt_3 were used. The reaction mixture was worked up after 18 h. After column chromatography (SiO_2, CyH/EE 0-3%), the product **9c** (202 mg, 405 µmol, 100%) was obtained as a yellow oil. R_f (**9c**) = 0.26 (pentane/EE 97:3). $[\alpha]_D^{20}$ = +33.3 (c = 1.0, $CHCl_3$).

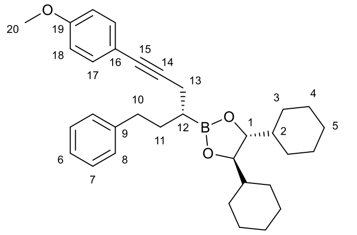

^{1}H-NMR (400 MHz, $CDCl_3$): δ = 7.31–7.24 (m, 4 H, 7-H, 17-H), 7.22–7.16 (m, 3 H, 6-H, 8-H), 6.81–6.79 (m, 2 H, 18-H), 3.90–3.87 (m, 2 H, 1-H), 3.80 (s, 3 H, 20-H), 2.73–2.64 (m, 2 H, 10-H), 2.55 (d, *J* = 6.9 Hz, 13-H), 1.97–1.84 (m, 2 H, 11-H), 1.81–1.61 (m, 12 H, 3-Ha, 3-Hb, 4-Ha, 4-Hb, 5-Ha, 11-H), 1.43–0.97 (m, 13 H, 2-H, 3-Hc, 3-Hd, 4-Hc, 4-Hd, 5-Hb, 12-H) ppm.

^{13}C-NMR (100 MHz, $CDCl_3$): δ = 158.9 (C-19), 142.8 (C-9), 132.9 (C-17), 128.5 (C-8), 128.3 (C-7), 125.6 (C-6), 116.4 (C-16), 113.7 (C-18), 88.4 (C-14), 83.5 (C-1), 80.7 (C-15), 55.2 (C-20), 43.0 (C-2), 35.1 (C-10), 32.7 (C-11), 28.3 (C-3a), 27.4 (C-3b), 26.4 (C-5), 26.0 (C-4a), 25.9 (C-4b), 20.9 (C-13) ppm.

The signal of C-12 could not be detected.

HRMS (CI) m/z calcd for $C_{33}H_{43}BO_3$ [M]$^+$: 498.3305, found: 498.3277.

(4*R*,5*R*)-4,5-Dicyclohexyl-2-((*R*)-6-(4-fluorophenyl)-1-phenylhex-5-yn-3-yl)-1,3,2-dioxaborolane (9d)

According to GP-5, 101 mg (257 µmol) boronic ester **3**, 18.0 mg (26.0 µmol) $PdCl_2(PPh_3)_2$, 9.8 mg (51.0 µmol) CuI, 148 µl (285 mg, 1.28 mmol) 1-fluoro-4-iodobenzene and 1.71 ml NEt_3 were used. The reaction mixture was worked up after 18 h. After column chromatography (SiO_2, CyH/EE 0-3%), the product **9d** (122 mg, 251 µmol, 98%) was obtained as a yellow oil. R_f (**9d**) = 0.33 (pentane/EE 97:3). $[\alpha]_D^{20}$ = +32.4 (c = 1.0, $CHCl_3$).

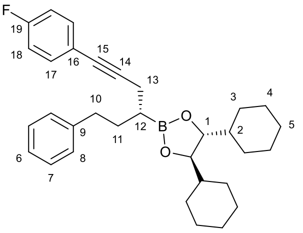

^{1}H-NMR (400 MHz, $CDCl_3$): δ = 7.35–7.24 (m, 4 H, 17-H, 7-H), 7.21–7.16 (m, 3 H, 6-H, 8-H), 6.98–6.93 (m, 2 H, 18-H), 3.90–3.87 (m, 2 H, 1-H), 2.74–2.66 (m, 2 H, 10-H), 2.55 (d, *J* = 6.9 Hz, 2 H, 13-H), 1.97–1.84 (m, 2 H, 11-H), 1.83–1.61 (m, 12 H, 3-Ha, 3-Hb, 4-Ha, 4-Hb, 5-Ha, 11-H), 1.44–0.97 (m, 13 H, 2-H, 3-Hc, 3-Hd, 4-Hc, 4-Hd, 5-Hb, 12-H) ppm.

^{13}C-NMR (100 MHz, $CDCl_3$): δ = 161.9 (*J* = 248.0 Hz,C-19), 142.7 (C-9), 133.3 (*J* = 8.1 Hz, C-17), 128.4 (C-8), 128.3 (C-7), 125.6 (C-6), 120.2 (*J* = 2.9 Hz, C-16), 115.2 (*J* = 22.0 Hz, C-18), 89.7 (C-14), 83.5 (C-1), 79.9 (C-15), 43.0 (C-2), 35.1 (C-10), 32.7 (C-11), 28.3 (C-3a), 27.4 (C-3b), 26.4 (C-5), 26.0 (C-4a), 25.9 (C-4b), 20.8 (C-13) ppm.

The signal of C-12 could not be detected.

^{19}F-NMR (400 MHz, $CDCl_3$): δ = −112.5 ppm.

HRMS (CI) m/z calcd for $C_{32}H_{40}BFO_2$ $[M]^+$: 486.3105, found: 486.3109.

(4*R*,5*R*)-4,5-dicyclohexyl-2-((*R*)-6-(2-methoxyphenyl)-1-phenylhex-5-yn-3-yl)-1,3,2-dioxaborolane (9e)

According to GP-5, 159 mg (406 µmol) boronic ester **3**, 28.5 mg (41.0 µmol) $PdCl_2(PPh_3)_2$, 15.5 mg (81.0 µmol) CuI, 476 mg (2.03 mmol) 1-iodo-2-methoxybenzene and 2.71 ml NEt_3 were used. The reaction mixture was worked up after 16 h. After column chromatography (SiO_2, CyH/EE 0-2%), the product **9e** (202 mg, 405 µmol, 100%) was obtained as a yellow oil.

<u>1 mmol scale</u>

According to GP-5, 492 mg (1.25 mmol) boronic ester **3**, 88.0 mg 125 µmol) $PdCl_2(PPh_3)_2$, 47.8 mg (251 µmol) CuI, 522, 1.47 g (6.27 mmol) 1-iodo-2-methoxybenzene and 8.36 ml NEt_3 were used. The reaction mixture was worked up after 16 h. After column chromatography (SiO_2, CyH/EE 0-2%), the product **9e** (546 mg, 1.09 mmol, 87%) was obtained as a yellow oil.

R_f (**9e**) = 0.36 (pentane/EE 97:3). $[\alpha]_D^{20}$ = +37.8 (c = 1.0, $CHCl_3$).

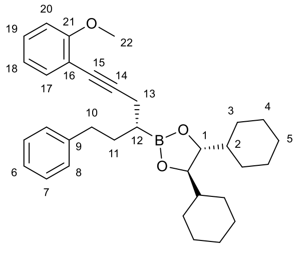

^{1}H-NMR (400 MHz, $CDCl_3$): δ = 7.34 (dd, *J* = 7.6 Hz, 1.5 Hz, 1 H, 17-H), 7.29–7.15 (m, 6 H, 6-H, 7-H, 8-H, 19-H), 6.88–6.82 (m, 2 H, 18-H, 20-H), 3.89–3.86 (m, 2 H, 1-H), 3.83 (s, 3 H, 22-H), 2.74–2.57 (m, 4 H, 10-H, 13-H), 1.97–1.91 (m, 2 H, 11-H), 1.80–1.60 (m, 10 H, 3-Ha, 3-Hb, 4-Ha, 4-Hb, 5-Ha), 1.46–0.93 (m, 13 H, 2-H, 3-Hc, 3-Hd, 4-Hc, 4-Hd, 5-Hb, 12-H) ppm.

^{13}C-NMR (100 MHz, $CDCl_3$): δ = 159.9 (C-21), 142.9 (C-9), 133.6 (C-17), 128.7 (C-19), 128.5 (C-8), 128.2 (C-7), 125.6 (C-6), 120.2 (C-18), 113.3 (C-16), 110.4 (C-20), 94.3 (C-14), 83.5 (C-1), 77.1 (C-15), 55.7 (C-22), 43.0 (C-2), 35.1 (C-10), 32.6 (C-11), 28.4 (C-3a), 27.4 (C-3b), 26.4 (C-5), 26.0 (C-4a), 25.9 (C-4b), 21.1 (C-13) ppm.

The signal of C-12 could not be detected.

HRMS (CI) m/z calcd for $C_{33}H_{43}BO_3$ $[M]^+$: 498.3305, found: 498.3317.

(4*R*,5*R*)-4,5-Dicyclohexyl-2-((*R*)-6-(4-nitrophenyl)-1-phenylhex-5-yn-3-yl)-1,3,2-dioxaborolane (9f)

According to GP-5, 98.7 mg (252 µmol) boronic ester **3**, 17.7 mg (25.0 µmol) $PdCl_2(PPh_3)_2$, 9.6 mg (50.0 µmol) CuI, 313 mg (1.26 mmol) 1-iodo-4-nitrobenzene and 1.68 ml NEt_3 were used. The reaction mixture was worked up after 18 h. After column chromatography (SiO_2, CyH/EE 0-2%), the product **9f** (119 mg, 232 µmol, 92%) was obtained as a yellow oil. R_f (**9f**) = 0.24 (pentane/EE 97:3). $[\alpha]_D^{20}$ = +24.2 (c = 1.0, $CHCl_3$).

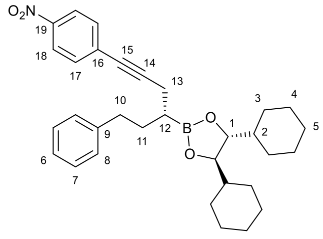

^{1}H-NMR (400 MHz, $CDCl_3$): δ = 8.15–8.13 (m, 2 H, 18-H), 7.49–7.47 (m, 2 H, 17-H), 7.30–7.26 (m, 2 H, 7-H), 7.21–7.17 (m, 3 H, 6-H, 8-H), 3.91–3.88 (m, 2 H, 1-H), 2.73–2.68 (m, 2 H, 10-H), 2.61 (d, *J* = 6.7 Hz, 2 H, 13-H), 1.95–1.85 (m, 2 H, 11-H), 1.80–1.60 (m, 10 H, 3-Ha, 3-Hb, 4-Ha, 4-Hb, 5-Ha), 1.37–0.96 (m, 13 H, 2-H, 3-Hc, 3-Hd, 4-Hc, 4-Hd, 5-Hb, 12-H) ppm.

^{13}C-NMR (100 MHz, $CDCl_3$): δ = 146.5 (C-19), 142.5 (C-9), 132.2 (C-17), 128.4 (C-16), 128.4 (C-8), 128.3 (C-7), 125.8 (C-6), 123.4 (C-18), 96.7 (C-14), 83.6 (C-1), 79.7 (C-15), 43.0 (C-2), 35.0 (C-10), 32.7 (C-11), 28.3 (C-3a), 27.4 (C-3b), 26.4 (C-5), 26.0 (C-4a), 25.9 (C-4b), 21.0 (C-13) ppm.

The signal of C-12 could not be detected.

HRMS (CI) m/z calcd for $C_{32}H_{41}BNO_4$ $[M+H]^+$: 514.3129, found: 514.3097.

(4*R*,5*R*)-4,5-Dicyclohexyl-2-((*R*,*Z*)-6-(2-methoxyphenyl)-1-phenylhex-5-en-3-yl)-1,3,2-dioxaborolane (10e)

86.3 mg (173 µmol, 1.0 eq.) boronic ester **9e** and 89 mg (346 µmol, 2.0 eq.) Schwartz reagent were suspended in 1.08 ml anhydrous DCM (0.16 M) in the absence of light. The reaction mixture was stirred for 3 h at room temperature until the white precipitate was completely dissolved. Saturated NH_4Cl solution was added and the phases were separated. The aqueous phase was extracted three times with pentane. The combined organic phases were dried over Na_2SO_4 and filtered. The solvent was removed under reduced pressure. The crude product was purified by column chromatography (SiO_2, CyH/EE 0-3%). The product **10e** (33.7 mg, 67.0 µmol, 39%, 62% brsm) was obtained as a colourless oil. R_f (**10e**) = 0.26 (pentane/EE 8:2). $[\alpha]_D^{20}$ = +31.4 (c = 1.0, $CHCl_3$).

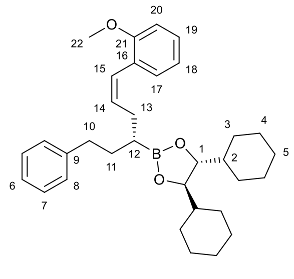

^{1}H-NMR (400 MHz, $CDCl_3$): δ = 7.28–7.19 (m, 4 H, 7-H, 17-H, 19-H), 7.16–7.12 (m, 3 H, 6-H, 8-H), 6.91 (app t, *J* = 7.4 Hz, 1 H, 18-H), 6.85 (d, *J* = 8.2 Hz, 1 H, 20-H), 6.52 (d, *J* = 11.5 Hz,

1 H, 15-H), 5.74 (dt, *J* = 11.6 Hz, 7.2 Hz, 1 H, 14-H), 3.87–3.84 (m, 2 H, 1-H), 3.80 (s, 3 H, 22-H), 2.61–2.50 (m, 2 H, 10-H), 2.49–2.35 (m, 2 H, 13-H), 1.82–1.59 (m, 12 H, 3-Ha, 3-Hb, 4-Ha, 4-Hb, 5-Ha, 11-H), 1.32–0.94 (m, 13 H, 2-H, 3-Hc, 3-Hd, 4-Hc, 4-Hd, 5-Hb, 12-H) ppm.

^{13}C-NMR (100 MHz, $CDCl_3$): δ = 157.0 (C-21), 143.1 (C-9), 132.5 (C-14), 130.1 (C-19), 128.4 (C-8), 128.2 (C-7), 127.9 (C-17), 126.5 (C-16), 125.5 (C-6), 124.5 (C-15), 120.0 (C-18), 110.3 (C-20), 83.4 (C-1), 55.3 (C-22), 43.1 (C-2), 35.5 (C-10), 33.1 (C-11), 29.9 (C-13), 28.5 (C-3a), 27.5 (C-3b), 26.4 (C-5), 26.0 (C-4a), 25.9 (C-4b) ppm.

The signal of C-12 could not be detected.

HRMS (CI) m/z calcd for $C_{33}H_{45}BO_3$ $[M]^+$: 500.3462, found: 500.3455.

(*R*,*Z*)-1,6-diphenylhex-5-en-3-ol (11a)

171 mg (364 µmol, 1.0 eq.) Boronic ester **9a** and 188 mg (728 µmol, 2.0 eq.) Schwartz reagent were suspended in 2.28 ml anhydrous DCM (0.16 M) in the absence of light. The reaction mixture was stirred for 2.5 h at room temperature until the white precipitate was completely dissolved. Saturated NH_4Cl solution was added and the phases were separated. The aqueous phase was extracted three times with pentane. The combined organic phases were dried over Na_2SO_4 and filtered. The solvent was removed under reduced pressure. The crude product was purified by column chromatography (SiO_2, CyH/EE 0-3%). The product **10a** (152 mg, 322 µmol, 88%) was obtained as a colourless oil.

67.3 mg (143 µmol) Boronic ester **10a** was subjected to oxidation according to GP-4 using 286 µl THF, 65.2 µl (24.3 mg, 715 µmol, 33%wt) aqueous H_2O_2 solution and a solution of 28.6 mg (715 µmol) NaOH in 286 µl water. The reaction was worked up after 1 h. To improve chromatographic separation, 10.3 mg (172 µmol) methylboronic acid and 0.72 ml diethyl ether were used. After column chromatography (SiO_2, CyH/EE 0-20%; C18-SiO_2, H_2O → MeCN), the product **11a** (28.1 mg, 111 µmol, 78%) was obtained as a colourless oil. R_f (**11a**) = 0.34 (pentane/EE 8:2). $[\alpha]_D^{20}$ = −0.6 (c = 1.0, $CHCl_3$).

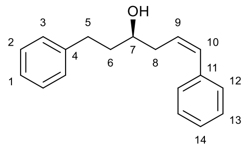

^{1}H-NMR (400 MHz, $CDCl_3$): δ = 7.35–7.18 (m, 10 H, 1-H, 2-H, 3-H, 12-H, 13-H, 14-H), 6.60 (d, *J* = 11.7 Hz, 1 H, 10-H), 5.73 (dt, *J* = 11.6 Hz, 7.4 Hz, 1 H, 9-H), 3.80–3.73 (m, 1 H, 7-H), 2.89–2.48 (m, 4 H, 5-H, 8-H), 1.87–1.76 (m, 2 H, 6-H), 1.54 (d, *J* = 4.2 Hz, 1 H, OH) ppm.

^{13}C-NMR (100 MHz, $CDCl_3$): δ = 141.9 (C-4), 137.1 (C-11), 131.8 (C-10), 128.7 (C-13), 128.4 (C-2, C-3), 128.2 (C-9), 128.0 (C-12), 126.8 (C-14), 125.8 (C-1), 71.0 (C-7), 38.6 (C-6), 36.5 (C-8), 32.0 (C-5) ppm.

HRMS (CI) m/z calcd for $C_{18}H_{19}$ $[M-OH]^+$: 235.1487, found: 235.1508.

(*R*,*Z*)-1-Phenyl-6-(*p*-tolyl)hex-5-en-3-ol (11b)

166 mg (345 µmol, 1.0 eq.) Boronic ester **9b** and 178 mg (689 µmol, 2.0 eq.) Schwartz reagent were suspended in 2.15 ml anhydrous DCM (0.16 M) in the absence of light. The reaction mixture was stirred for 2.5 h at room temperature until the white precipitate was completely dissolved. Saturated NH_4Cl solution was added and the phases were separated. The aqueous

phase was extracted three times with pentane. The combined organic phases were dried over Na_2SO_4 and filtered. The solvent was removed under reduced pressure. The crude product was purified by column chromatography (CyH/EE 0-3%). The product **10b** (141 mg, 290 µmol, 84%) was obtained as a colourless oil.

126 mg (260 µmol) Boronic esters **10b** was subjected to oxidation according to GP-4 using 521 µl THF, 119 µl (44.3 mg, 1.30 mmol, 33%wt) aqueous H_2O_2 solution and a solution of 52.1 mg (1.30 mmol) NaOH in 521 µl water. The reaction was worked up after 1 h. To improve chromatographic separation, 18.7 mg (313 µmol) methylboronic acid and 1.30 ml diethyl ether were used. After column chromatography (SiO_2, CyH/EE 0-20%; C18-SiO_2, H_2O → MeCN), the product **11b** (53.0 mg, 199 µmol, 76%) was obtained as a colourless oil. R_f (**11b**) = 0.31 (pentane/EE 8:2). $[\alpha]_D^{20} = -4.4$ (c = 1.0, $CHCl_3$).

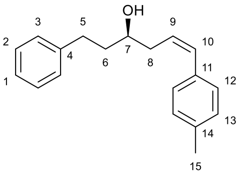

^{1}H-NMR (400 MHz, $CDCl_3$): δ = 7.31–7.24 (m, 2 H, 2-H), 7.22–7.09 (m, 7 H, 1-H, 3-H, 12-H, 13-H), 6.56 (d, *J* = 11.7 Hz, 1 H, 10-H), 5.68 (dt, *J* = 11.7 Hz, 7.3 Hz, 1 H, 9-H), 3.79–3.73 (m, 1 H, 7-H), 2.87–2.73 (m, 1 H, 5-Ha), 2.72–2.63 (m, 1 H, 5-Hb), 2.60–2.50 (m, 2 H, 8-H), 2.34 (s, 3 H, 15-H), 1.85–1.78 (m, 2 H, 6-H), 1.60 (br s, 1 H, OH) ppm.

^{13}C-NMR (100 MHz, $CDCl_3$): δ = 142.0 (C-4), 136.5 (C-11), 134.3 (C-14), 131.6 (C-10), 128.9 (C-12), 128.7 (C-13), 128.4 (C-3), 128.4 (C-2), 127.3 (C-9), 125.8 (C-1), 71.1 (C-7), 38.6 (C-6), 36.5 (C-8), 32.0 (C-5), 21.2 (C-15) ppm.

HRMS (CI) m/z calcd for $C_{19}H_{22}O$ $[M]^+$: 266.1671, found: 266.1664.

(*R*,*Z*)-6-(4-methoxyphenyl)-1-phenylhex-5-en-3-ol (11c)

187 mg (376 µmol, 1.0 eq.) Boronic ester **9c** and 194 mg (751 µmol, 2.0 eq.) Schwartz reagent were suspended in 2.35 ml anhydrous DCM (0.16 M) in the absence of light. The reaction mixture was stirred for 2.5 h at room temperature until the white precipitate was completely dissolved. Saturated NH_4Cl solution was added and the phases were separated. The aqueous phase was extracted three times with pentane. The combined organic phases were dried over Na_2SO_4 and filtered. The solvent was removed under reduced pressure. The crude product was purified by column chromatography (SiO_2, CyH/EE 0-3%). The product **10c** (133 mg, 265 µmol, 71%) was obtained as a colourless oil.

127 mg (254 µmol) Boronic ester **10c** was subjected to oxidation according to GP-4 using 507 µl THF, 116 µl (43.2 mg, 1.27 mmol, 33%wt) aqueous H_2O_2 solution and a solution of 50.7 mg (1.27 mmol) NaOH in 507 µl water. The reaction was worked up after 1 h. To improve chromatographic separation, 18.2 mg (304 µmol) methylboronic acid and 1.27 ml diethyl ether were used. After column chromatography (SiO_2, CyH/EE 0-20%; C18-SiO_2, H_2O → MeCN), the product **11c** (64.5 mg, 228 µmol, 90%) was obtained as a colourless oil.

<u>1 mmol scale</u>

527 mg (1.06 mmol, 1.0 eq.) Boronic ester **9c** and 545 mg (2.12 mmol, 2.0 eq.) Schwartz reagent were suspended in 6.61 ml anhydrous DCM (0.16 M) in the absence of light. The reaction mixture was stirred for 2.5 h at room temperature until the white precipitate was

completely dissolved. Saturated NH_4Cl solution was added and the phases were separated. The aqueous phase was extracted three times with pentane. The combined organic phases were dried over Na_2SO_4 and filtered. The solvent was removed under reduced pressure. The crude product was purified by column chromatography (SiO_2, CyH/EE 0-3%). The product **10c** (441 mg, 880 µmol, 83%) was obtained as a colourless oil.

426 mg (850 µmol) Boronic ester **10c** was subjected to oxidation according to GP-4 using 1.70 ml THF, 388 µl (438 mg, 4.25 mmol, 33%wt) aqueous H_2O_2 solution and a solution of 170 mg (4.25 mmol) NaOH in 1.70 ml water. The reaction was worked up after 1 h. To improve chromatographic separation, 61.1 mg (1.02 mmol) methylboronic acid and 4.25 ml diethyl ether were used. After column chromatography (SiO_2, CyH/EE 0-20%; C18-SiO_2, H_2O → MeCN), the product **11c** (202 mg, 716 µmol, 84%) was obtained as a colourless oil.

R_f (**11c**) = 0.23 (pentane/EE 8:2). $[\alpha]_D^{20} = -2.4$ (c = 1.0, $CHCl_3$).

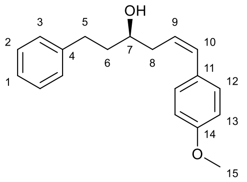

¹H-NMR (400 MHz, $CDCl_3$): δ = 7.35–7.17 (m, 7 H, 1-H, 2-H, 3-H, 12-H), 6.88–6.86 (m, 2 H, 13-H), 6.53 (d, J = 11.6 Hz, 1 H, 10-H), 5.62 (dt, J = 11.7 Hz, 7.3 Hz, 1 H, 9-H), 3.81 (s, 3 H, 15-H), 3.79–3.74 (m, 1 H, 7-H), 2.83–2.76 (m, 1 H, 5-Ha), 2.71–2.63 (m, 1 H, 5-Hb), 2.60–2.48 (m, 1 H, 8-H), 1.85–1.79 (m, 2 H, 6-H), 1.55 (d, J = 4.5 Hz, 1 H, OH) ppm.

¹³C-NMR (100 MHz, $CDCl_3$): δ = 158.4 (C-14), 142.0 (C-4), 131.2 (C-10), 130.0 (C-12), 129.8 (C-11), 128.5 (C-3), 128.4 (C-2), 128.4 (C-12), 126.4 (C-9), 125.8 (C-1), 113.6 (C-13), 71.1 (C-7), 55.2 (C-15), 38.6 (C-6), 36.5 (C-8), 32.0 (C-5) ppm.

HRMS (CI) m/z calcd for $C_{19}H_{21}O$ $[M-H]^+$: 265.1592, found: 265.1600.

(*R*,*Z*)-6-(4-Fluorophenyl)-1-phenylhex-5-en-3-ol (11d)

174 mg (358 µmol, 1.0 eq.) Boronic ester **9d** and 185 mg (716 µmol, 2.0 eq.) Schwartz reagent were suspended in 2.24 ml anhydrous DCM (0.16 M) in the absence of light. The reaction mixture was stirred for 2.5 h at room temperature until the white precipitate was completely dissolved. Saturated NH_4Cl solution was added and the phases were separated. The aqueous phase was extracted three times with pentane. The combined organic phases were dried over Na_2SO_4 and filtered. The solvent was removed under reduced pressure. The crude product was purified by column chromatography (SiO_2, CyH/EE 0-3%). The product **10d** (153 mg, 313 µmol, 87%) was obtained as a colourless oil.

143 mg (294 µmol) Boronic esters **10d** was subjected to oxidation according to GP-4 using 587 µl THF, 134 µl (49.9 mg, 1.47 mmol, 33%wt) aqueous H_2O_2 solution and a solution of 58.7 mg (1.47 mmol) NaOH in 587 µl water. The reaction was worked up after 1 h. To improve chromatographic separation, 21.1 mg (352 µmol) methylboronic acid and 1.47 ml diethyl ether were used. After column chromatography (SiO_2, CyH/EE 0-20%; C18-SiO_2, H_2O → MeCN), the product **11d** (60.5 mg, 224 µmol, 76%) was obtained as a colourless oil. R_f (**11d**) = 0.29 (pentane/EE 8:2). $[\alpha]_D^{20} = -8.8$ (c = 1.0, $CHCl_3$).

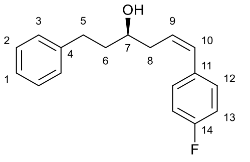

^{1}H-NMR (400 MHz, $CDCl_3$): δ = 7.40–7.18 (m, 7 H, 1-H, 2-H, 3-H, -H), 7.04–6.99 (m, 2 H, -H), 6.54 (d, *J* = 11.6 Hz, 10-H), 5.71 (dt, *J* = 11.7 Hz, 7.3 Hz, 1H, 9-H), 3.80–3.73 (m, 1 H, 7-H), 2.83–2.74 (m, 1 H, 5-Ha), 2.71–2.63 (m, 1 H, 5-Hb), 2.56–2.45 (m, 2 H, 8-H), 1.84–1.78 (m, 2 H, 6-H), 1.54 (d, *J* = 4.2 Hz, 1-H, OH) ppm.

^{13}C-NMR (100 MHz, $CDCl_3$): δ = 161.6 (*J* = 246.5 Hz, C-14), 141.8 (C-4), 133.5 (*J* = 8.1 Hz, C-12), 130.6 (C-10), 130.3 (*J* = 8.1 Hz, C-11), 128.4 (C-3), 128.4 (C-2), 128.0 (C-9), 125.9 (C-1), 115.1 (*J* = 21.3 Hz, C-13), 71.0 (C-7), 38.6 (C-6), 36.3 (C-8), 32.0 (C-5) ppm.

^{19}F-NMR (400 MHz, $CDCl_3$): δ = −115.20 ppm.

HRMS (CI) m/z calcd for $C_{18}H_{19}FO$ $[M]^+$: 270.1420, found: 270.1419.

(4*R*,5*R*)-4,5-Dicyclohexyl-2-((*R*,*E*)-5-iodo-1-phenyl-6-(*p*-tolyl)hex-5-en-3-yl)-1,3,2-dioxaborolane and (4*R*,5*R*)-4,5-dicyclohexyl-2-((*R*,*E*)-6-iodo-1-phenyl-6-(*p*-tolyl)hex-5-en-3-yl)-1,3,2-dioxaborolane (12b & 13b)

132 mg (274 µmol, 1.0 eq.) Boronic ester **9b** and 141 mg (584 µmol, 2.0 eq.) Schwartz reagent were suspended in 2.42 ml anhydrous DCM in the absence of light. The reaction mixture was stirred for 2.5 h at room temperature until the white precipitate was completely dissolved. Then, a solution of 123 mg (548 µmol, 2.0 eq.) *N*-iodosuccinimide in 1.10 ml anhydrous THF was added dropwise and the reaction mixture was stirred for 30 min at room temperature. Pentane and a 1:1 (v/v) mixture of saturated $Na_2S_2O_3$ and $NaHCO_3$ solution were added. The phases were separated and the aqueous phase was extracted twice with pentane. The combined organic phases were dried over Na_2SO_4, filtered and concentrated under reduced pressure. The residue was purified by column chromatography (cyclohexane/ethyl acetate 0-3%). The products **12b** (89.0 mg, 145 µmol, 53%) and **13b** (55.2 mg, 90.0 µmol, 33%) were obtained as a colourless oil and as a mixture. R_f (**12b**) = R_f (**13b**) = 0.36 (pentane/EE 97:3). $[\alpha]_D^{20}$ = +32.8 (c = 1.0, $CHCl_3$).

Major regioisomer **12b**:

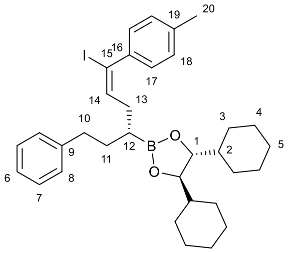

^{1}H-NMR (400 MHz, $CDCl_3$): δ = 7.29–7.21 (m, 2 H, 7-H), 7.20–7.05 (m, 7 H, 6-H, 8-H, 17-H, 18-H), 6.47 (t, *J* = 7.4 Hz, 1 H, 14-H), 3.86–3.85 (m, 2 H, 1-H), 2.55–2.49 (m, 2 H, 10-H), 2.33 (s, 3 H, 20-H), 2.17–2.13 (m, 2 H, 13-H), 1.81–1.50 (m, 12 H, 3-Ha, 3-Hb, 4-Ha, 4-Hb, 5-Ha, 11-H), 1.33–0.96 (m, 13 H, 2-H, 3-Hc, 3-Hd, 4-Hc, 4-Hd, 5-Hb, 12-H) ppm.

^{13}C-NMR (100 MHz, $CDCl_3$): δ = 143.0 (C-14), 142.6 (C-9), 141.4 (C-18), 137.7 (C-19), 134.9 (C-16), 129.0 (C-17), 128.7 (C-8), 128.2 (C-7), 125.6 (C-6), 95.3 (C-15), 83.5 (C-1), 43.1 (C-

2), 35.3 (C-10), 33.6 (C-13), 32.0 (C-11), 28.5 (C-3a), 27.6 (C-3b), 26.5 (C-5), 26.0 (C-4a), 25.9 (C-4b), 21.2 (C-20) ppm.

The signal of C-12 could not be detected.

Minor regioisomer **13b** (selected signals):

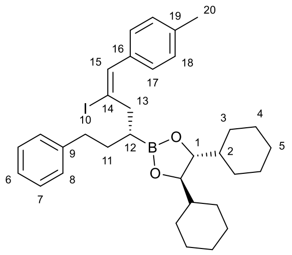

^{1}H-NMR (400 MHz, $CDCl_3$): δ = 7.20–7.05 (m, 8 H, 6-H, 8-H, 15-H, 17-H, 18-H), 3.82–3.81 (m, 2 H, 1-H), 2.71–2.67 (m, 2 H, 10-H), 2.32 (s, 3 H, 20-H) ppm.

^{13}C-NMR (100 MHz, $CDCl_3$): δ = 142.8 (C-15), 138.9 (C-19), 136.9 (C-16), 110.2 (C-14), 83.5 (C-1) ppm.

The signal of C-12 could not be detected.

HRMS (ESI) m/z calcd for $C_{33}H_{45}BO_2$ $[M+H]^+$: 611.2552, found: 611.2521.

Copies of NMR spectra

((*R*)-4-((4*R*,5*R*)-4,5-Dicyclohexyl-1,3,2-dioxaborolan-2-yl)-6-phenylhex-1-yn-1-yl)trimethylsilane (2)

^{1}H-NMR (400 MHz, $CDCl_3$):

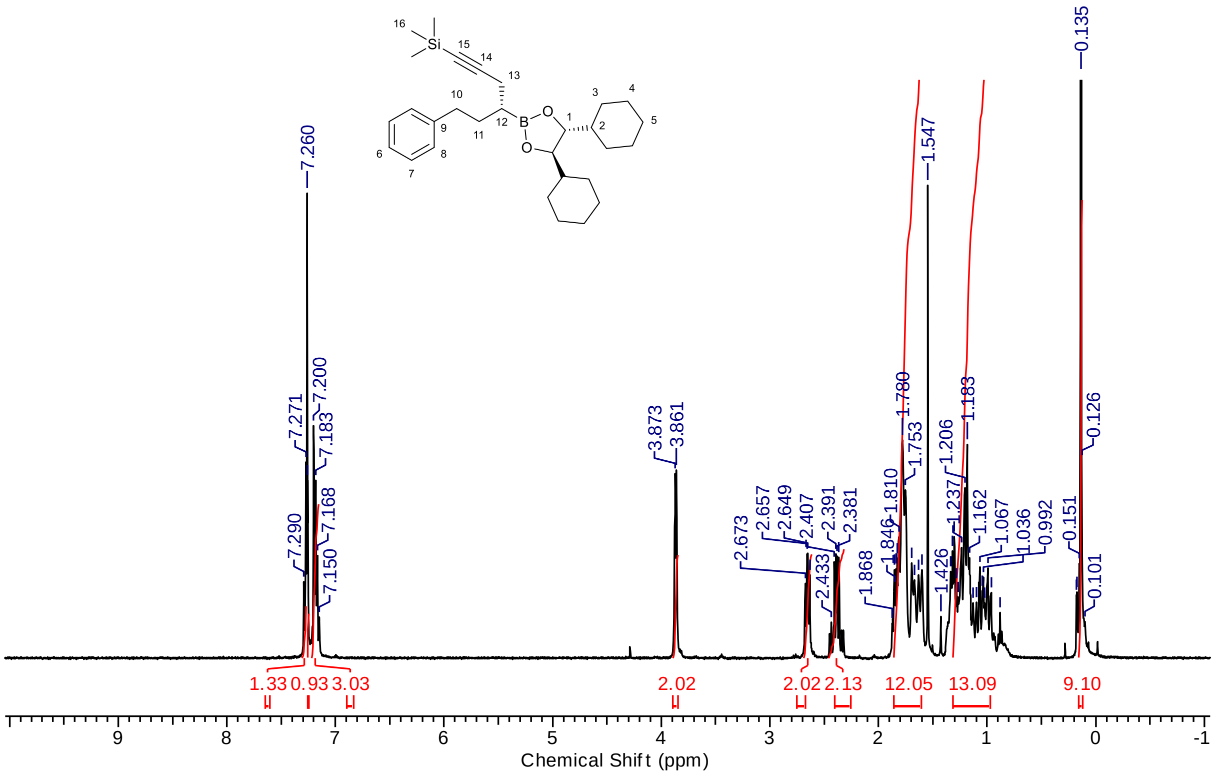

^{13}C-NMR (100 MHz, $CDCl_3$):

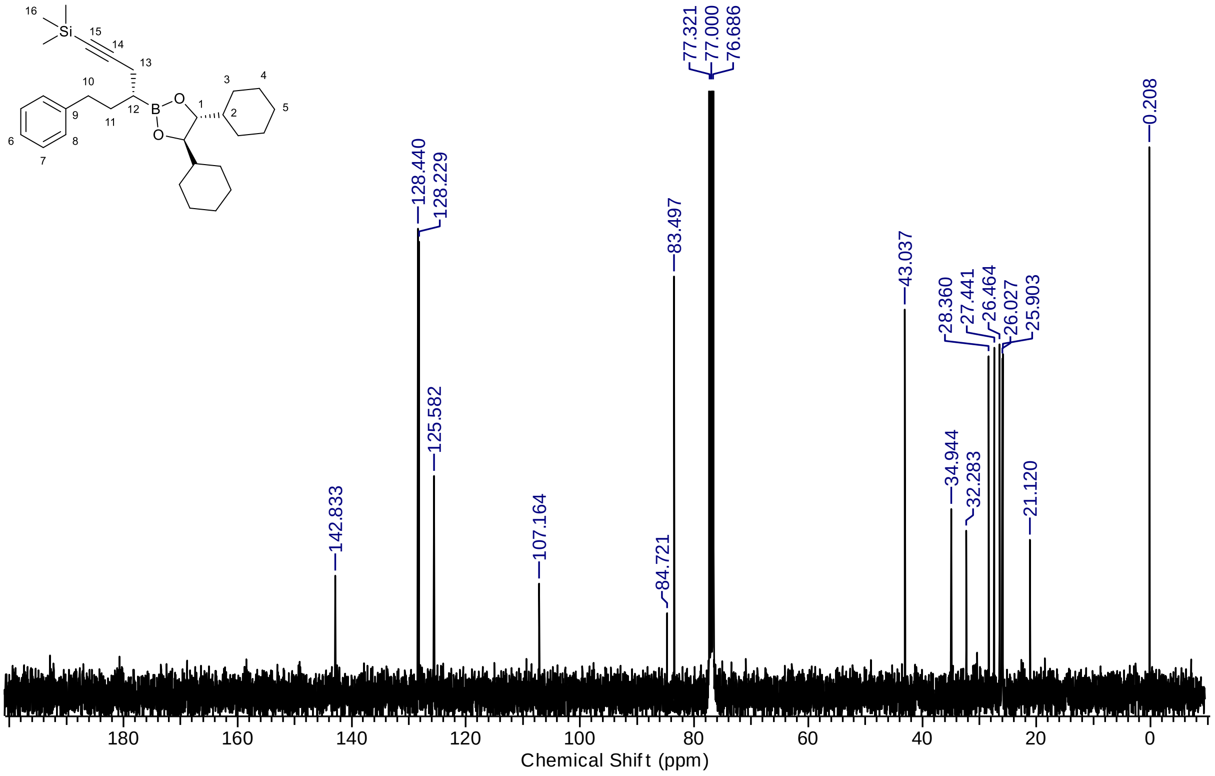

(4*R*,5*R*)-4,5-Dicyclohexyl-2-((*R*)-1-phenylhex-5-yn-3-yl)-1,3,2-dioxaborolane (3)

^{1}H-NMR (400 MHz, $CDCl_3$):

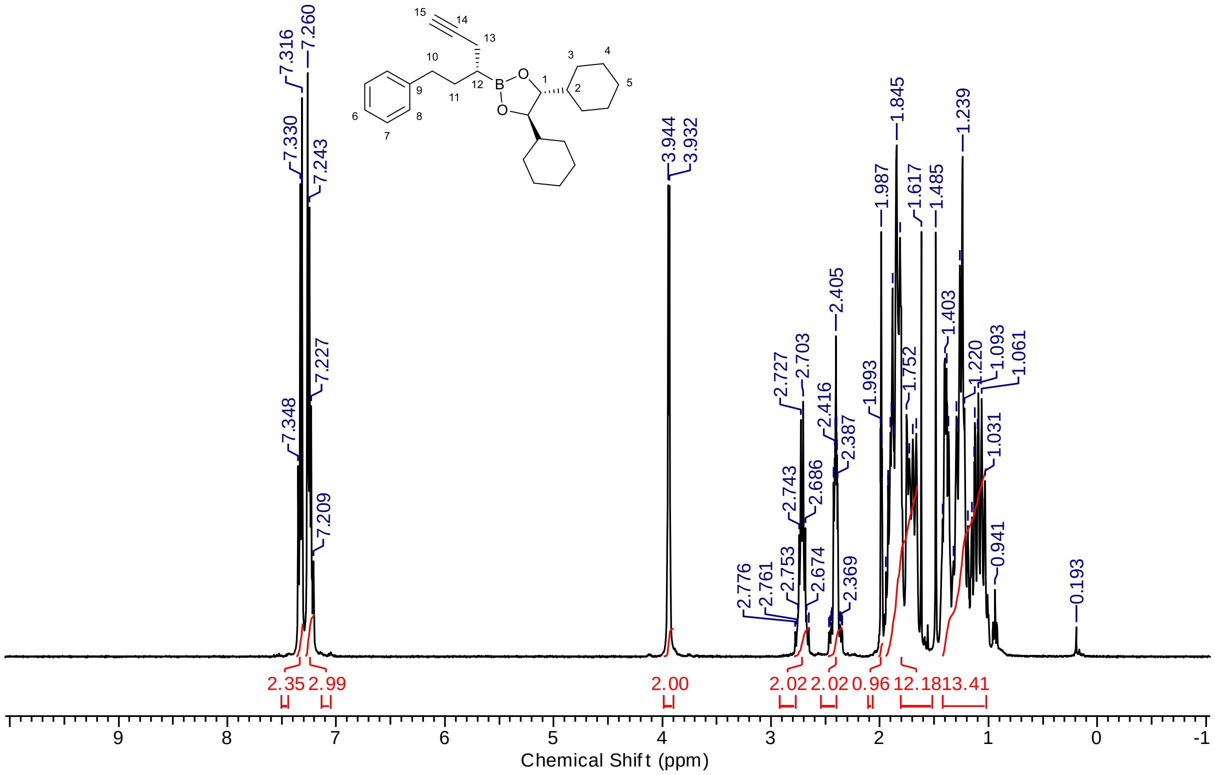

^{13}C-NMR (100 MHz, $CDCl_3$):

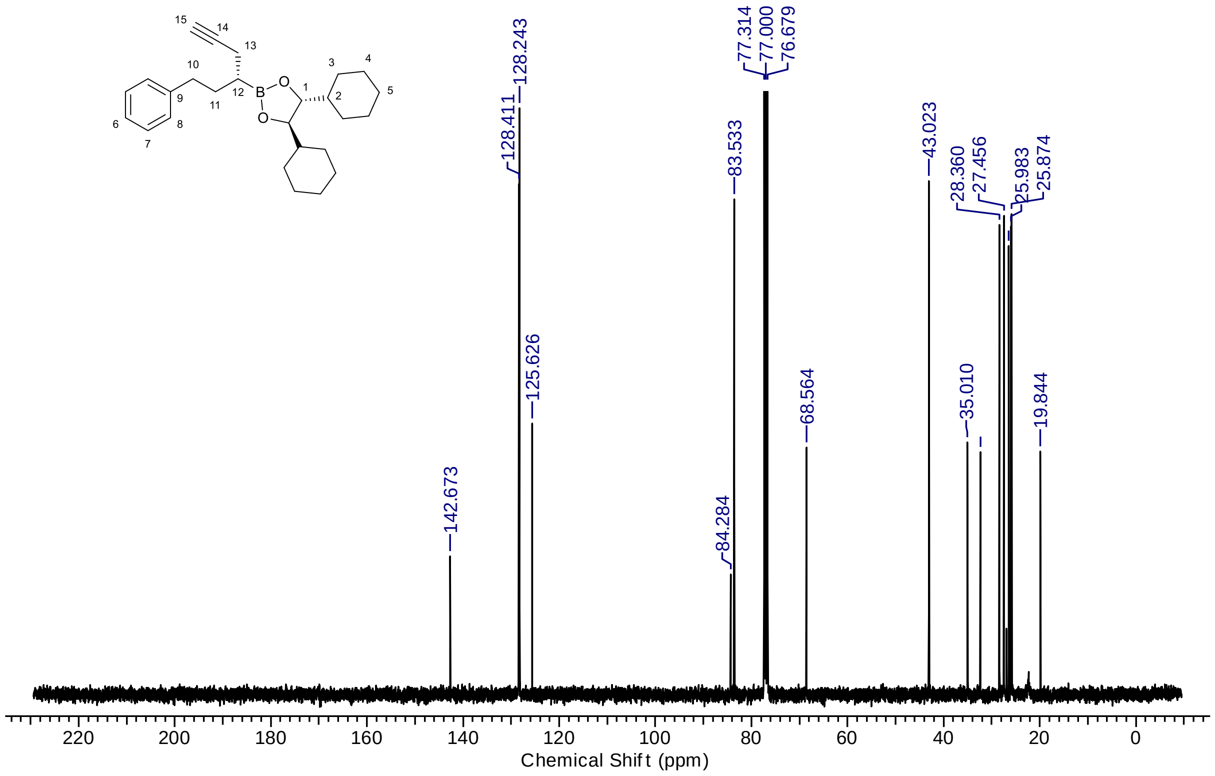

(4*R*,5*R*)-4,5-dicyclohexyl-2-((*R*,*E*)-7-(4-methoxyphenyl)-1-phenylhept-5-en-3-yl)-1,3,2-dioxaborolane (5h)

^{1}H-NMR (400 MHz, $CDCl_3$):

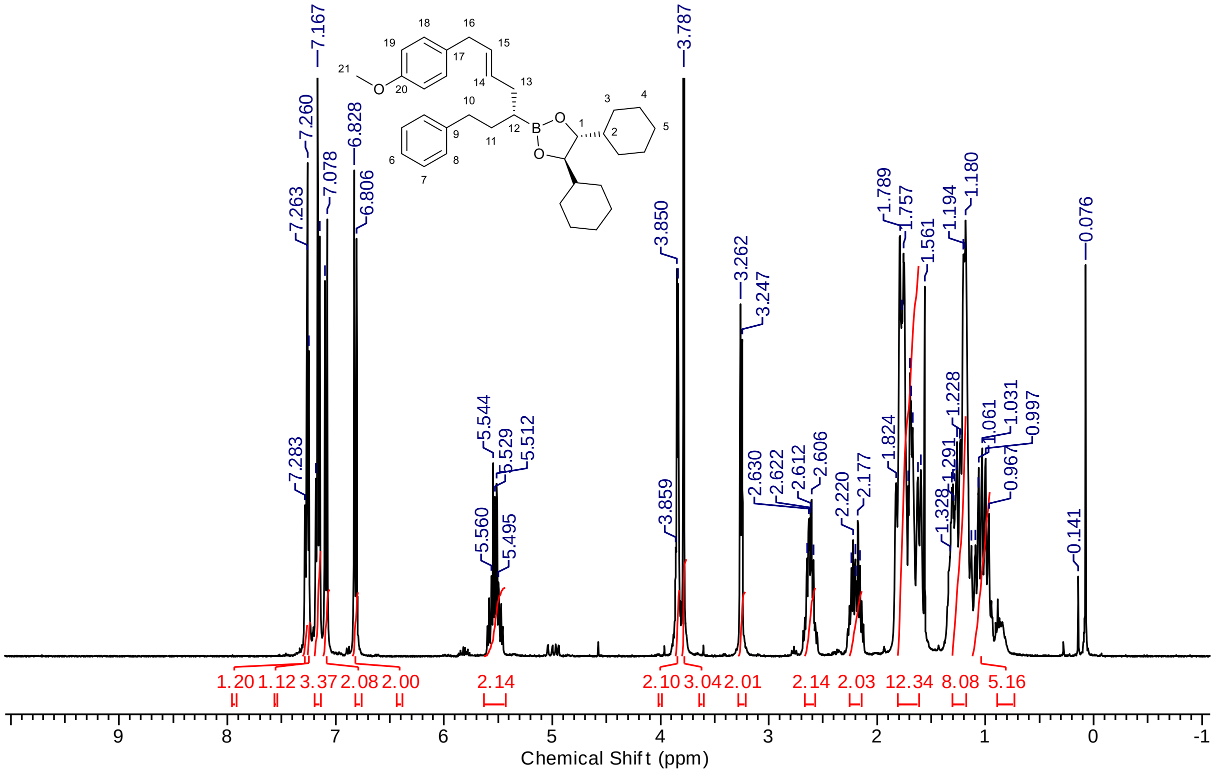

^{13}C-NMR (100 MHz, $CDCl_3$):

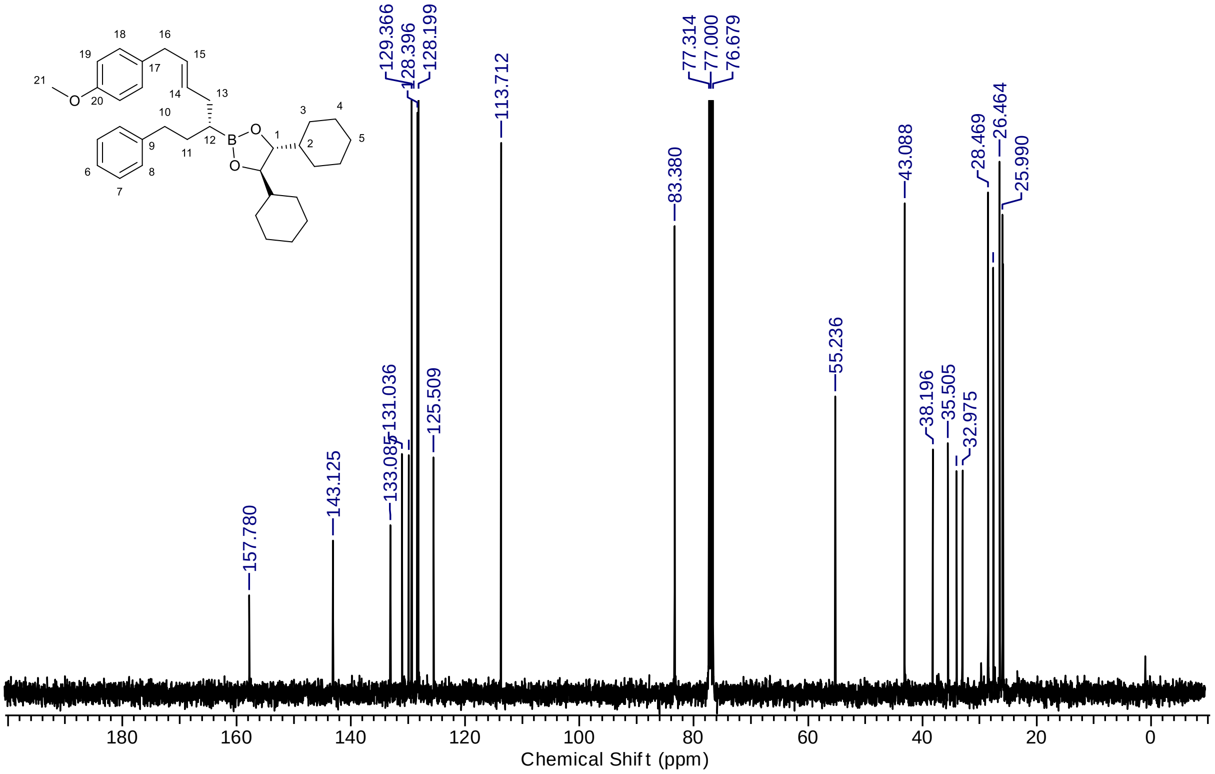

(*R*,*E*)-1,7-Diphenylhept-5-en-3-ol (6a)

^{1}H-NMR (400 MHz, $CDCl_3$):

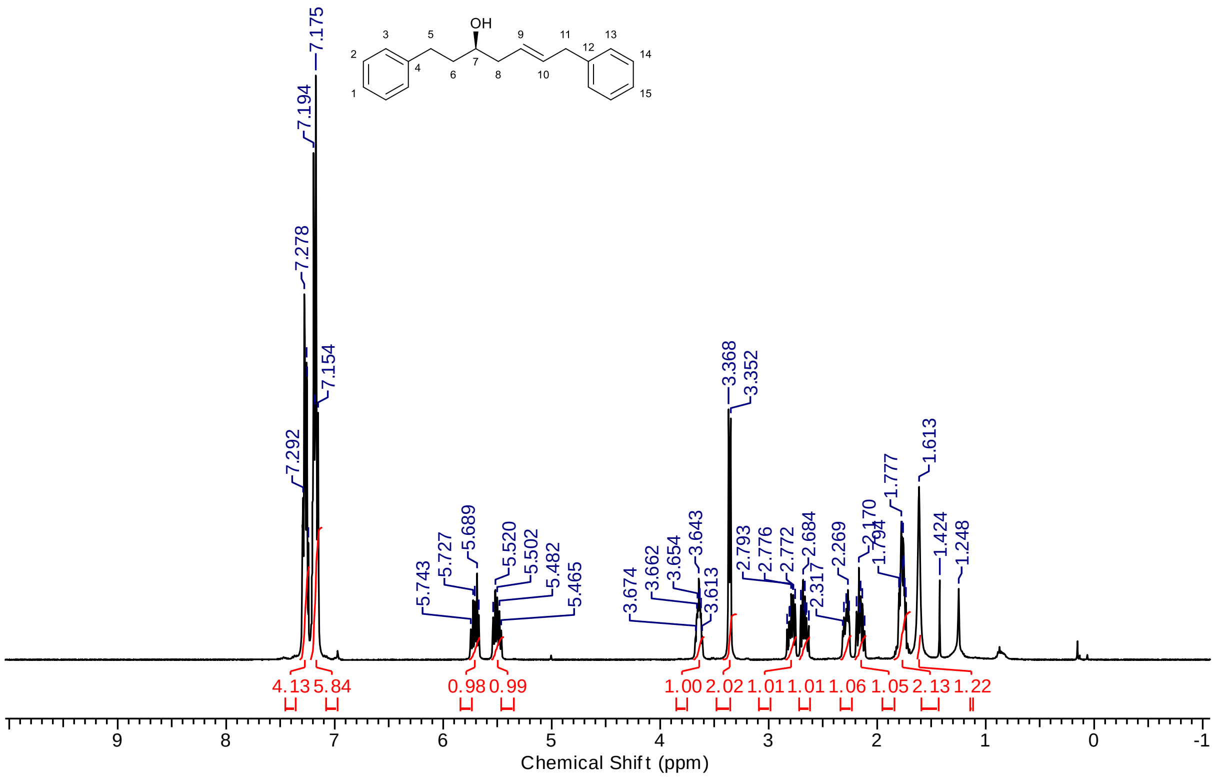

^{13}C-NMR (100 MHz, $CDCl_3$):

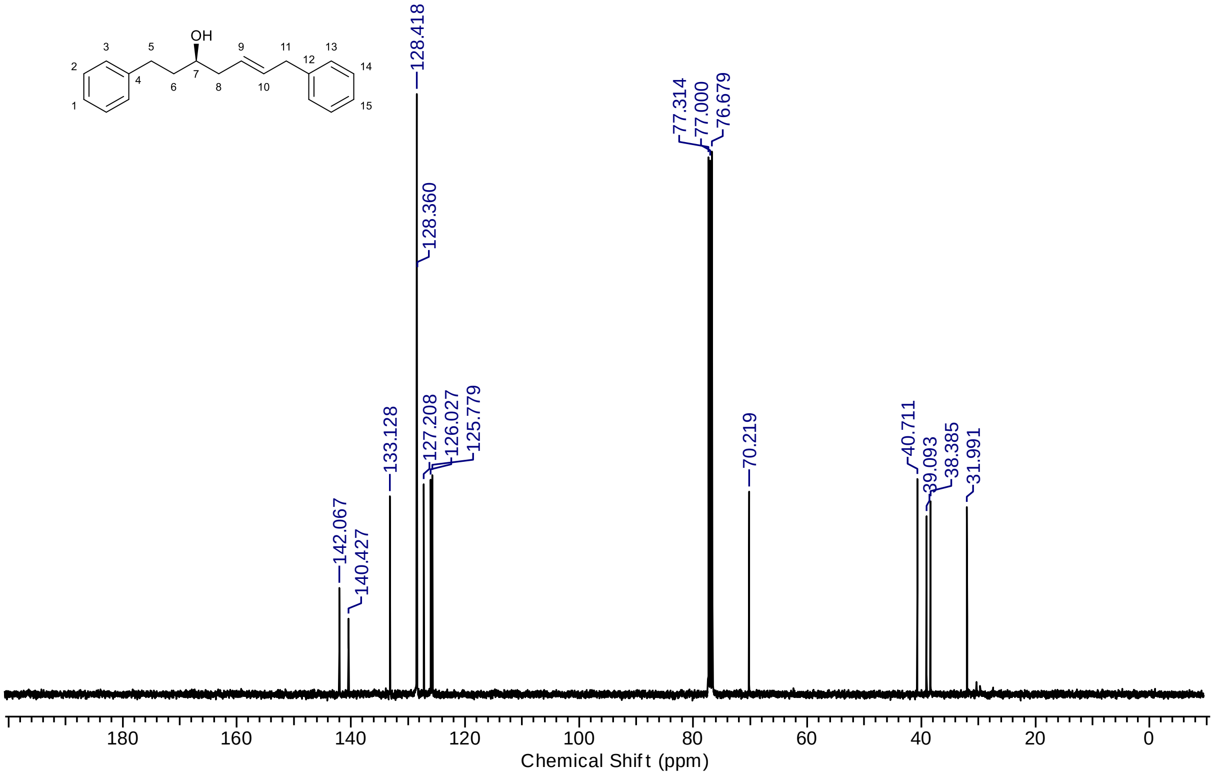

(*R*,*E*)-7-(4-Fluorophenyl)-1-phenylhept-5-en-3-ol (6b)

^{1}H-NMR (400 MHz, $CDCl_3$):

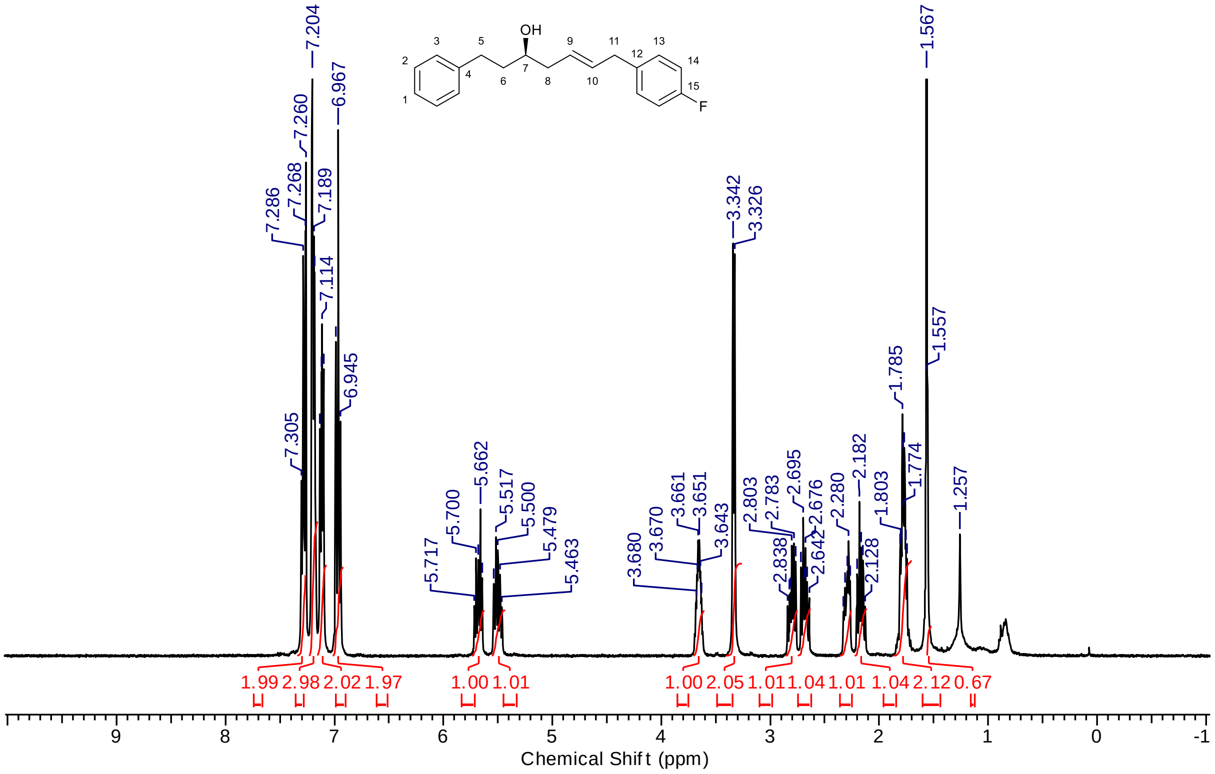

^{13}C-NMR (100 MHz, $CDCl_3$):

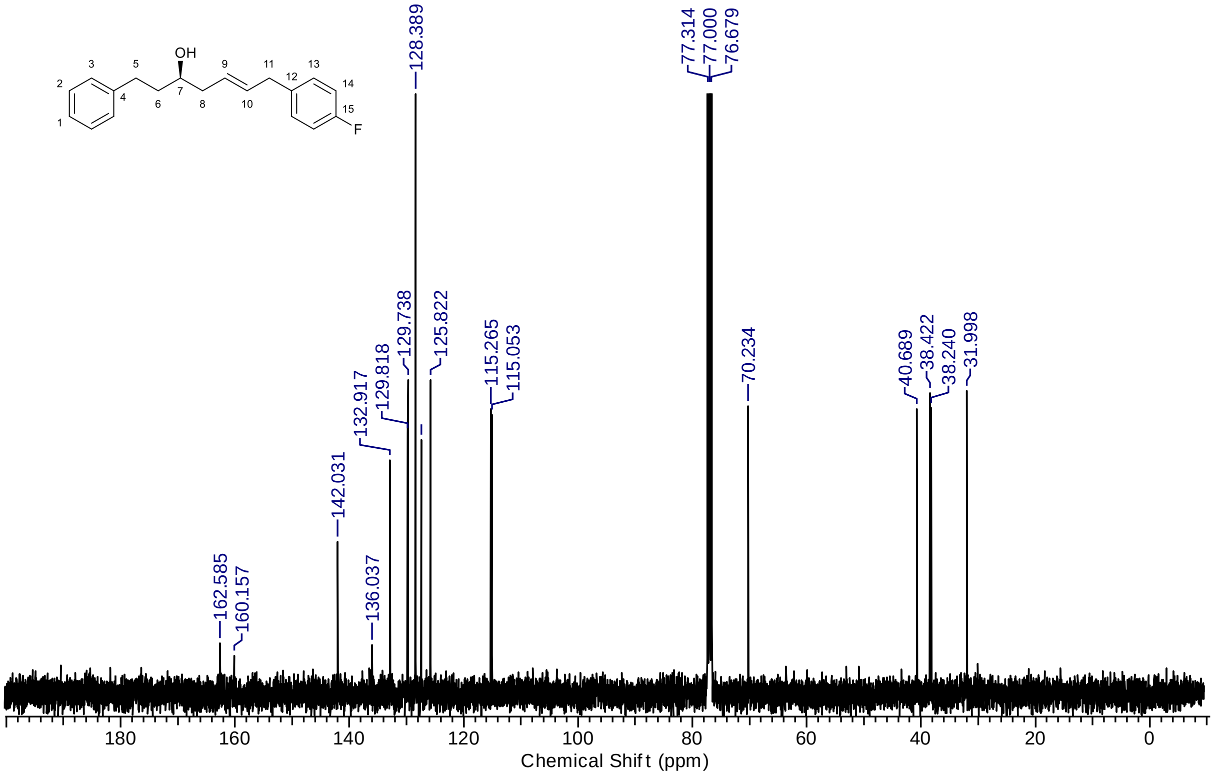

^{19}F-NMR (400 MHz, $CDCl_3$):

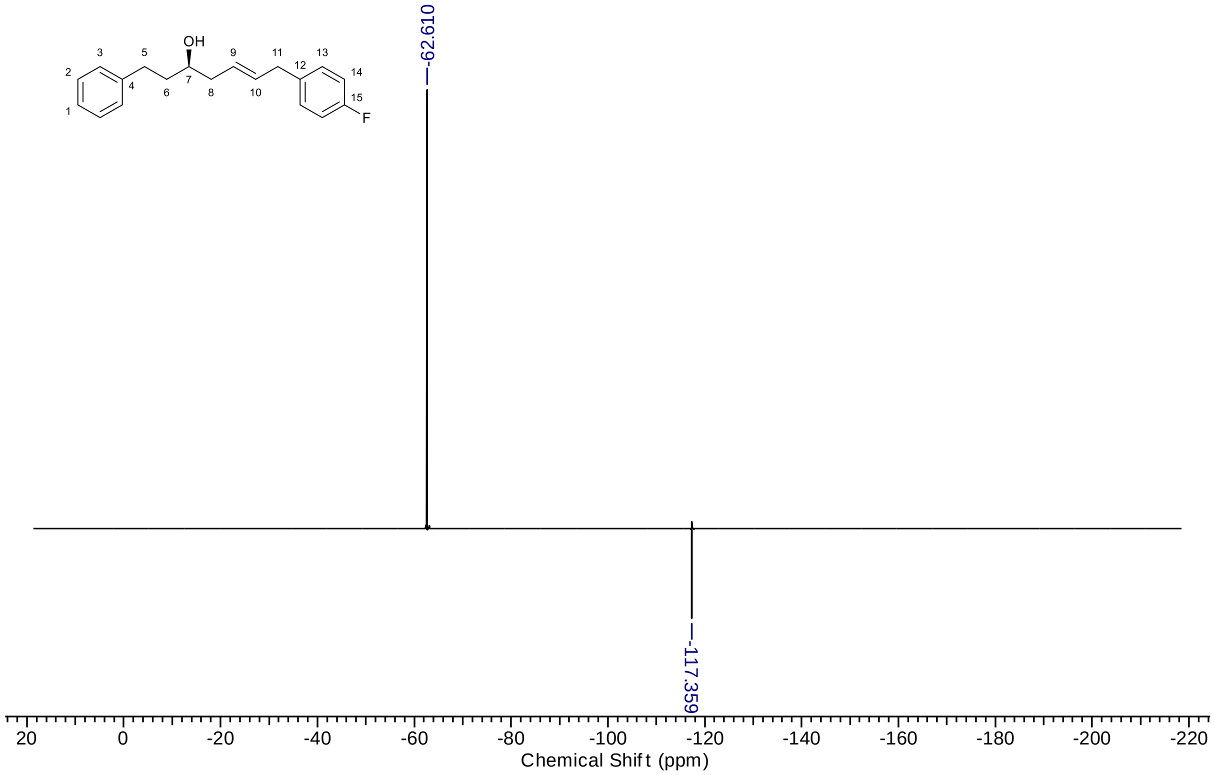

(*R*,*E*)-7-(4-Bromophenyl)-1-phenylhept-5-en-3-ol (6c)

^{1}H-NMR (400 MHz, $CDCl_3$):

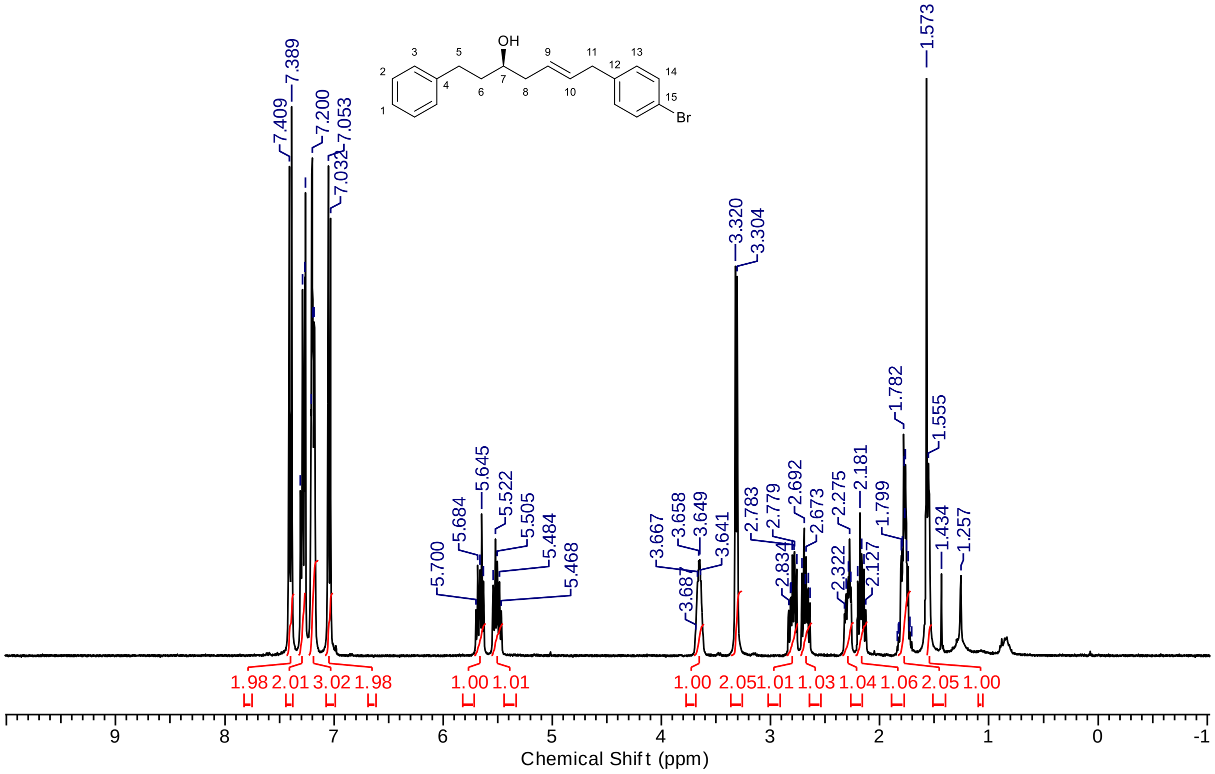

^{13}C-NMR (100 MHz, $CDCl_3$):

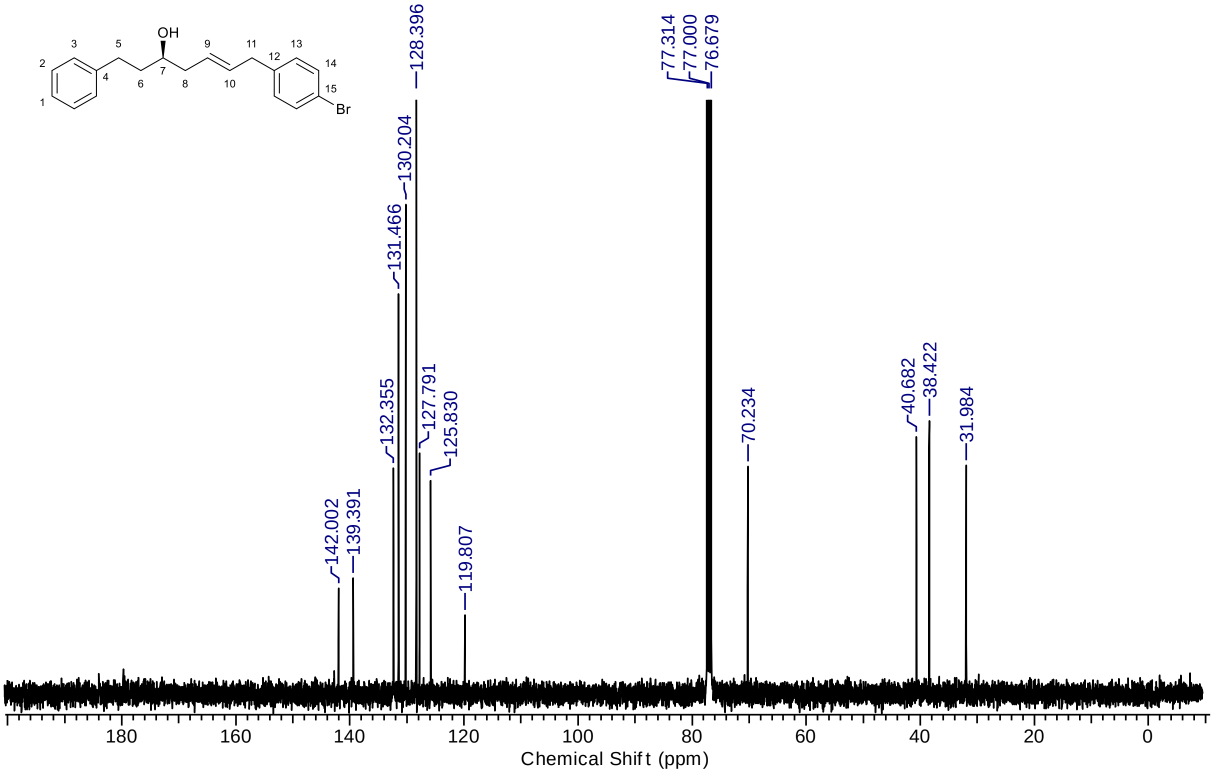

(*R*,*E*)-1-Phenyl-7-(*p*-tolyl)hept-5-en-3-ol (6d)

^{1}**H-NMR** (400 MHz, $CDCl_3$):

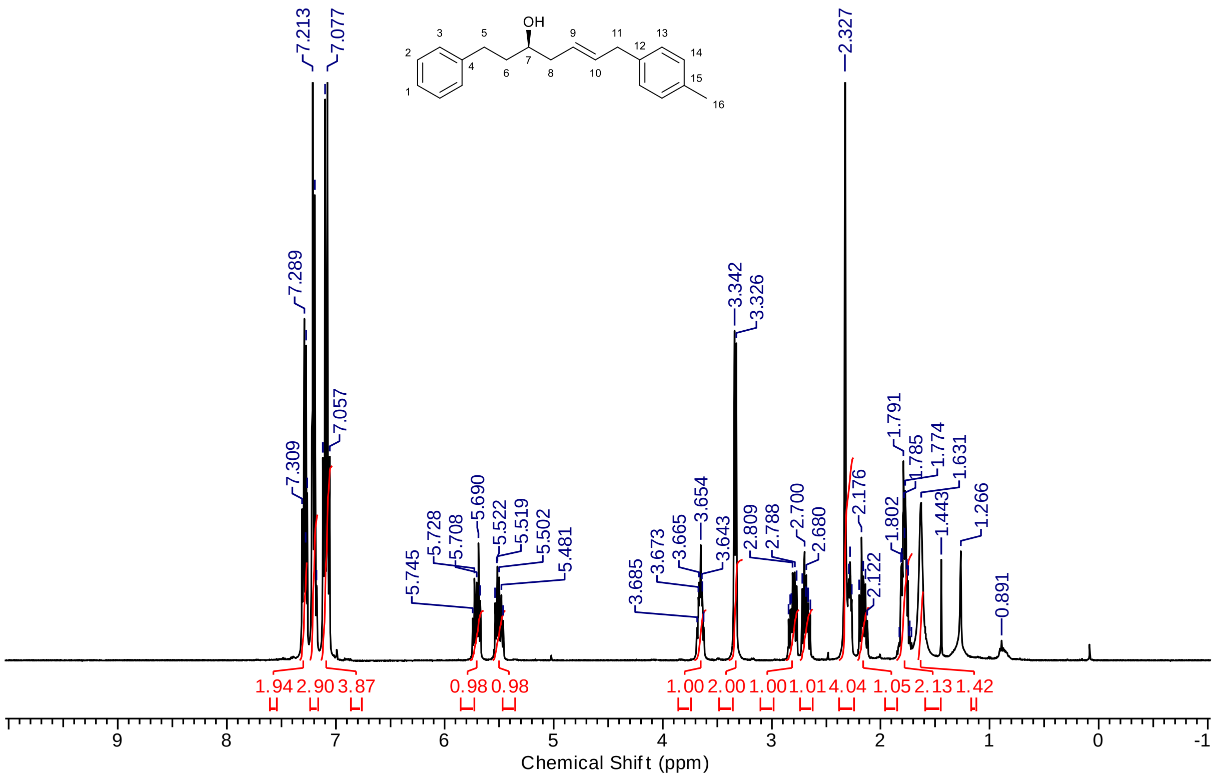

^{13}**C-NMR** (100 MHz, $CDCl_3$):

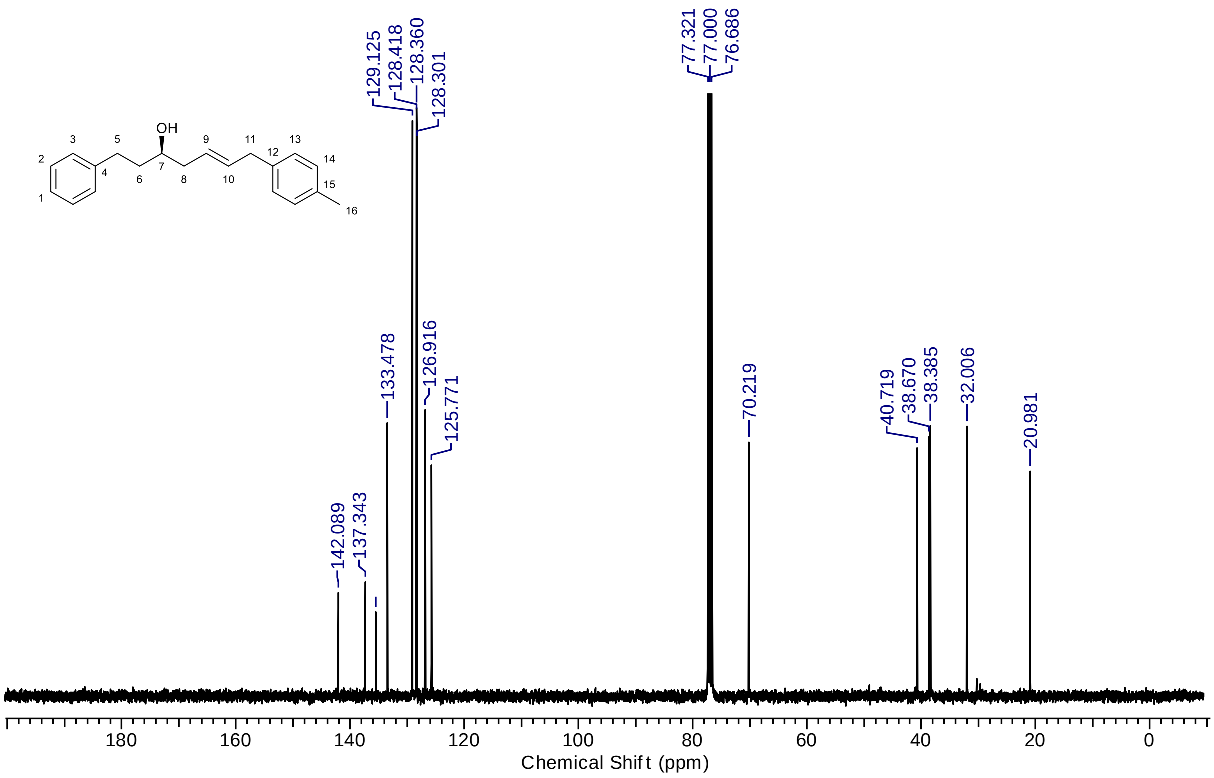

(*R*,*E*)-1-Phenyl-7-(*o*-tolyl)hept-5-en-3-ol (6e)

^{1}H-NMR (400 MHz, $CDCl_3$):

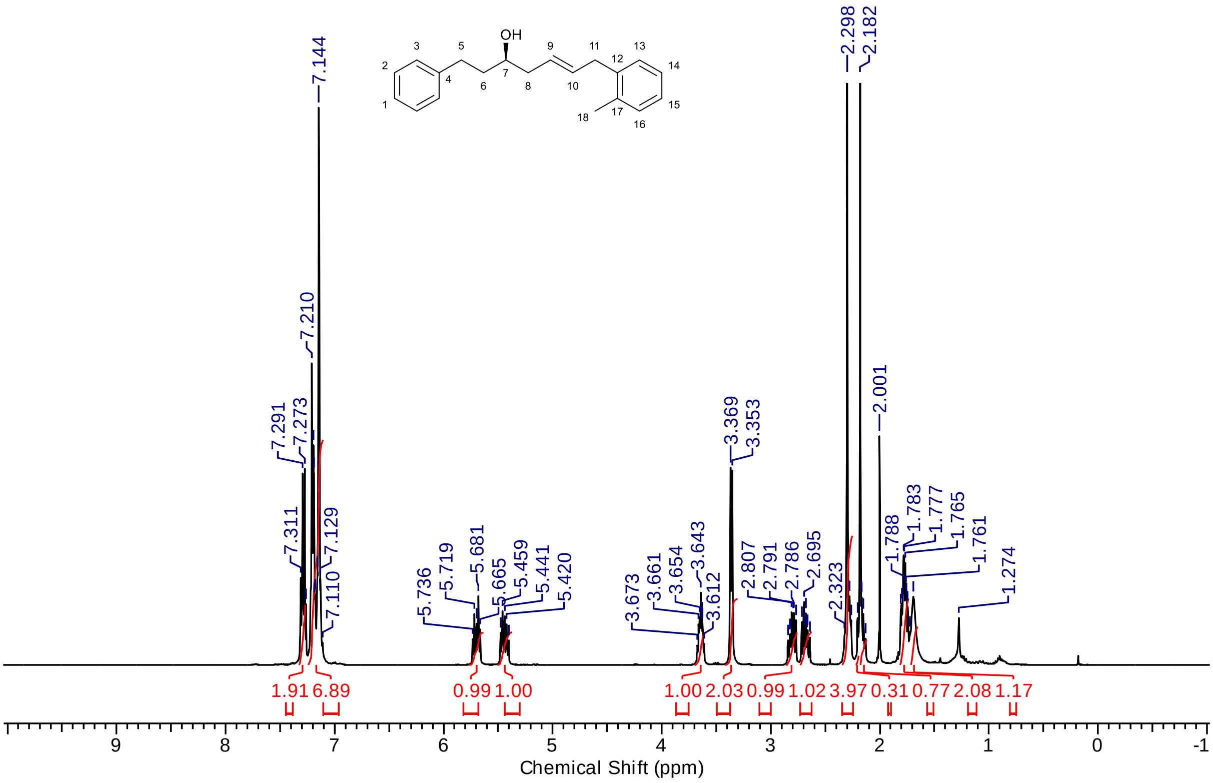

^{13}C-NMR (100 MHz, $CDCl_3$):

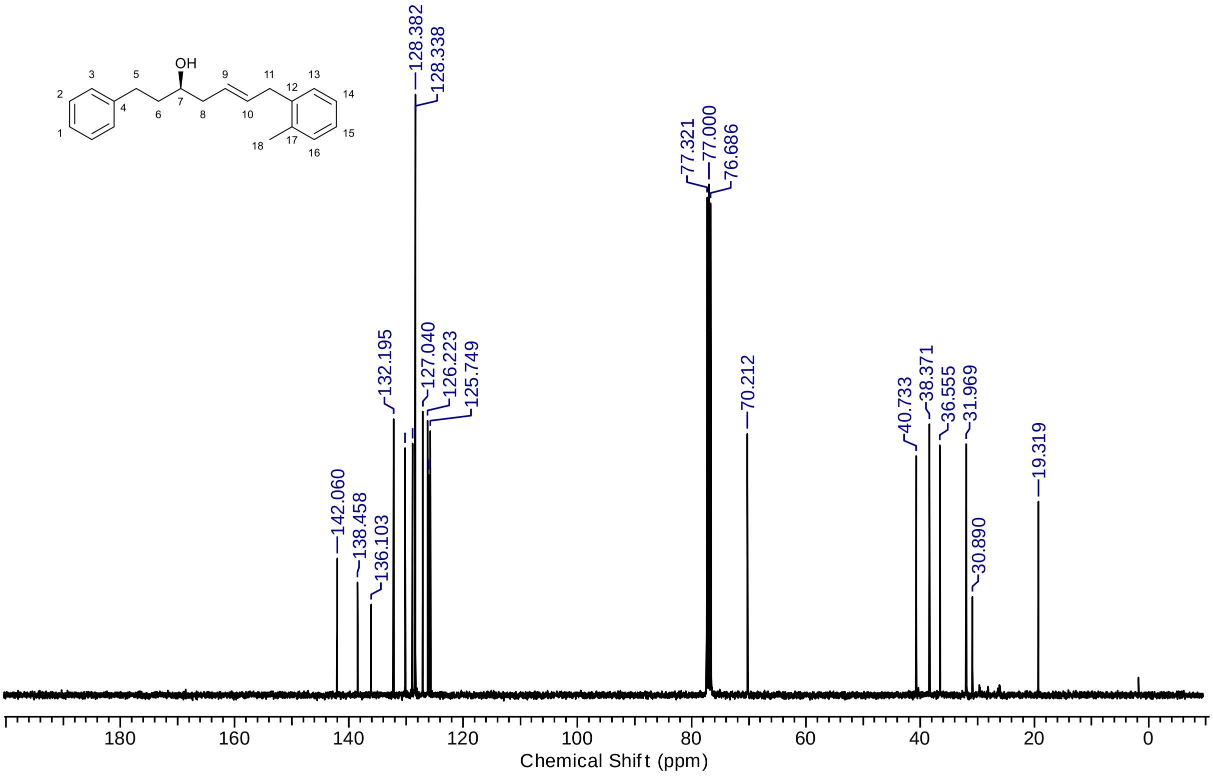

(*R*,*E*)-7-(Benzo[d][1,3]dioxol-5-yl)-1-phenylhept-5-en-3-ol (6f)

^{1}H-NMR (400 MHz, $CDCl_3$):

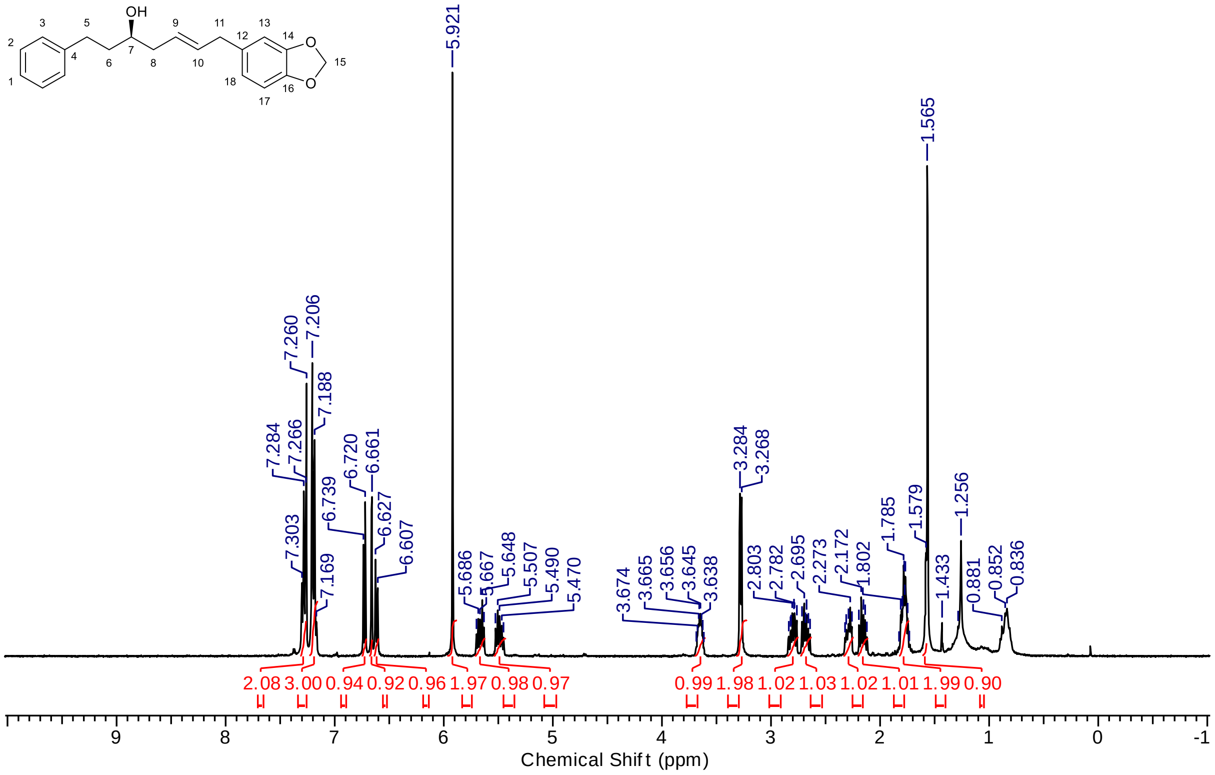

^{13}C-NMR (100 MHz, $CDCl_3$):

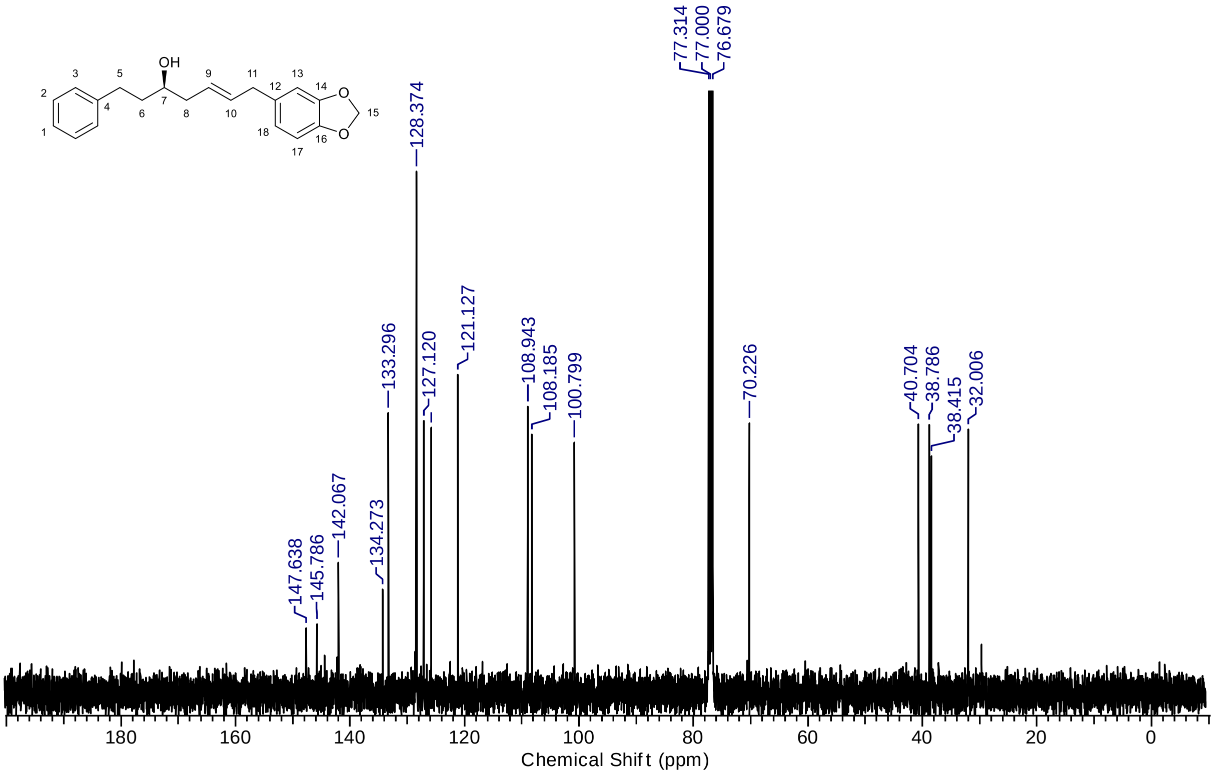

(*R*,*E*)-1-phenylnona-5,8-dien-3-ol (6g)

^{1}H-NMR (400 MHz, $CDCl_3$):

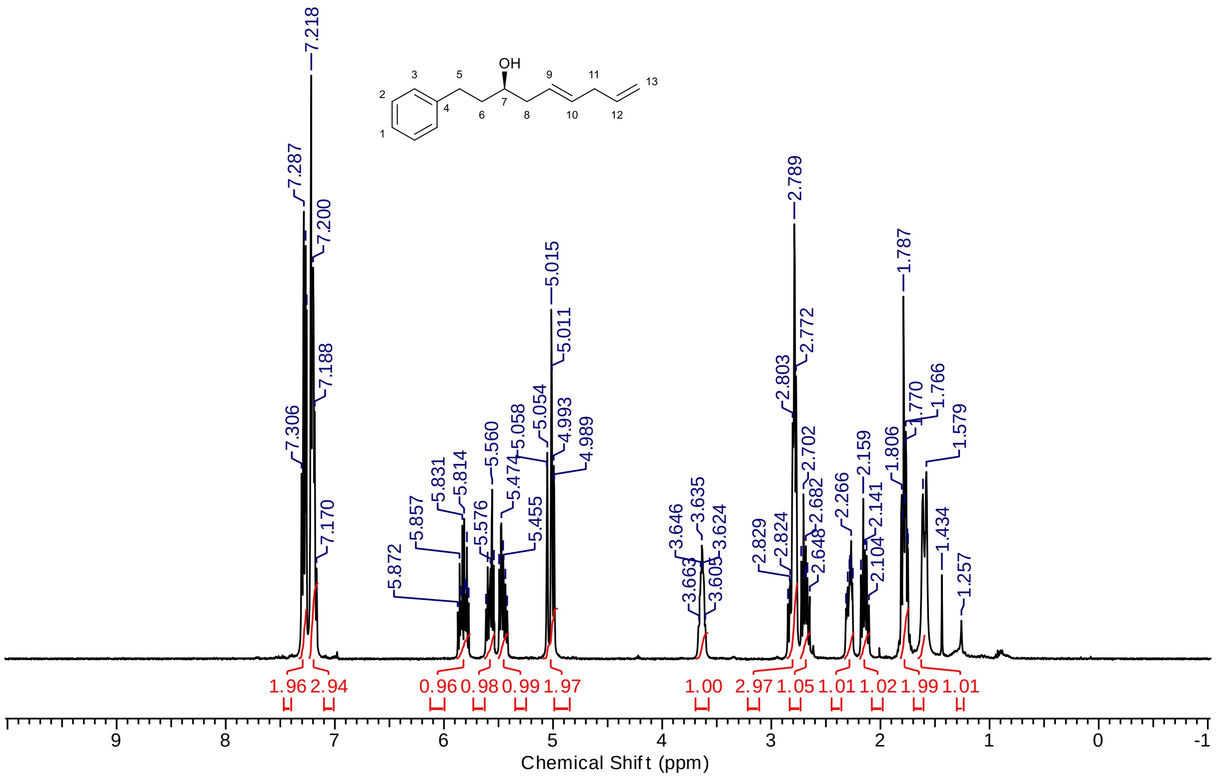

^{13}C-NMR (100 MHz, $CDCl_3$):

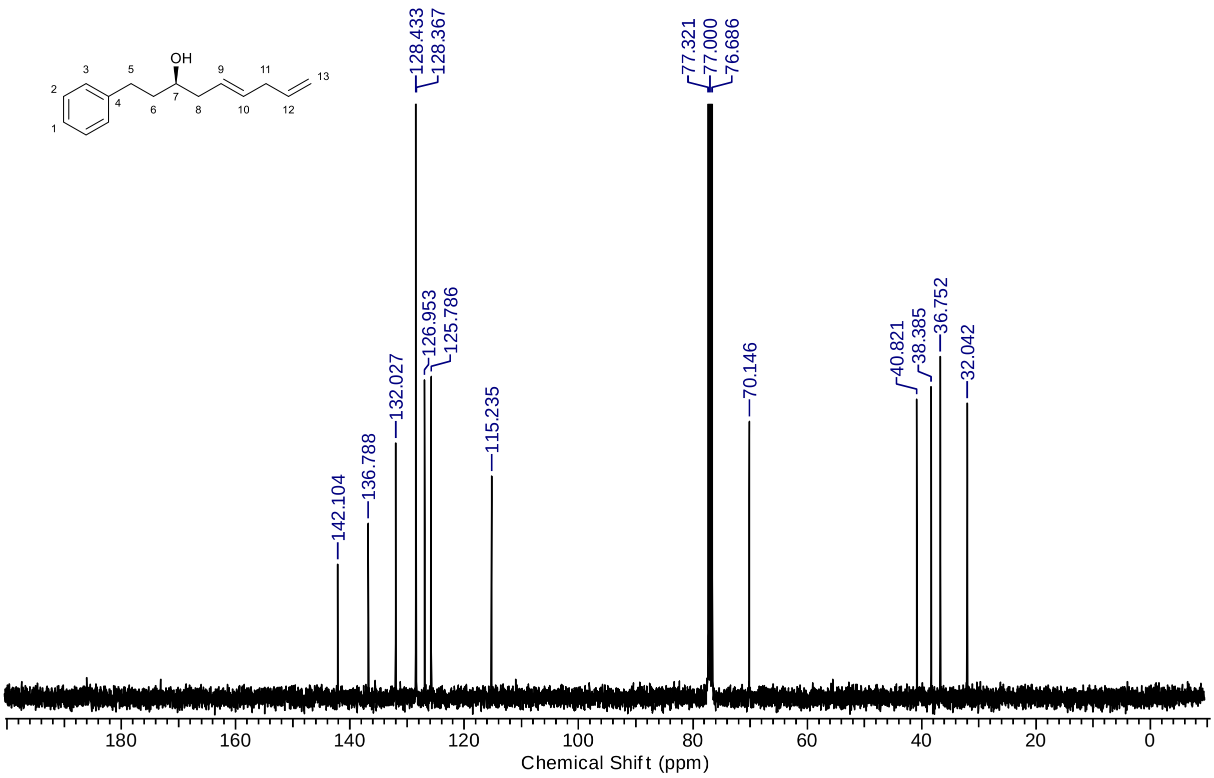

(*R*,*E*)-7-(4-(3-Hydroxyprop-1-yn-1-yl)phenyl)-1-phenylhept-5-en-3-ol (6i)

^{1}H-NMR (500 MHz, $CDCl_3$):

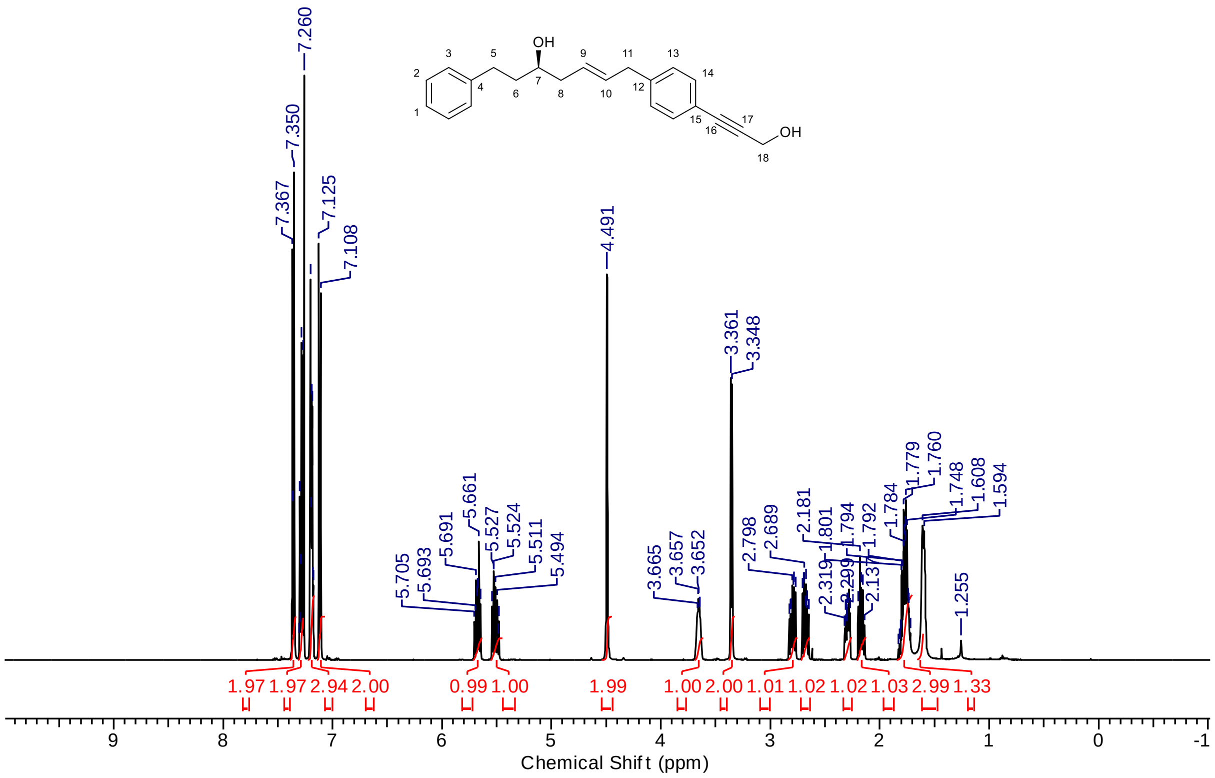

^{13}C-NMR (125 MHz, $CDCl_3$):

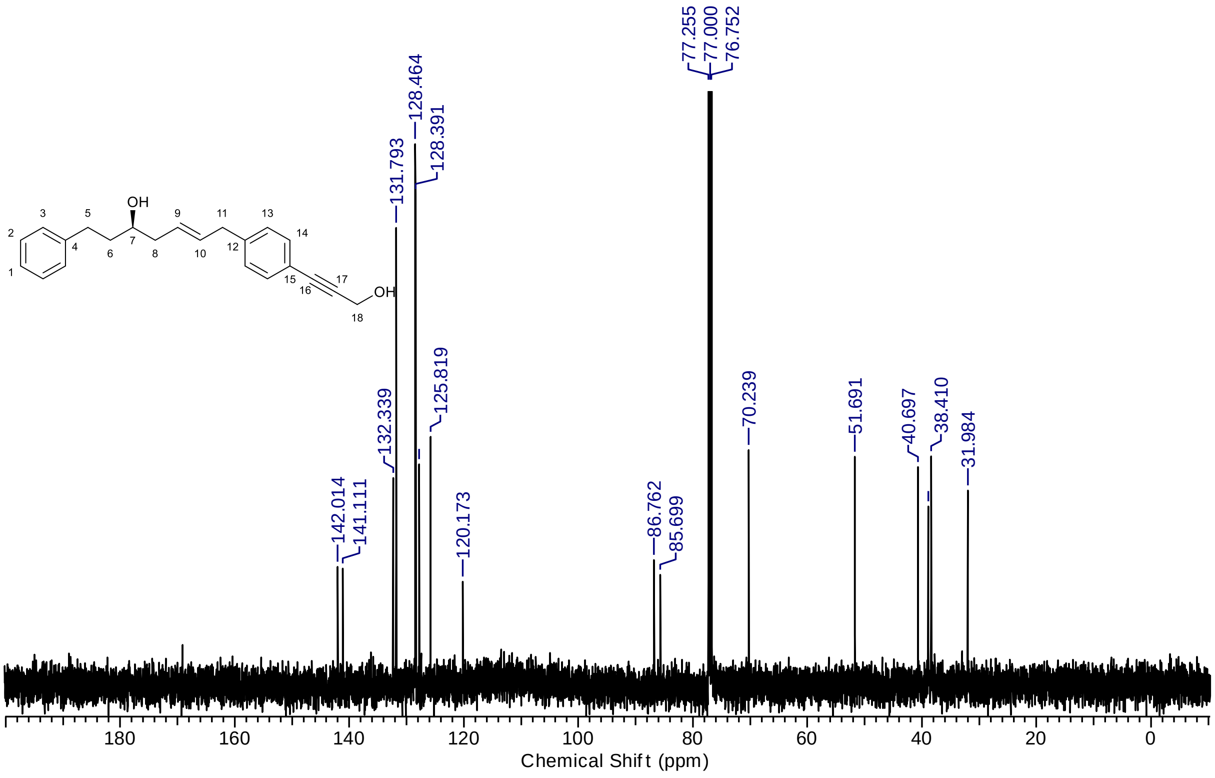

(*R*,*E*)-1,6-Diphenylhex-5-en-3-ol (8a)

^{1}H-NMR (400 MHz, $CDCl_3$):

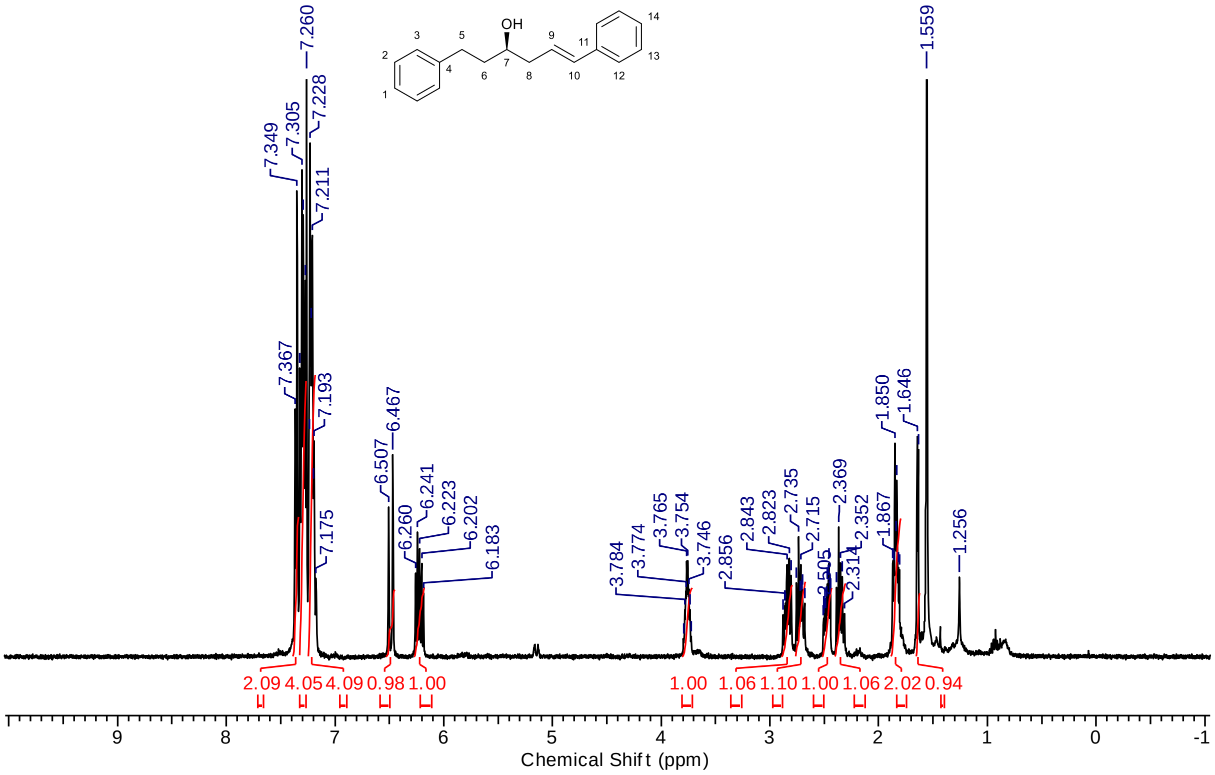

^{13}C-NMR (100 MHz, $CDCl_3$):

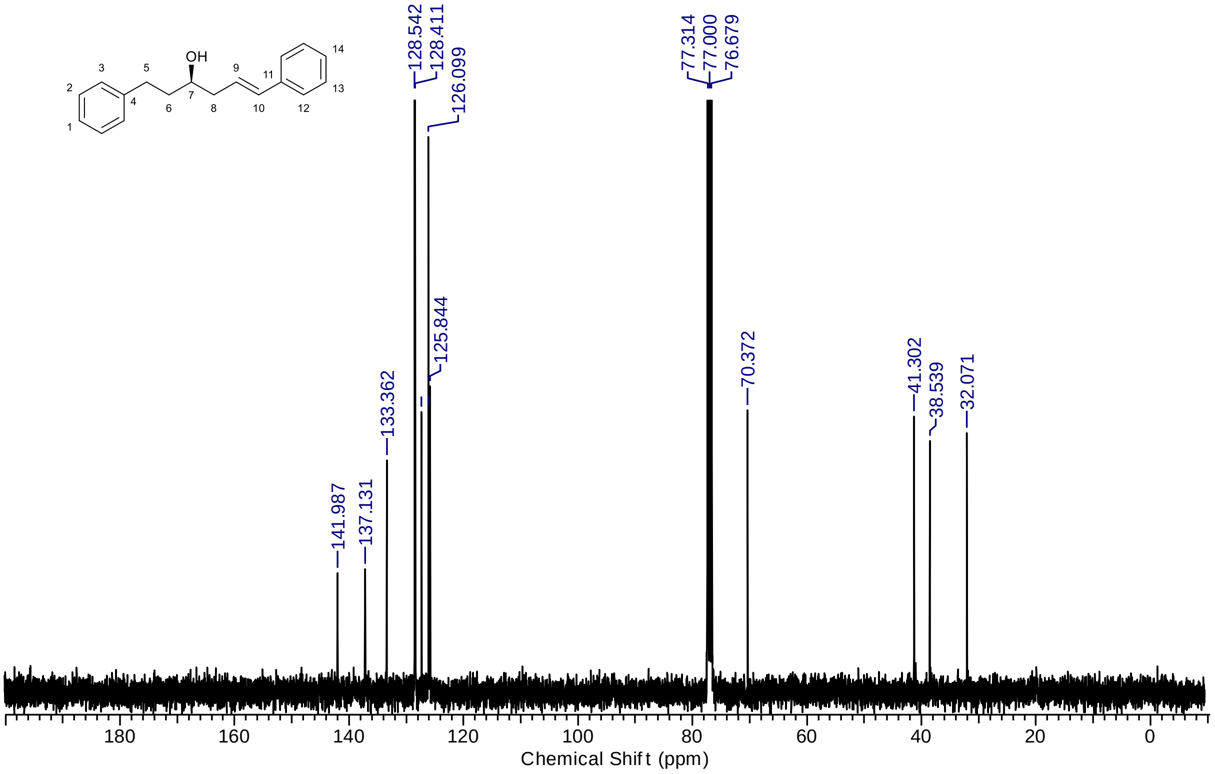

(*R*,*E*)-1-Phenyl-6-(*p*-tolyl)hex-5-en-3-ol (8b)

^{1}H-NMR (400 MHz, $CDCl_3$):

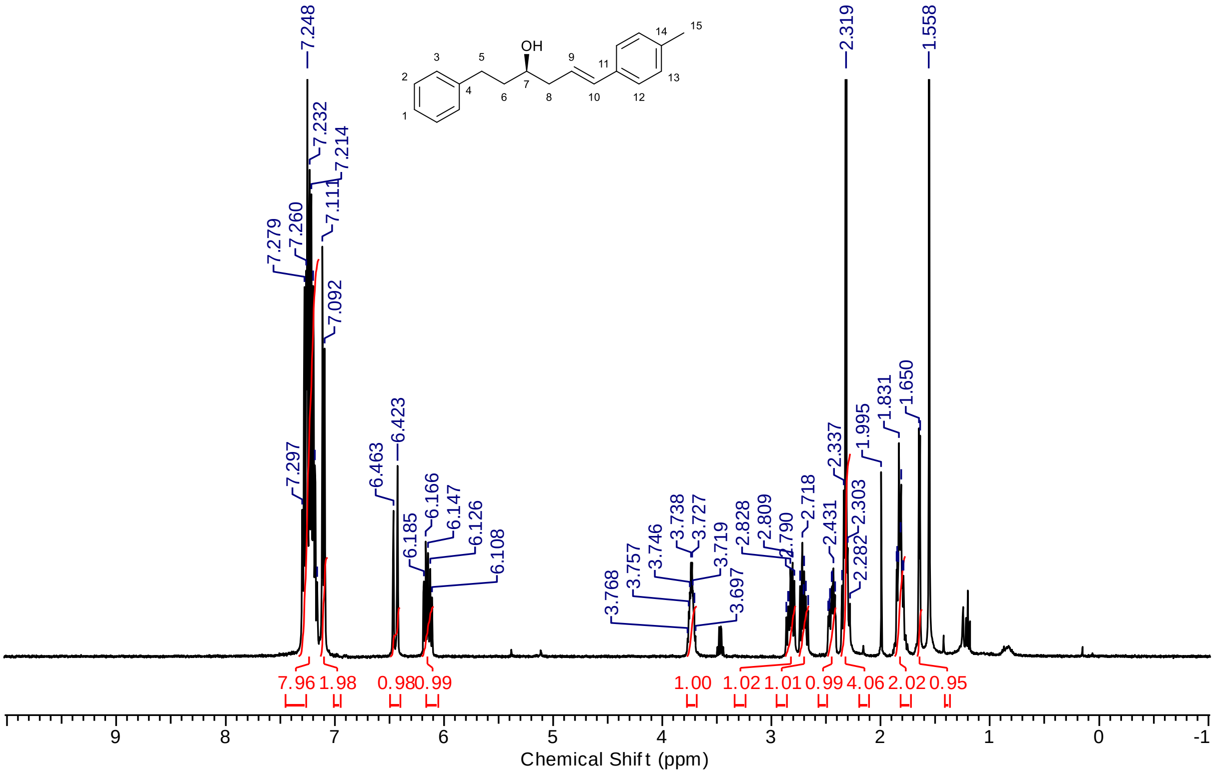

^{13}C-NMR (100 MHz, $CDCl_3$):

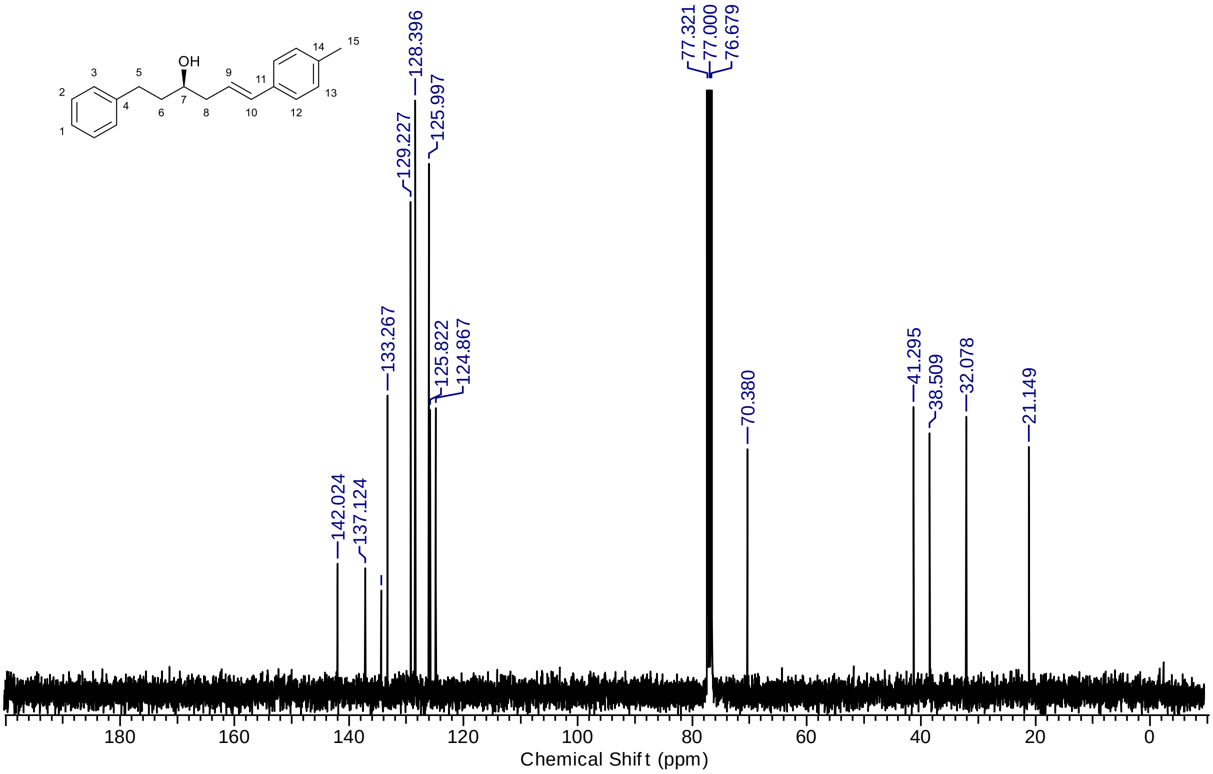

(*R*,*E*)-6-(4-Methoxyphenyl)-1-phenylhex-5-en-3-ol (8c)

^{1}H-NMR (400 MHz, $CDCl_3$):

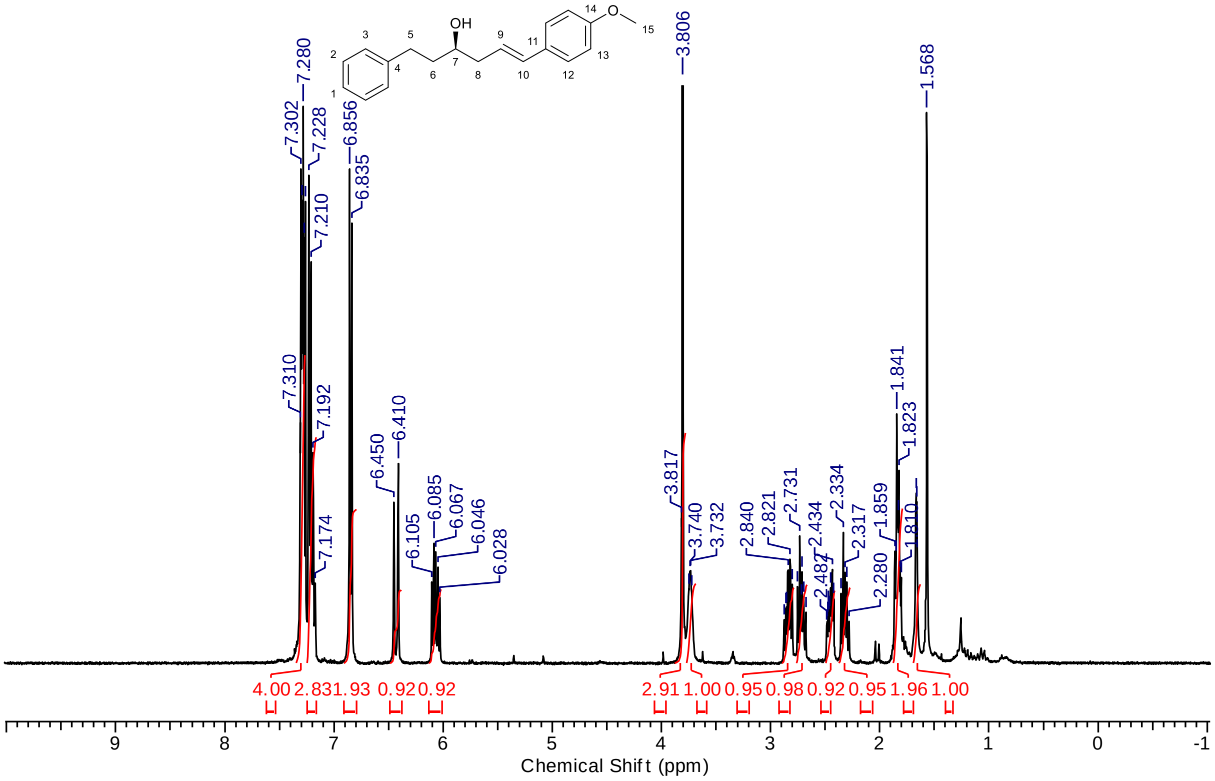

^{13}C-NMR (100 MHz, $CDCl_3$):

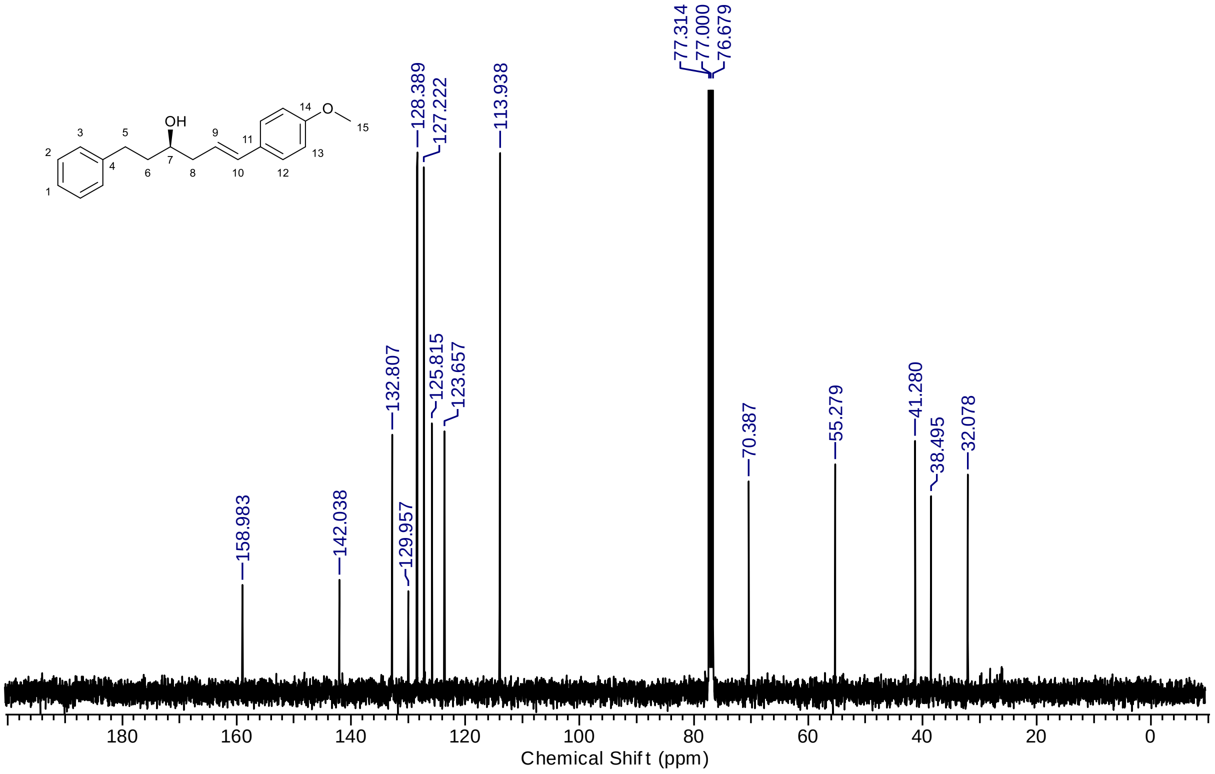

(*R*,*E*)-6-(4-Fluorophenyl)-1-phenylhex-5-en-3-ol (8d)

^{1}H-NMR (400 MHz, $CDCl_3$):

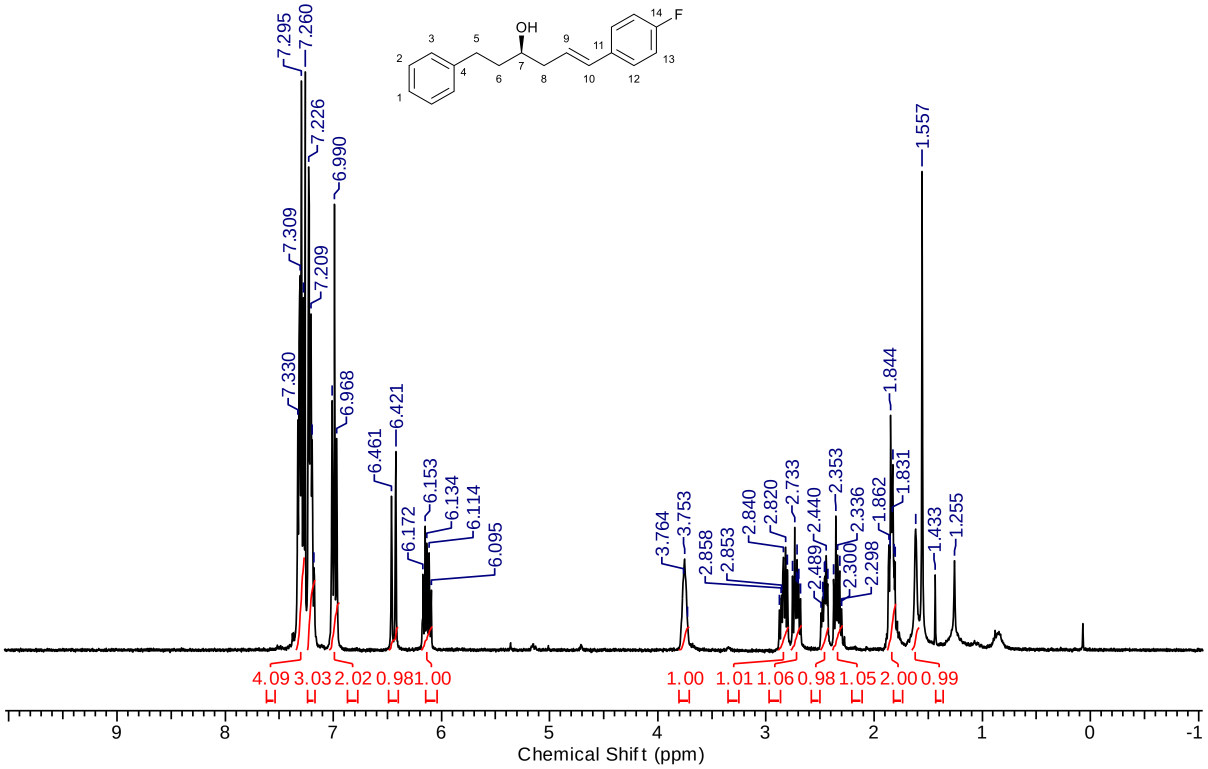

^{13}C-NMR (100 MHz, $CDCl_3$):

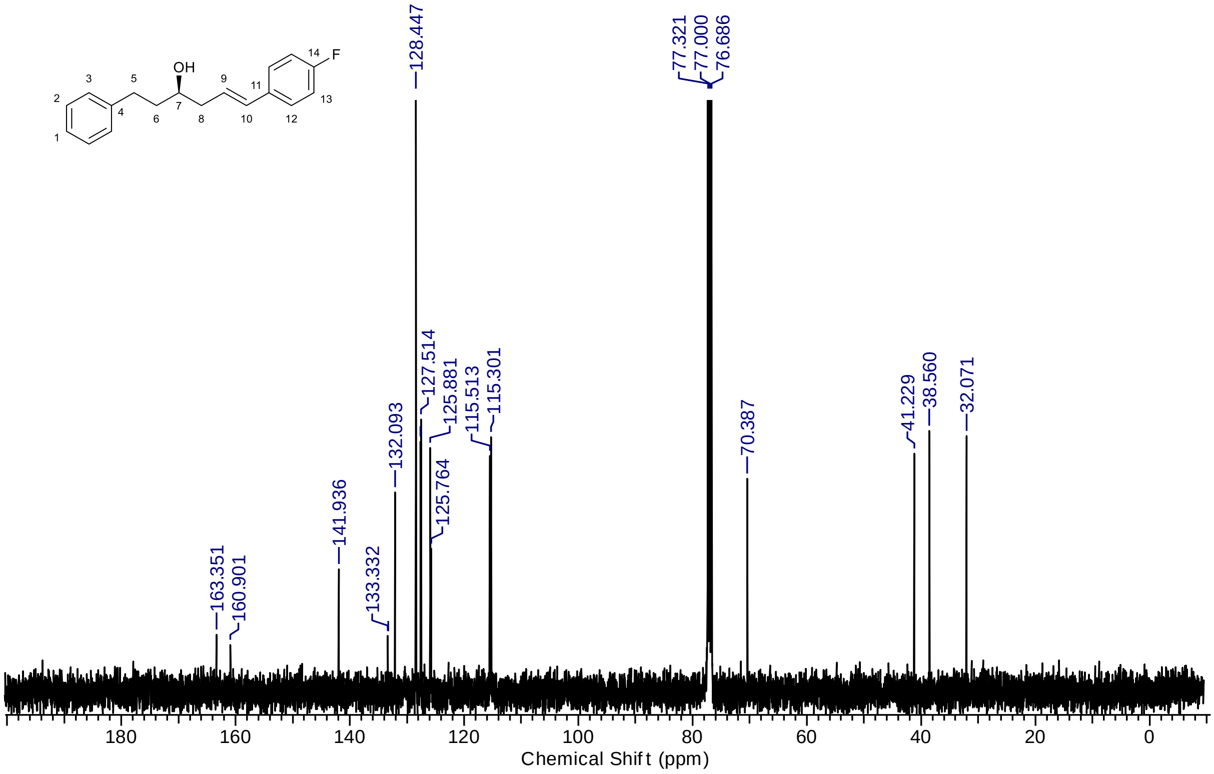

^{19}F-NMR (400 MHz, $CDCl_3$):

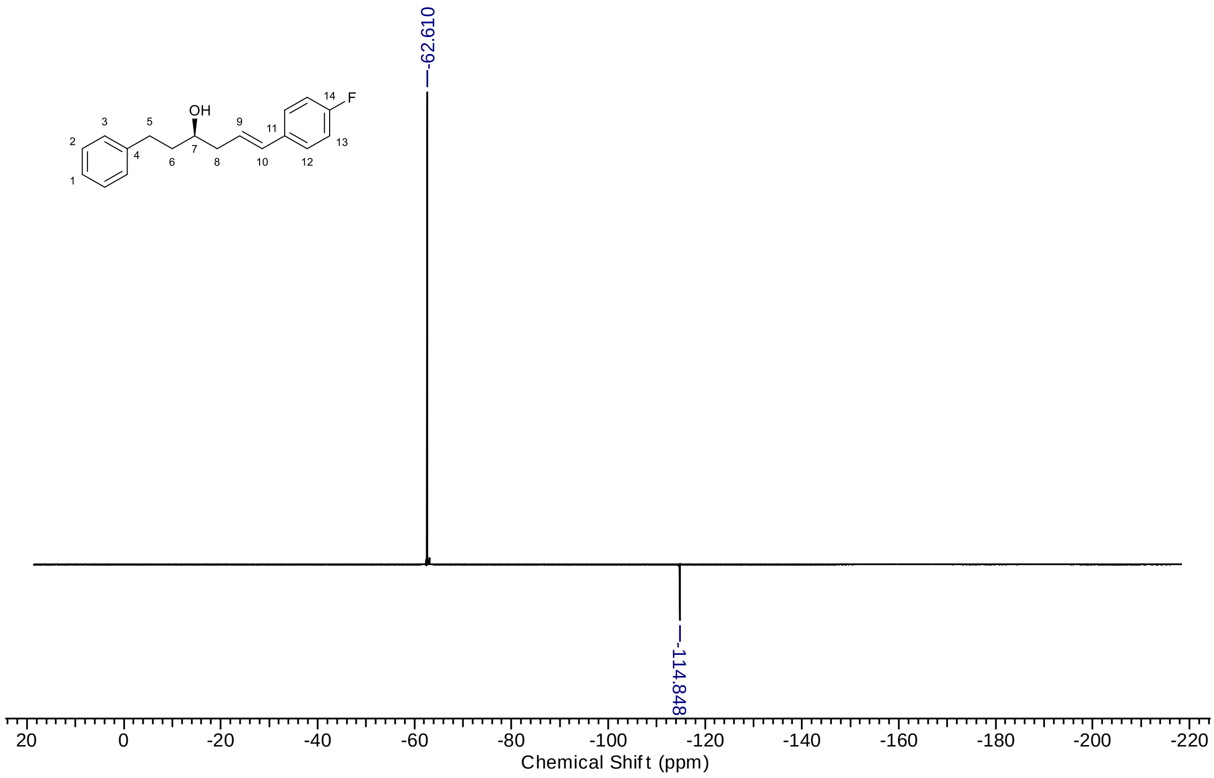
OH
F
1
2
3
4
5
6
7
8
9
10
11
12
13
14
-62.610
-114.848
20
0
-20
-40
-60
-80
-100
-120
-140
-160
-180
-200
-220
Chemical Shift (ppm)

Ethyl (*R*,*E*)-2-(4-hydroxy-6-phenylhex-1-en-1-yl)benzoate (8e)

^{1}H-NMR (400 MHz, $CDCl_3$):

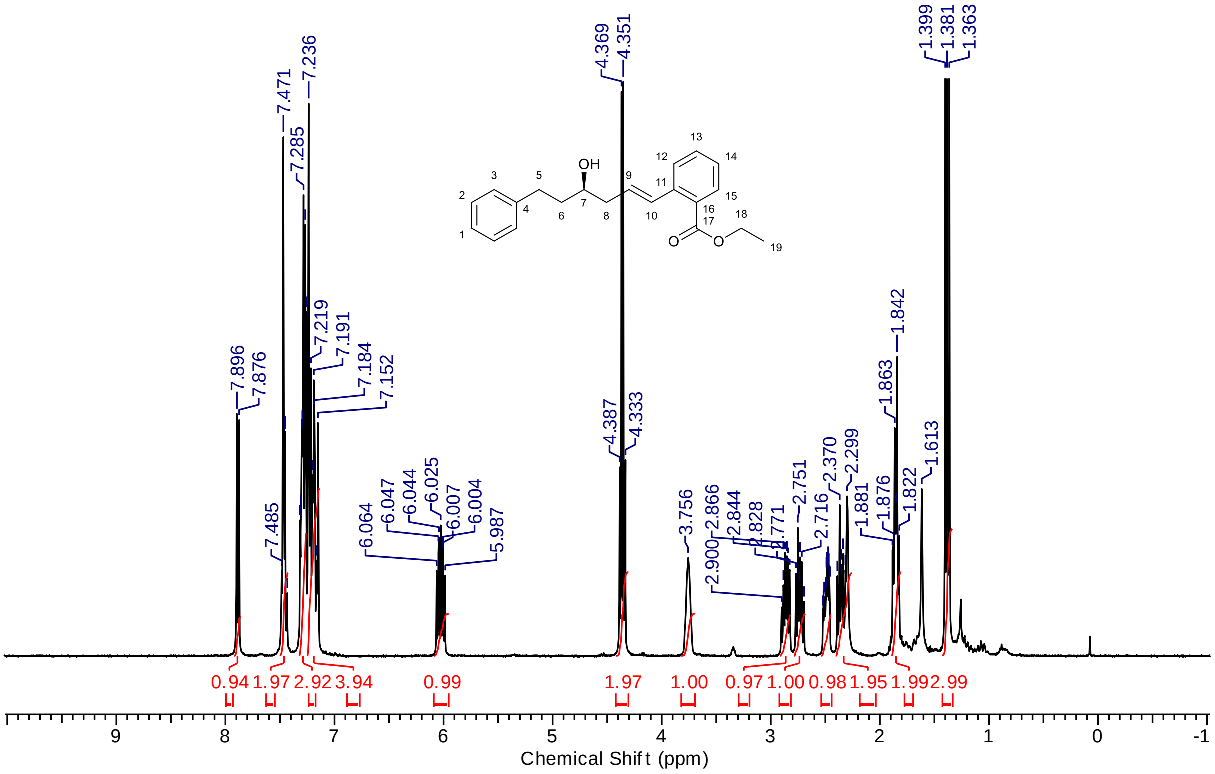

^{13}C-NMR (400 MHz, $CDCl_3$):

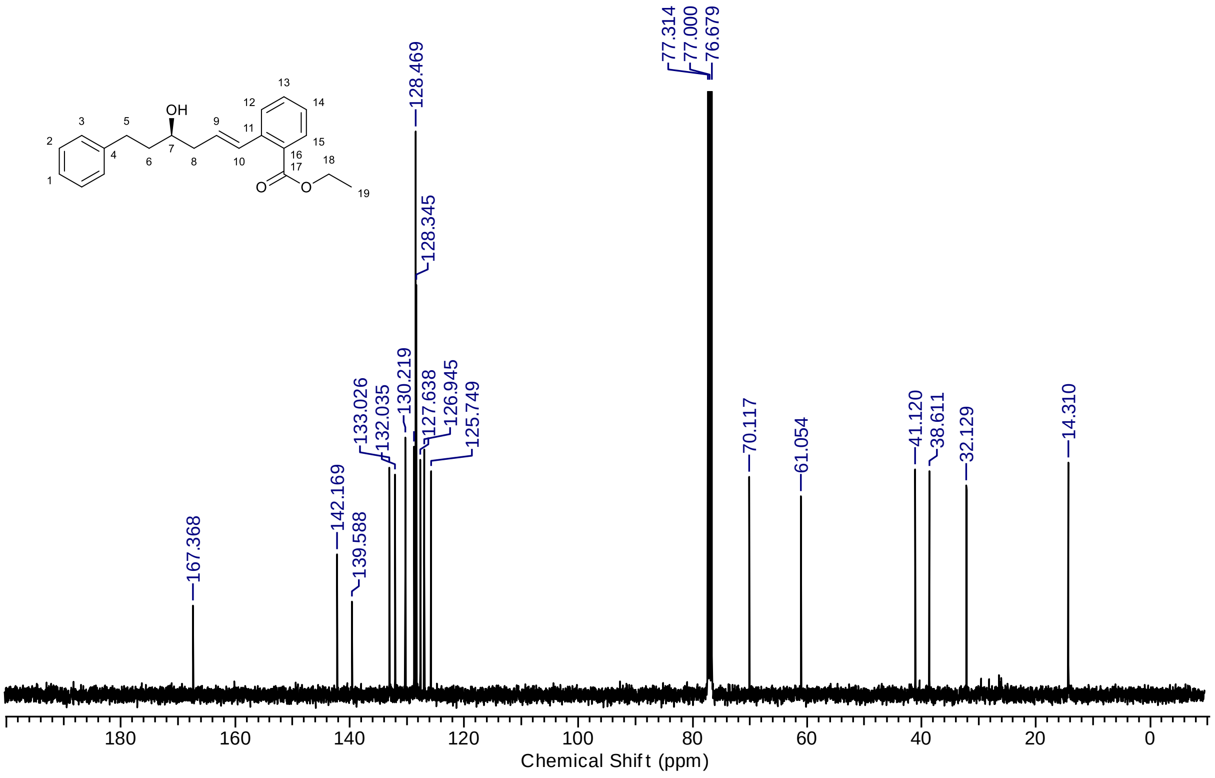

(*R*,*E*)-4-(4-Hydroxy-6-phenylhex-1-en-1-yl)benzonitrile (8f)

^{1}H-NMR (400 MHz, $CDCl_3$):

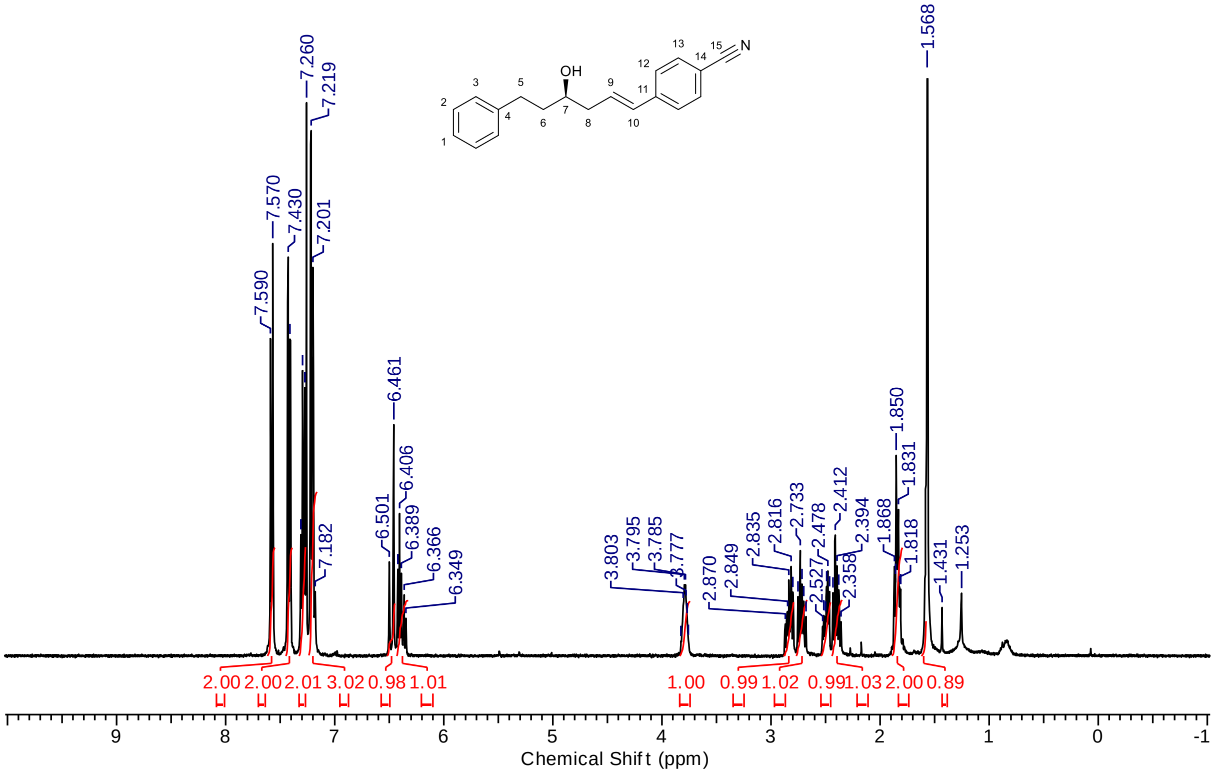

^{13}C-NMR (100 MHz, $CDCl_3$):

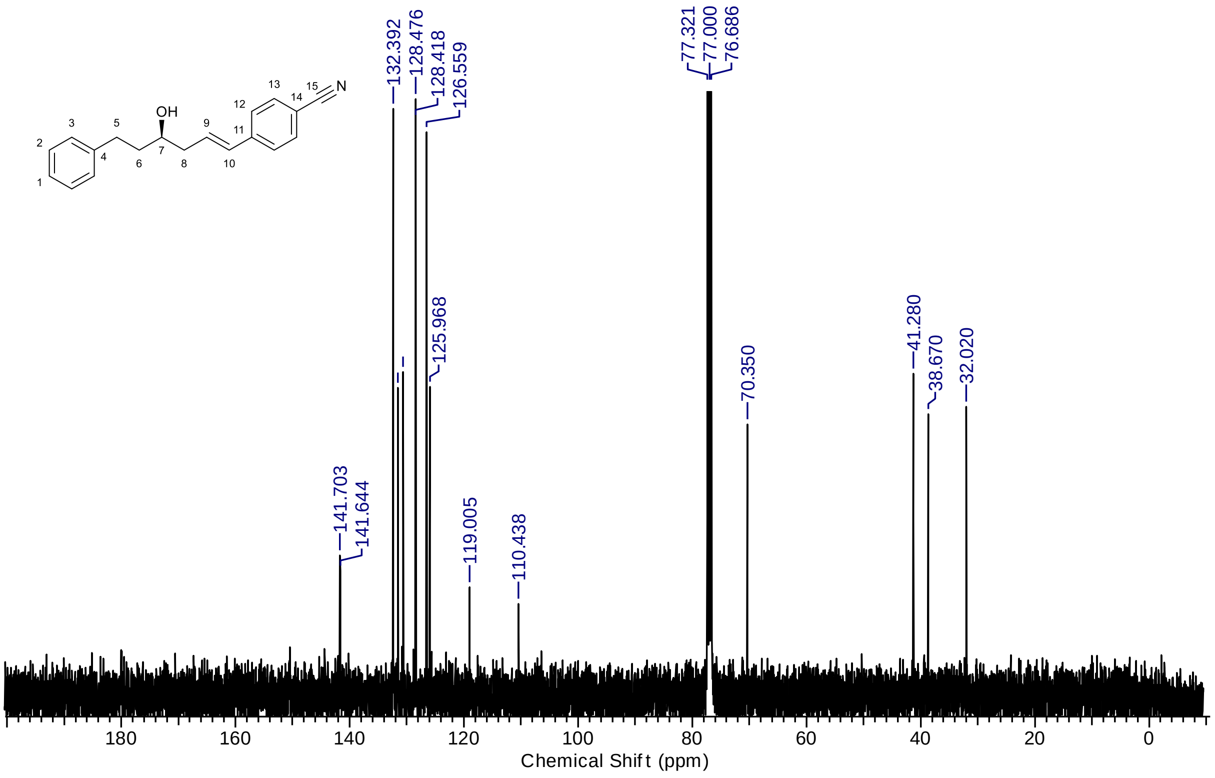

(*R*,5*E*,7*E*)-1,8-diphenylocta-5,7-dien-3-ol (8g)

^{1}H-NMR (400 MHz, $CDCl_3$):

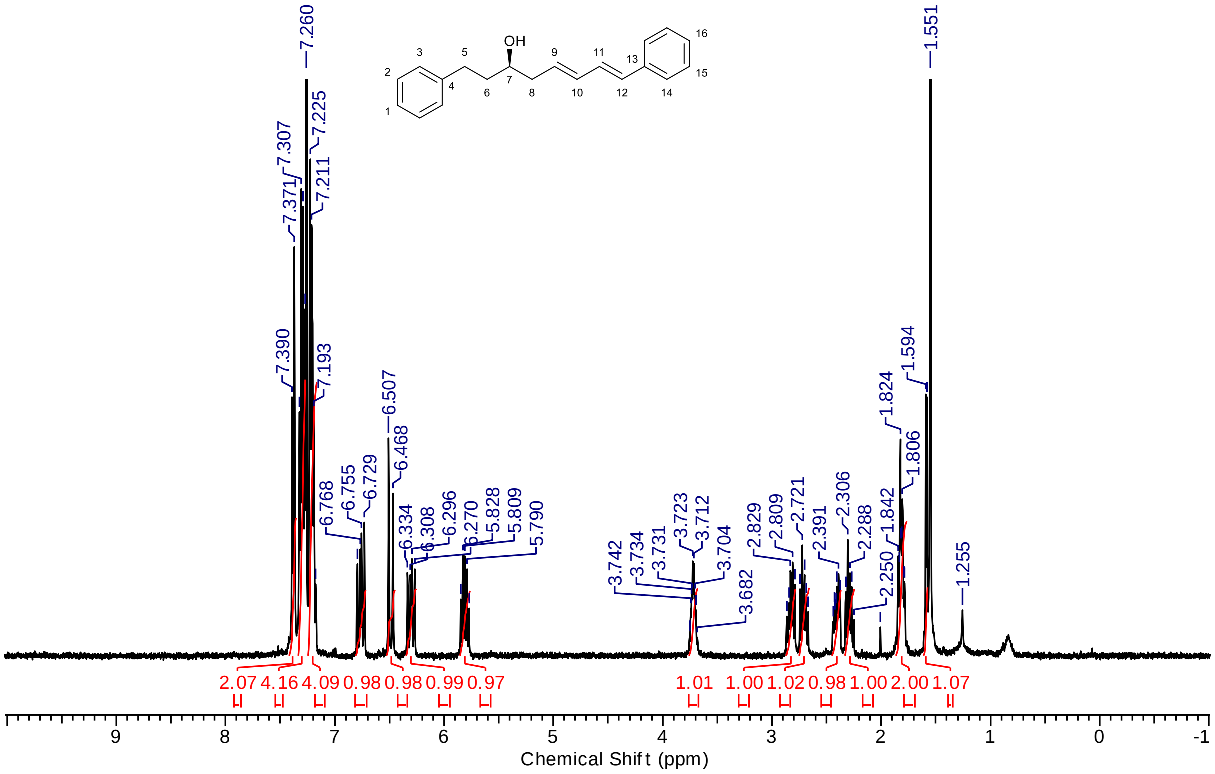

^{13}C-NMR (100 MHz, $CDCl_3$):

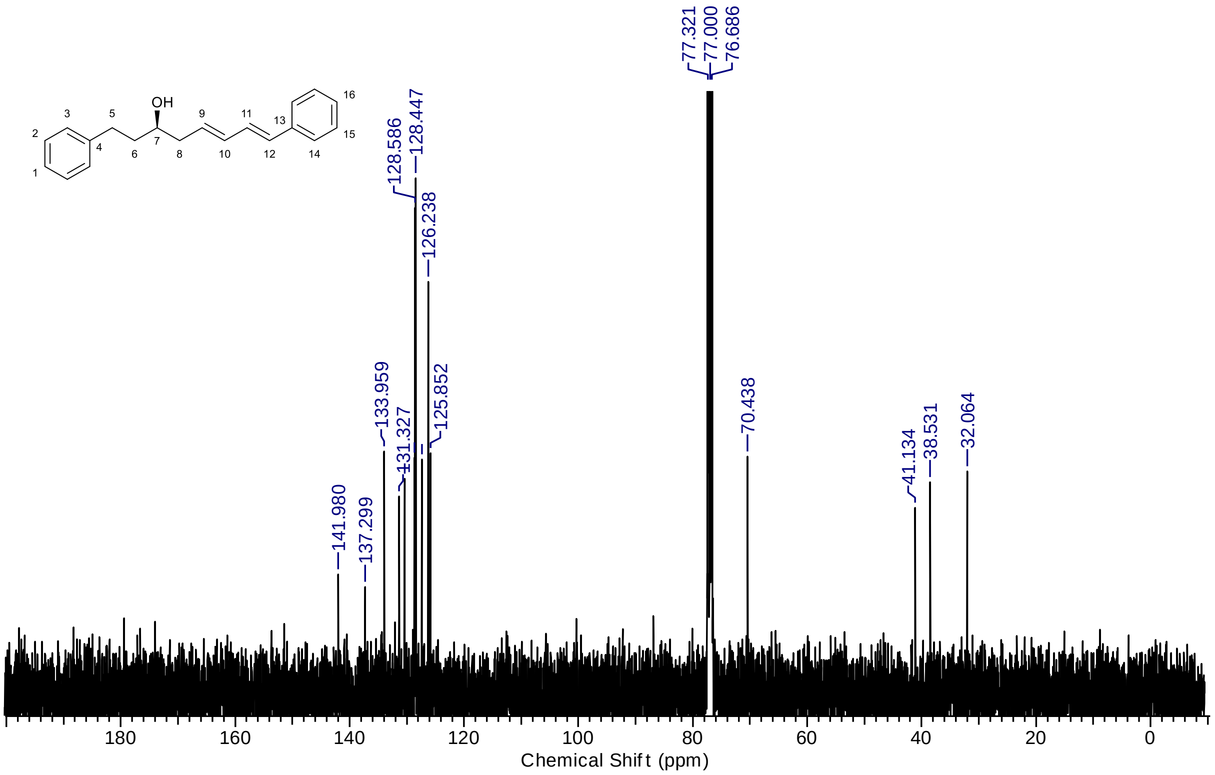

Ethyl (*R*,2*Z*,4*E*)-7-hydroxy-9-phenylnona-2,4-dienoate (8h)

^{1}H-NMR (400 MHz, $CDCl_3$):

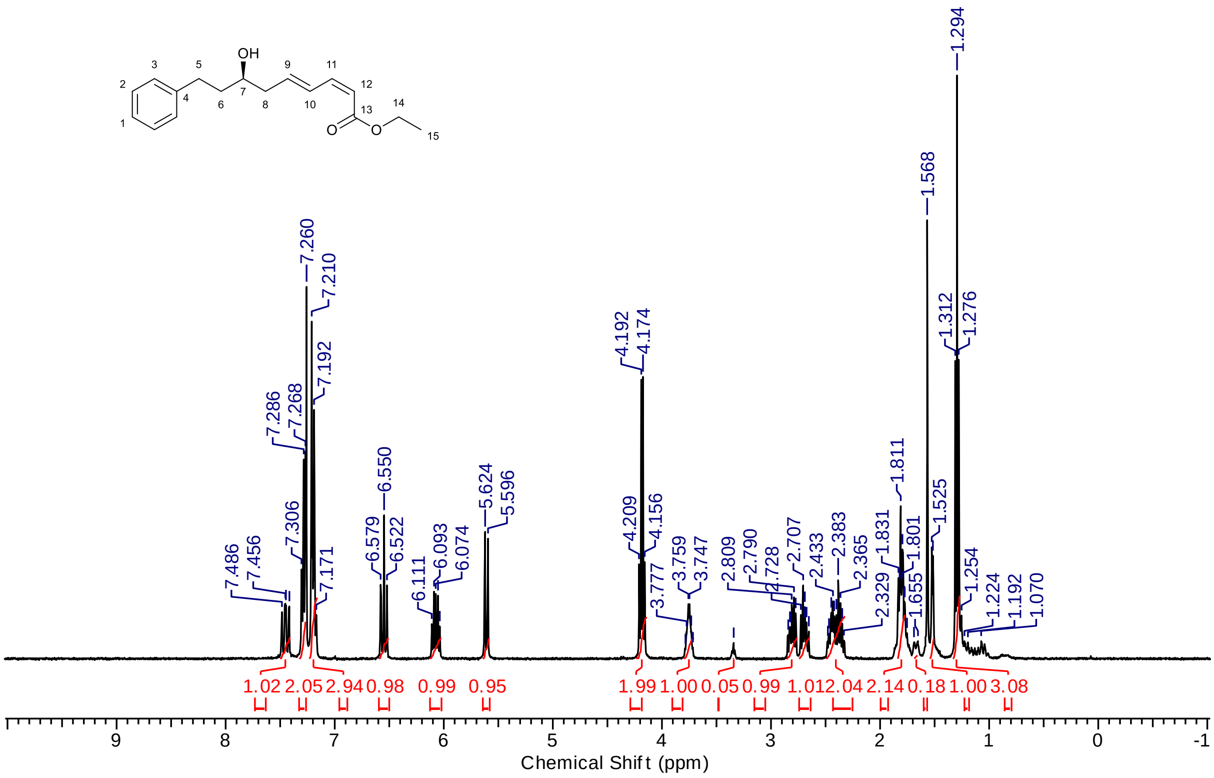

^{13}C-NMR (100 MHz, $CDCl_3$):

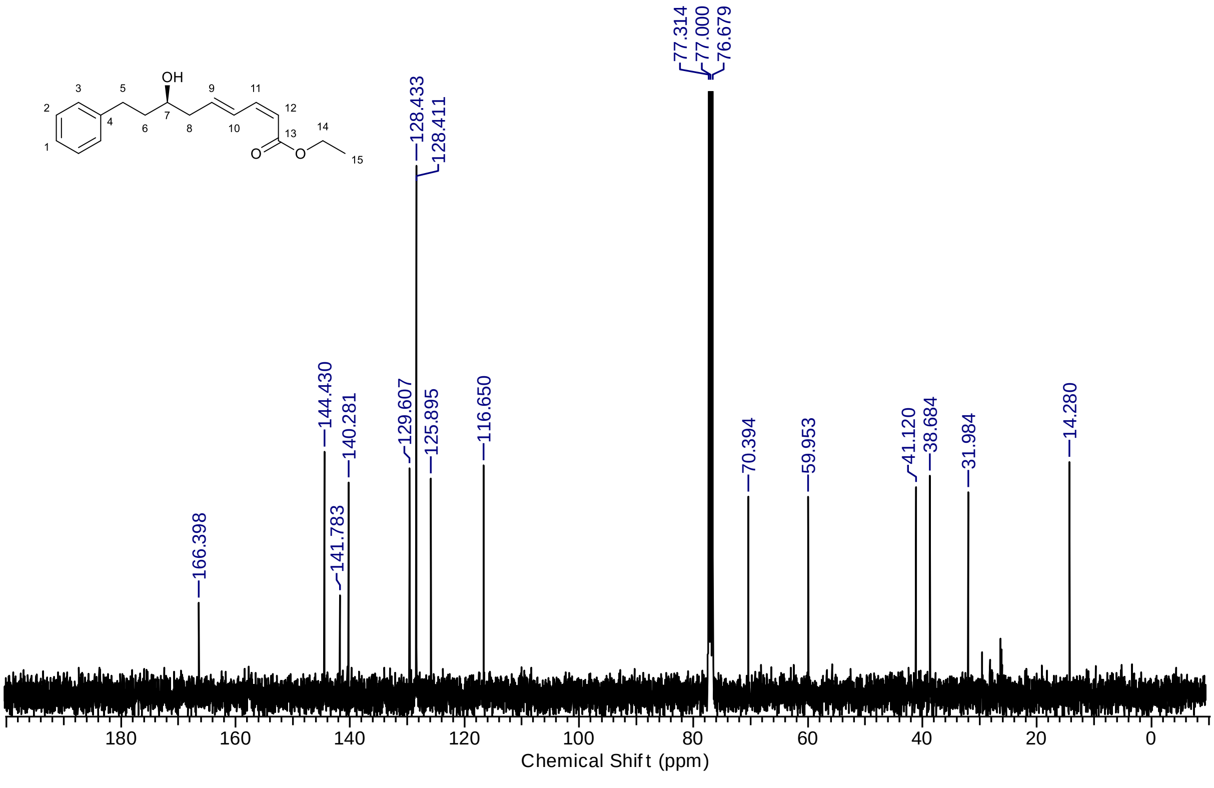

(4*R*,5*R*)-4,5-Dicyclohexyl-2-((R)-1,6-diphenylhex-5-yn-3-yl)-1,3,2-dioxaborolane (9a)

^{1}H-NMR (400 MHz, $CDCl_3$):

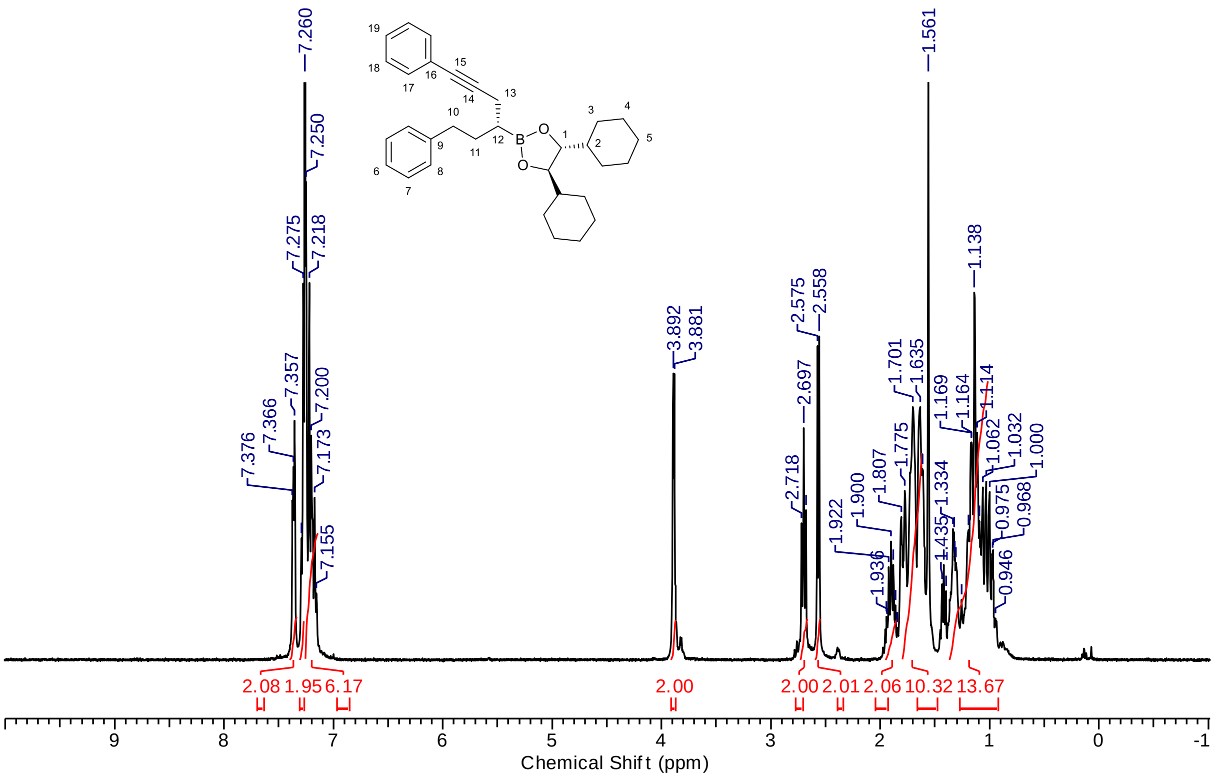

^{13}C-NMR (100 MHz, $CDCl_3$):

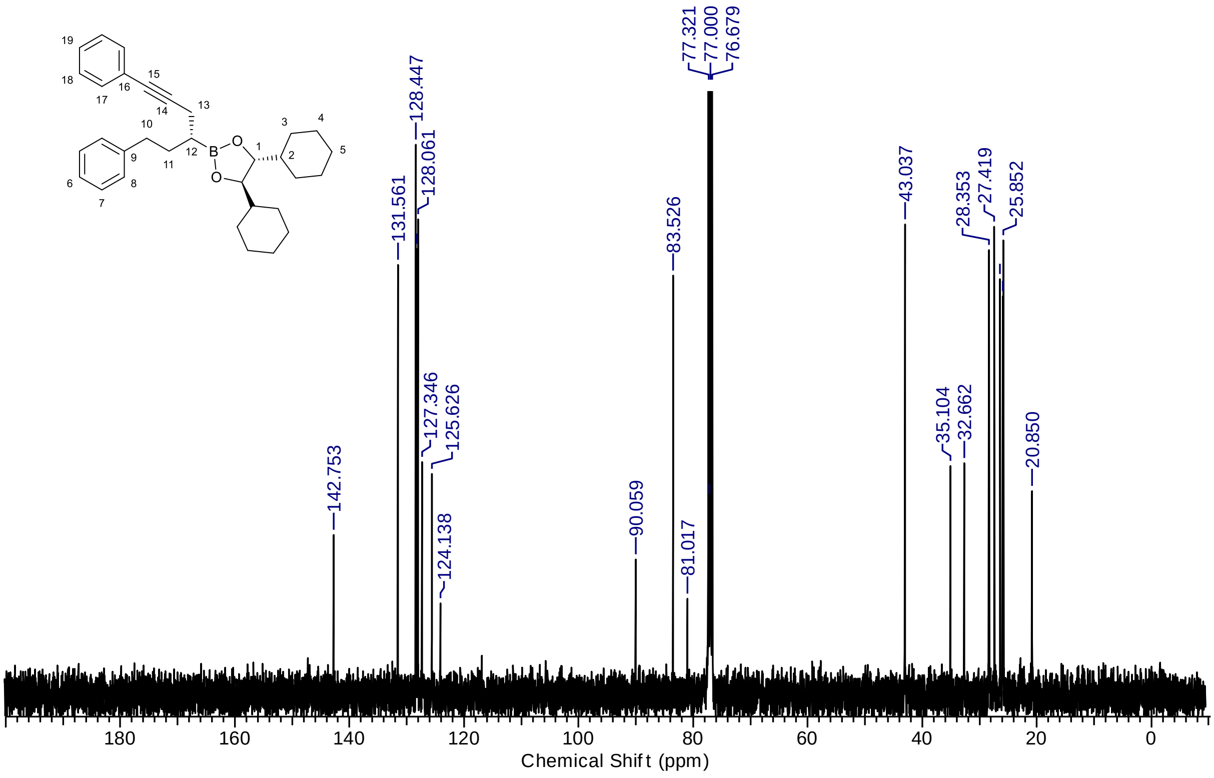

(4*R*,5*R*)-4,5-dicyclohexyl-2-((*R*)-1-phenyl-6-(*p*-tolyl)hex-5-yn-3-yl)-1,3,2-dioxaborolane (9b)

^{1}H-NMR (400 MHz, $CDCl_3$):

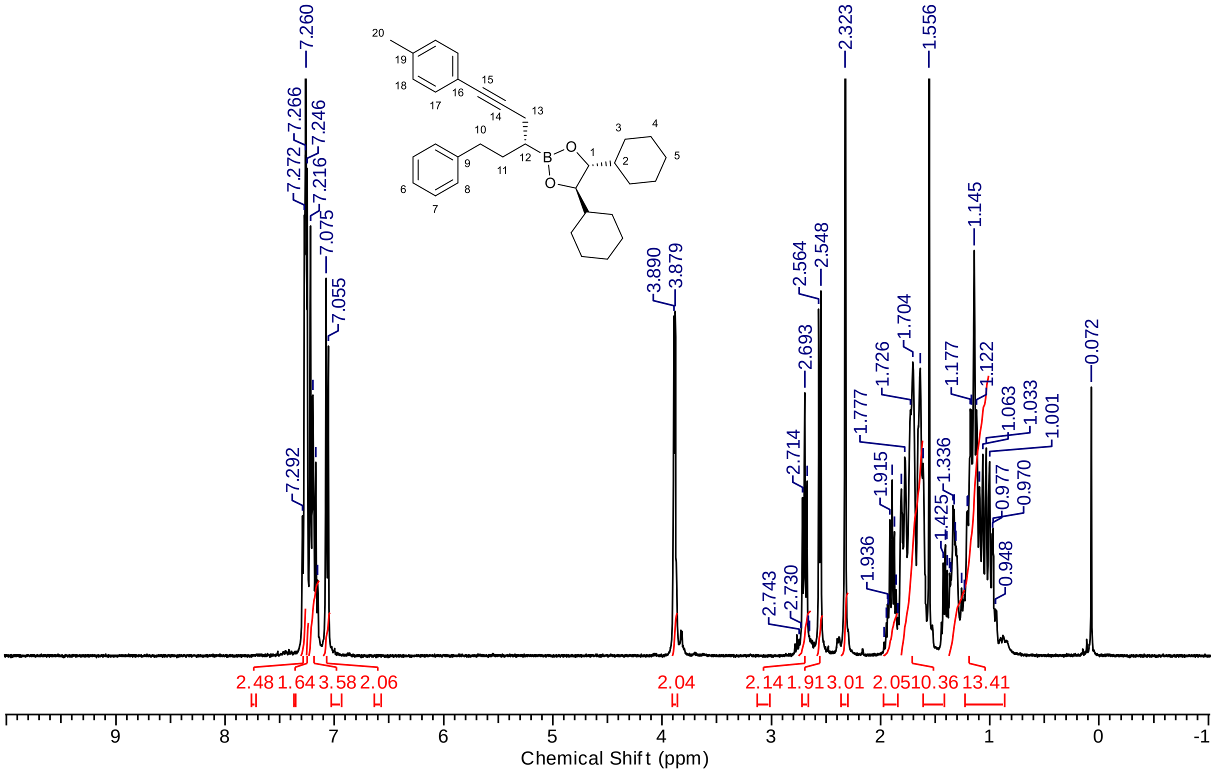

^{13}C-NMR (100 MHz, $CDCl_3$):

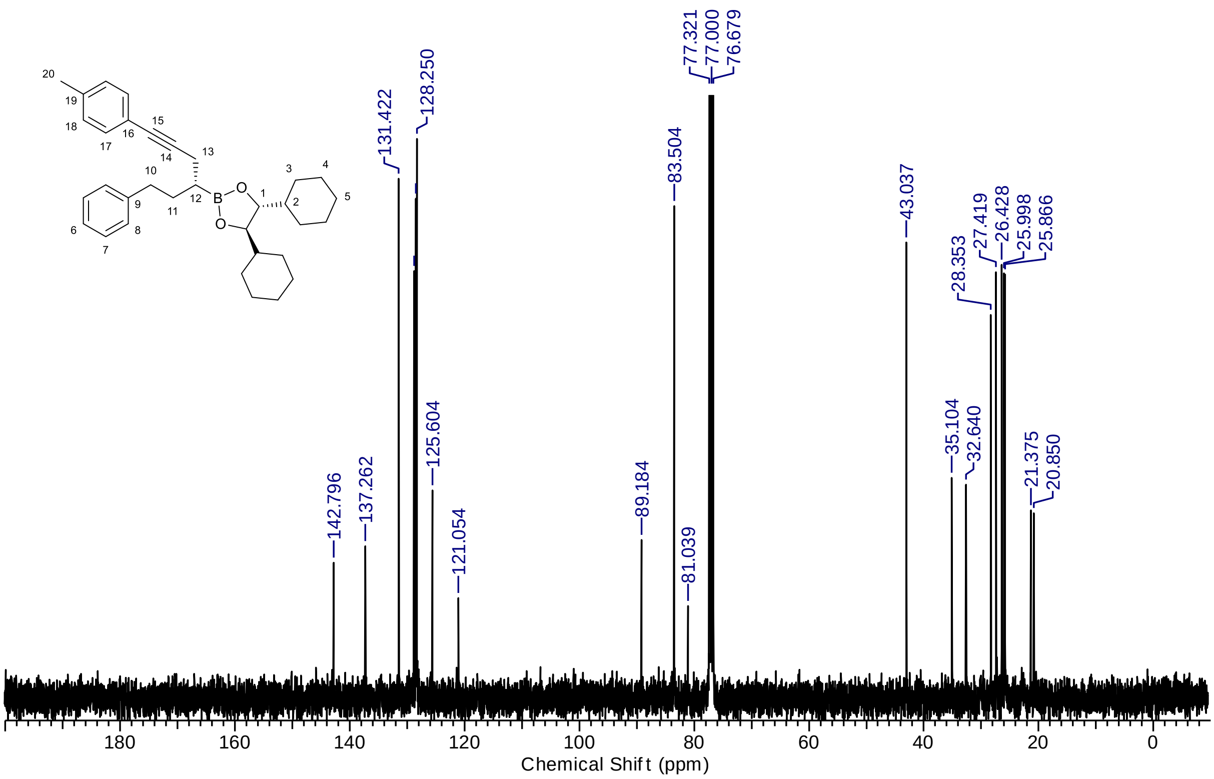

(4*R*,5*R*)-4,5-Dicyclohexyl-2-((*R*)-6-(4-methoxyphenyl)-1-phenylhex-5-yn-3-yl)-1,3,2-dioxaborolane (9c)

^{1}H-NMR (400 MHz, $CDCl_3$):

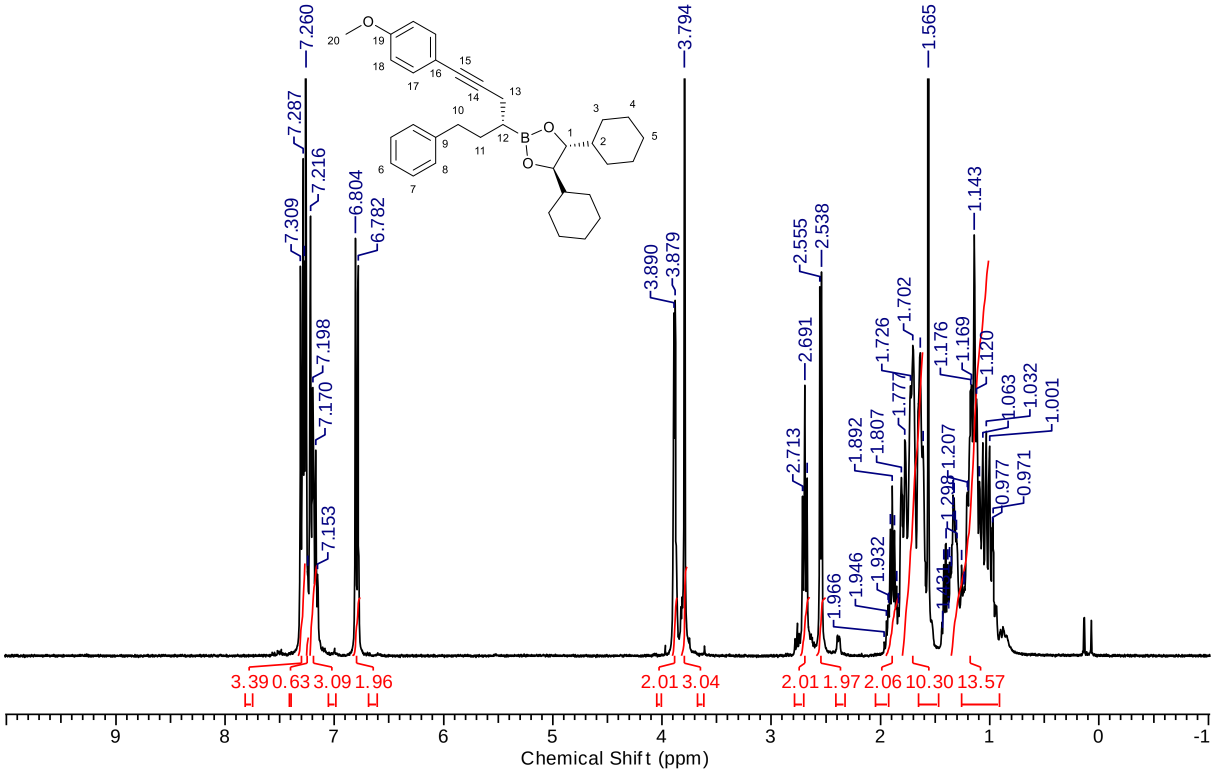

^{13}C-NMR (100 MHz, $CDCl_3$):

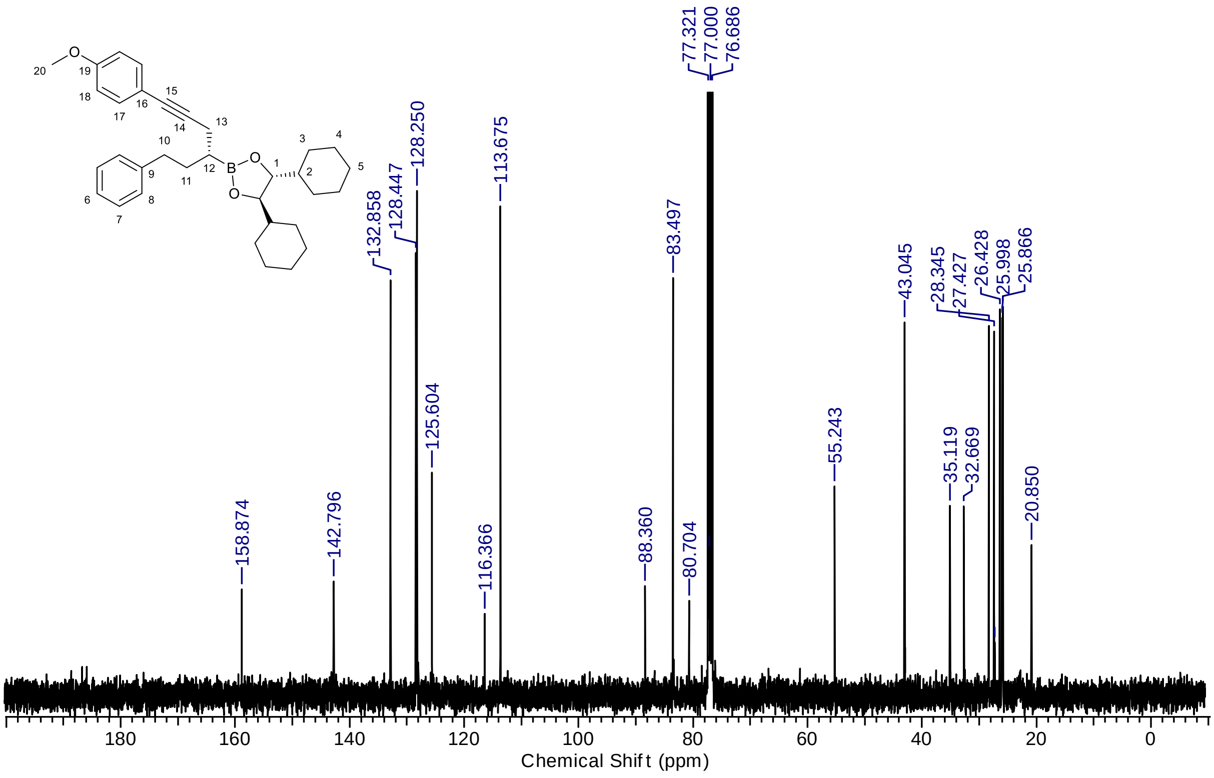

(4*R*,5*R*)-4,5-Dicyclohexyl-2-((*R*)-6-(4-fluorophenyl)-1-phenylhex-5-yn-3-yl)-1,3,2-dioxaborolane (9d)

^{1}H-NMR (400 MHz, $CDCl_3$):

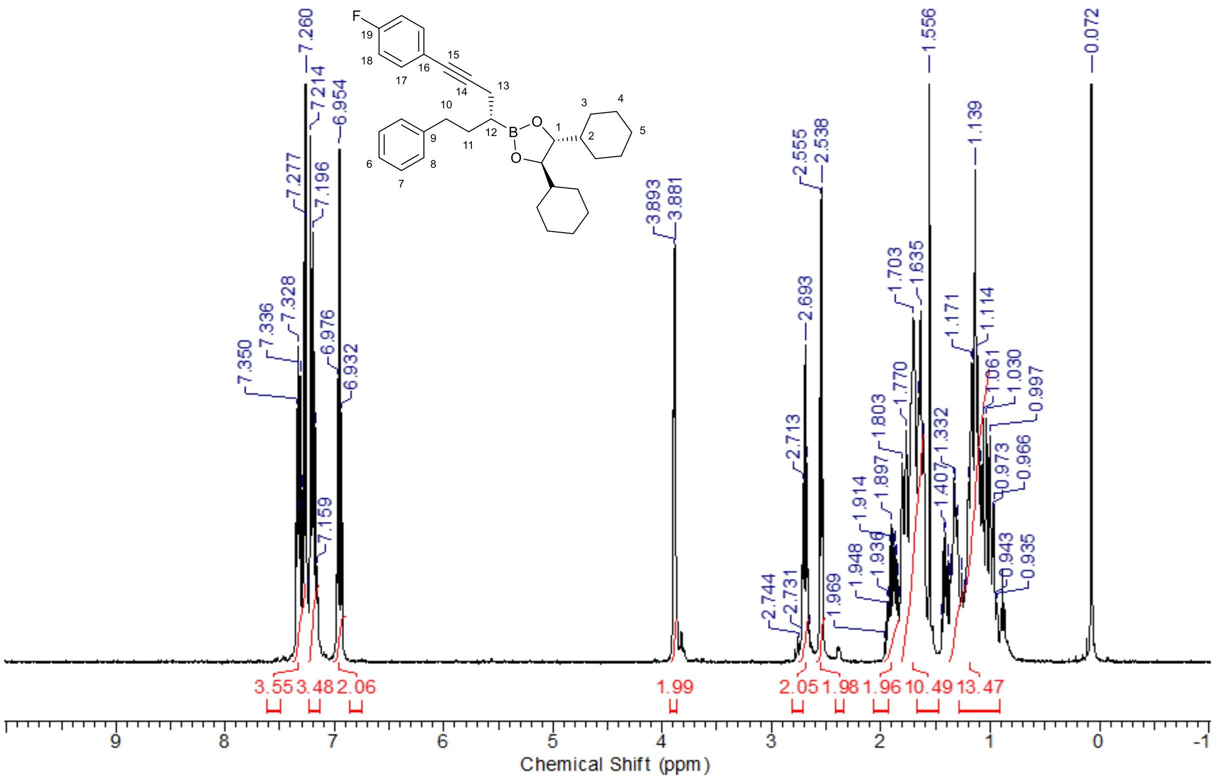

^{13}C-NMR (100 MHz, $CDCl_3$):

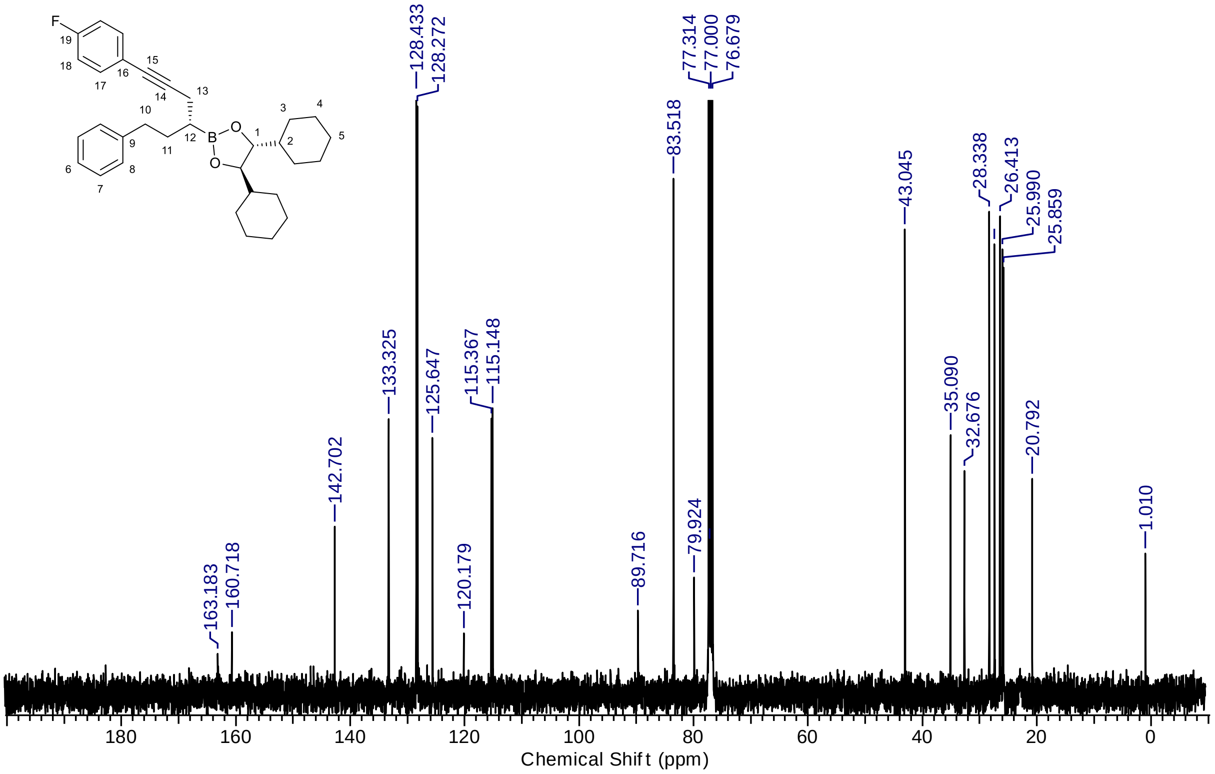

^{19}F-NMR (400 MHz, $CDCl_3$):

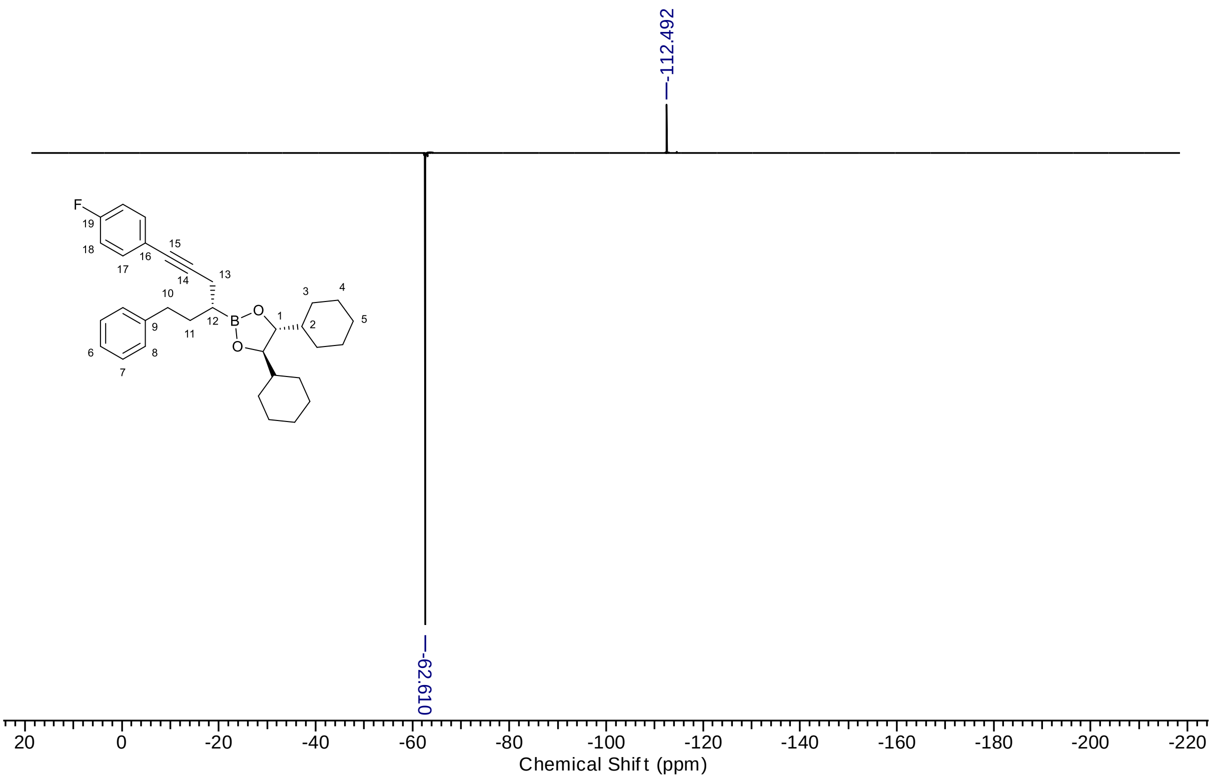

Reference substance: benzotrifluoride (−62.61 ppm)

(4*R*,5*R*)-4,5-dicyclohexyl-2-((*R*)-6-(2-methoxyphenyl)-1-phenylhex-5-yn-3-yl)-1,3,2-dioxaborolane (9e)

^{1}H-NMR (400 MHz, $CDCl_3$):

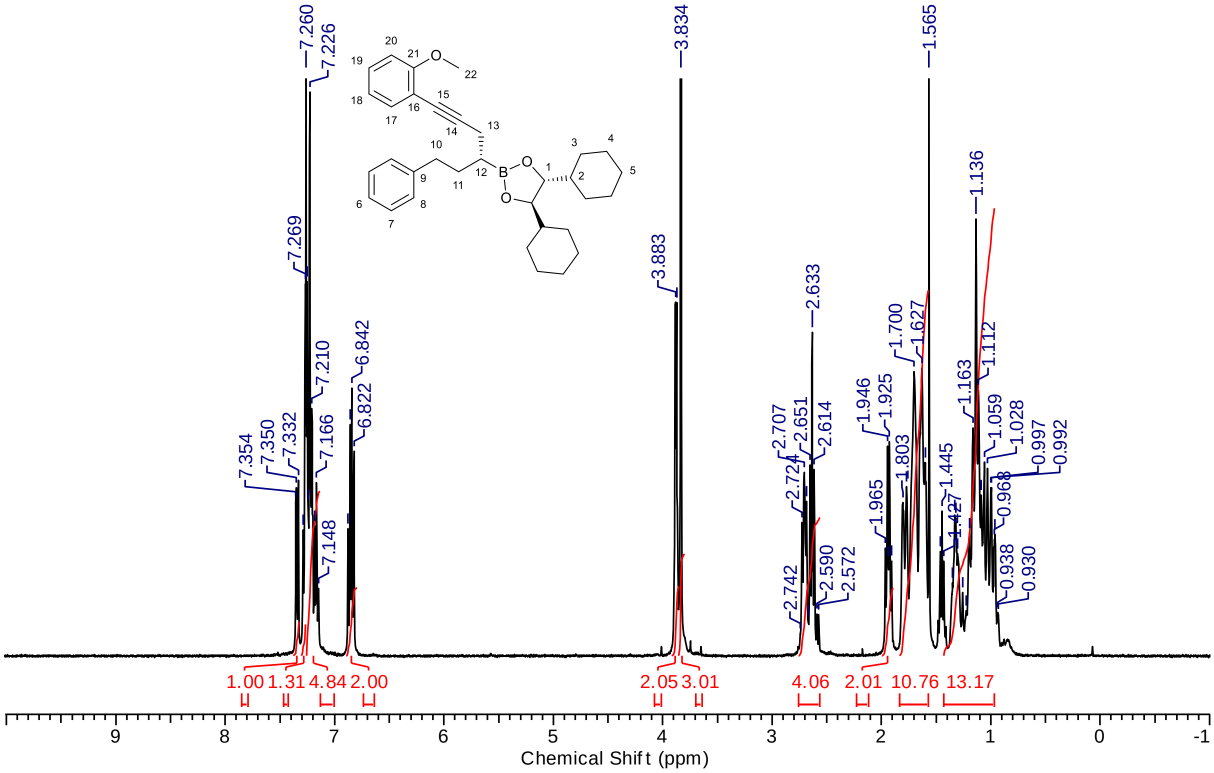

^{13}C-NMR (100 MHz, $CDCl_3$):

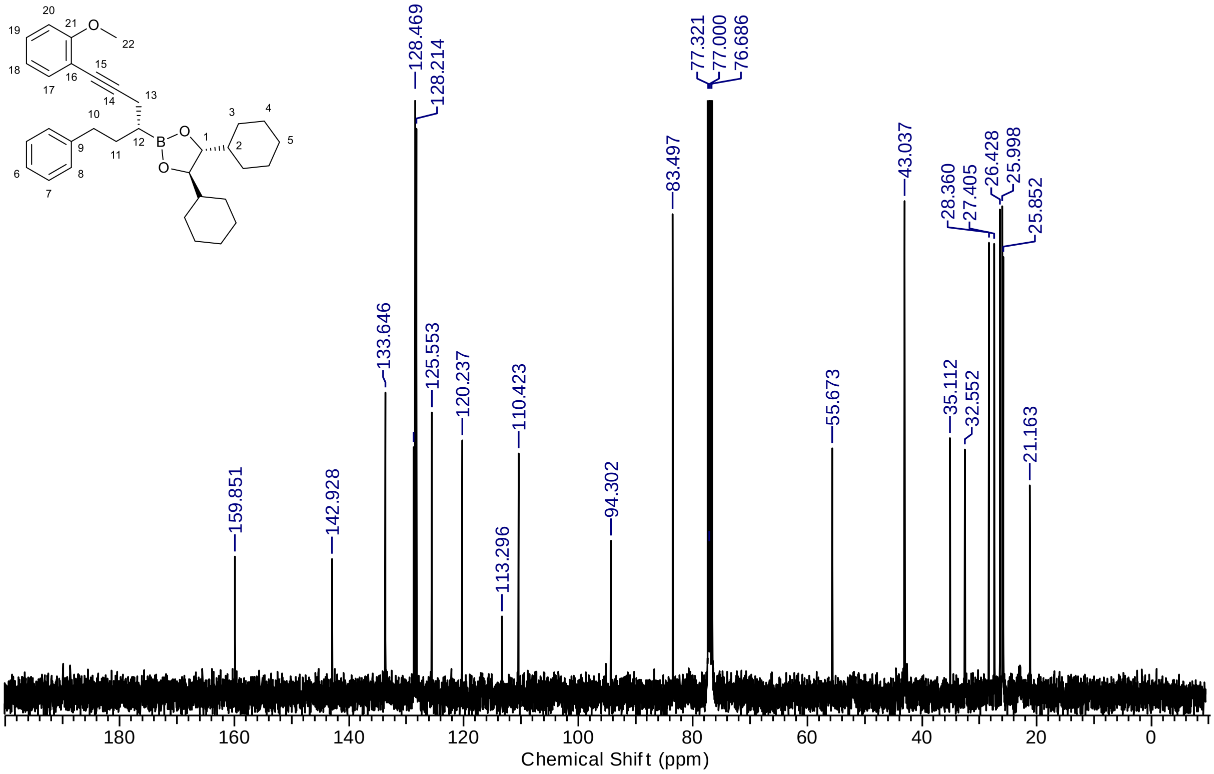

(4*R*,5*R*)-4,5-Dicyclohexyl-2-((*R*)-6-(4-nitrophenyl)-1-phenylhex-5-yn-3-yl)-1,3,2-dioxaborolane (9f)

^{1}H-NMR (400 MHz, $CDCl_3$):

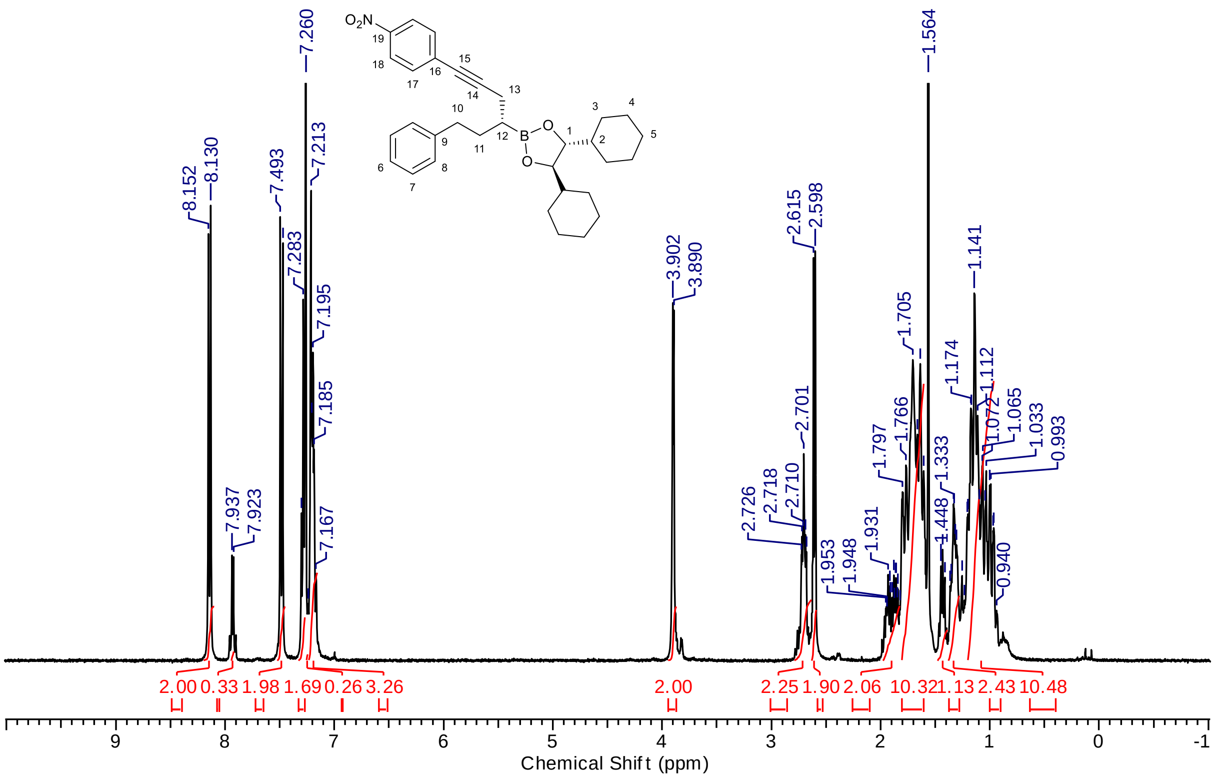

Additional signal arising from 1-iodo-4-nitrobenzene

^{13}C-NMR (100 MHz, $CDCl_3$):

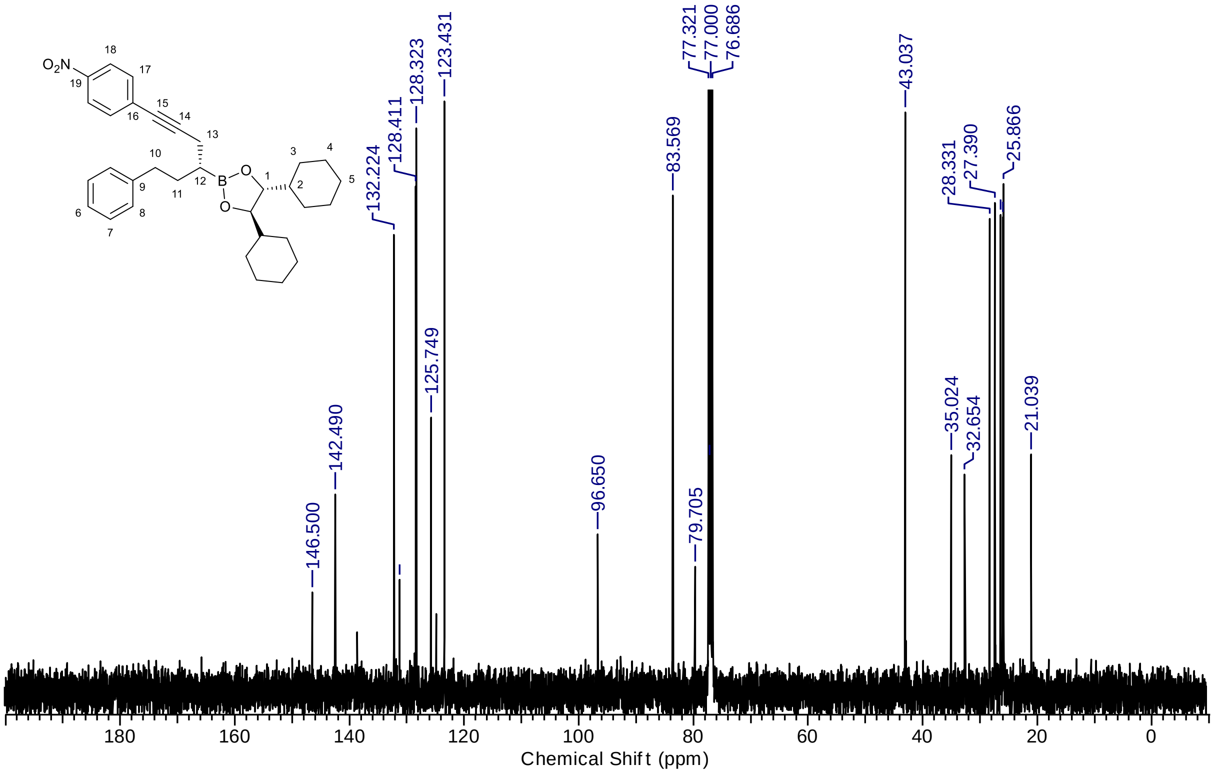

Additional signals arising from 1-iodo-4-nitrobenzene

(4*R*,5*R*)-4,5-Dicyclohexyl-2-((*R*,*Z*)-6-(2-methoxyphenyl)-1-phenylhex-5-en-3-yl)-1,3,2-dioxaborolane (10e)

^{1}H-NMR (400 MHz, $CDCl_3$):

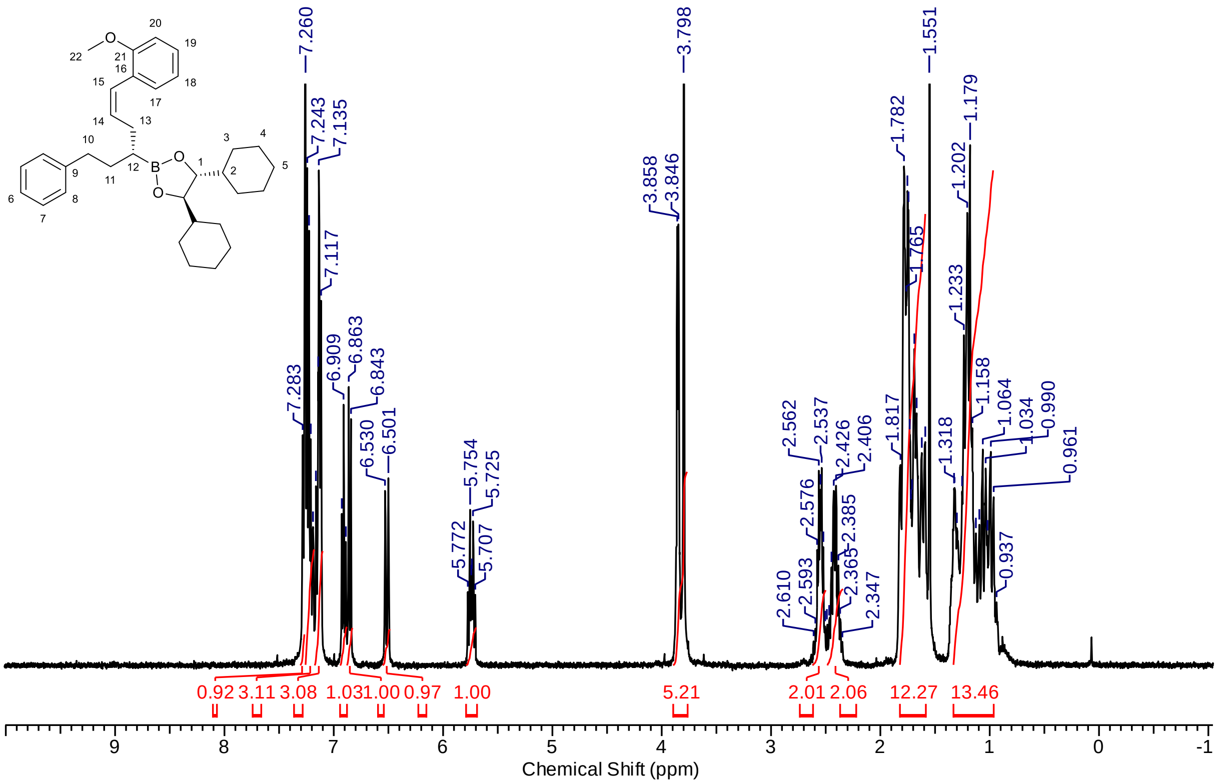

^{13}C-NMR (100 MHz, $CDCl_3$):

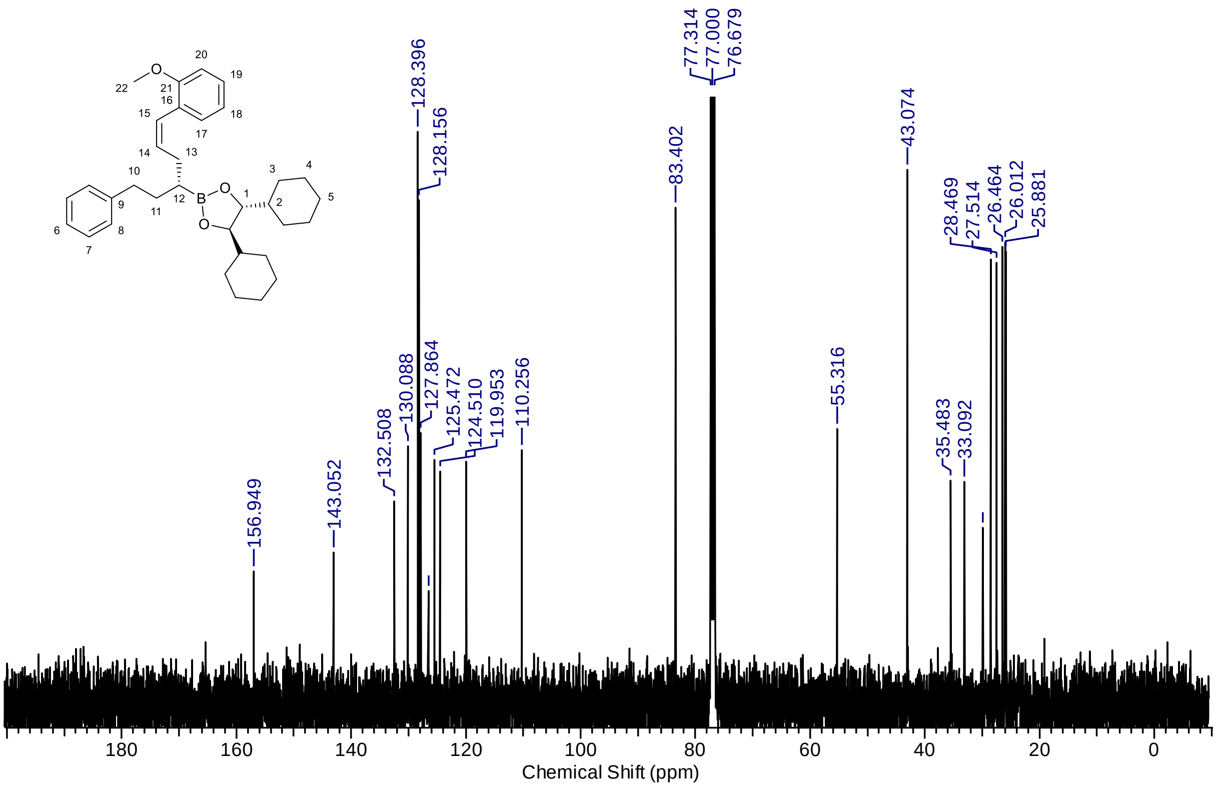

(*R*,*Z*)-1,6-diphenylhex-5-en-3-ol (11a)

^{1}H-NMR (400 MHz, $CDCl_3$):

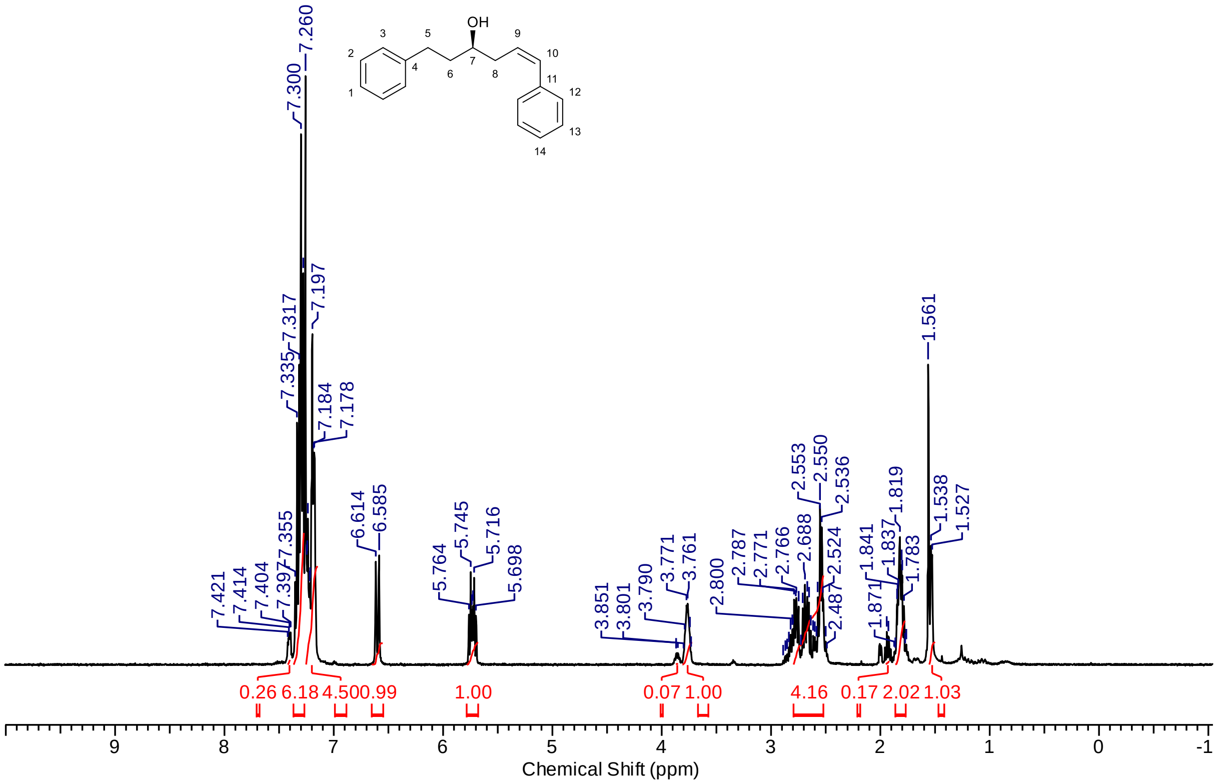

^{13}C-NMR (100 MHz, $CDCl_3$):

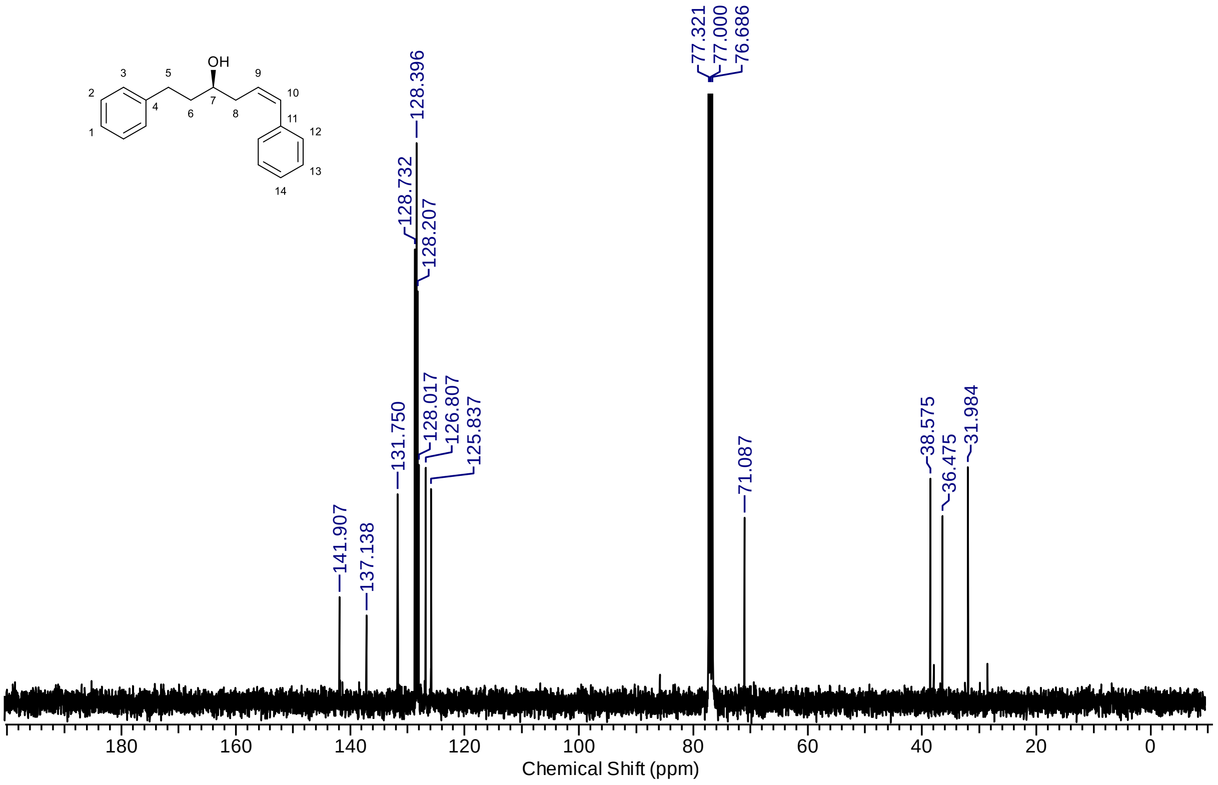

(*R*,*Z*)-1-Phenyl-6-(*p*-tolyl)hex-5-en-3-ol (11b)

^{1}H-NMR (400 MHz, $CDCl_3$):

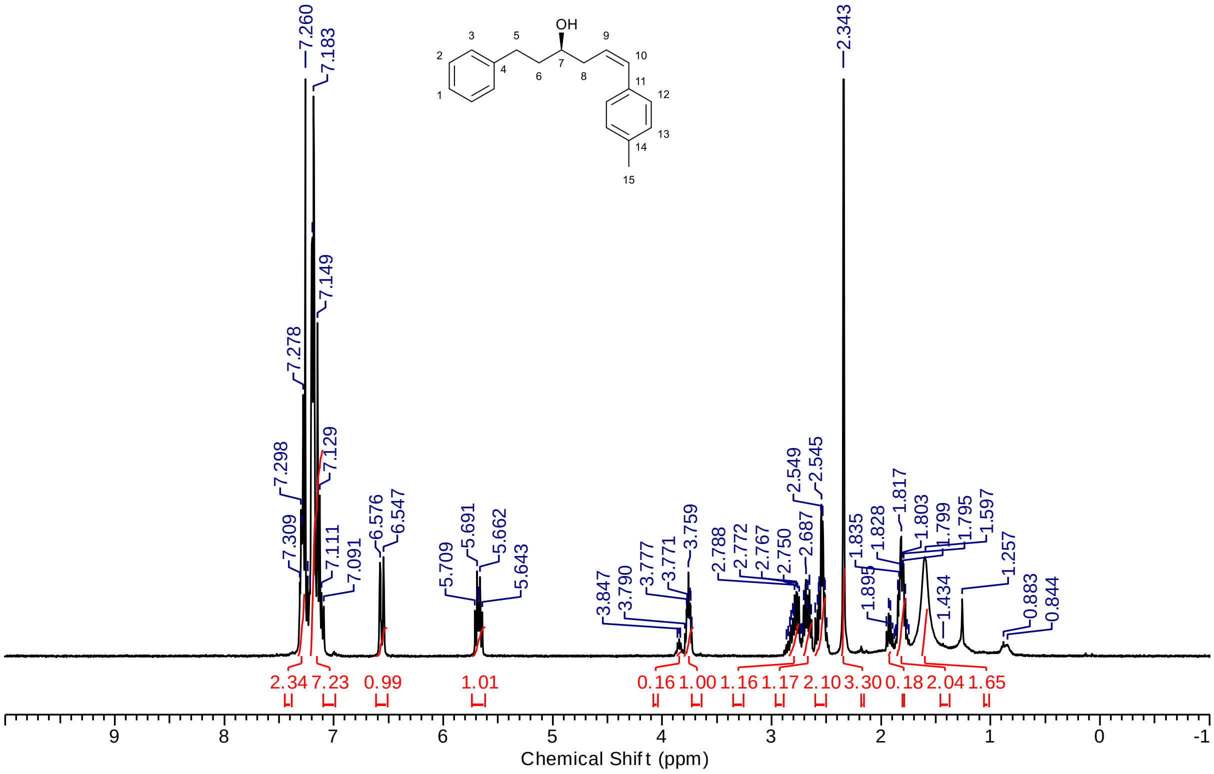

^{13}C-NMR (100 MHz, $CDCl_3$):

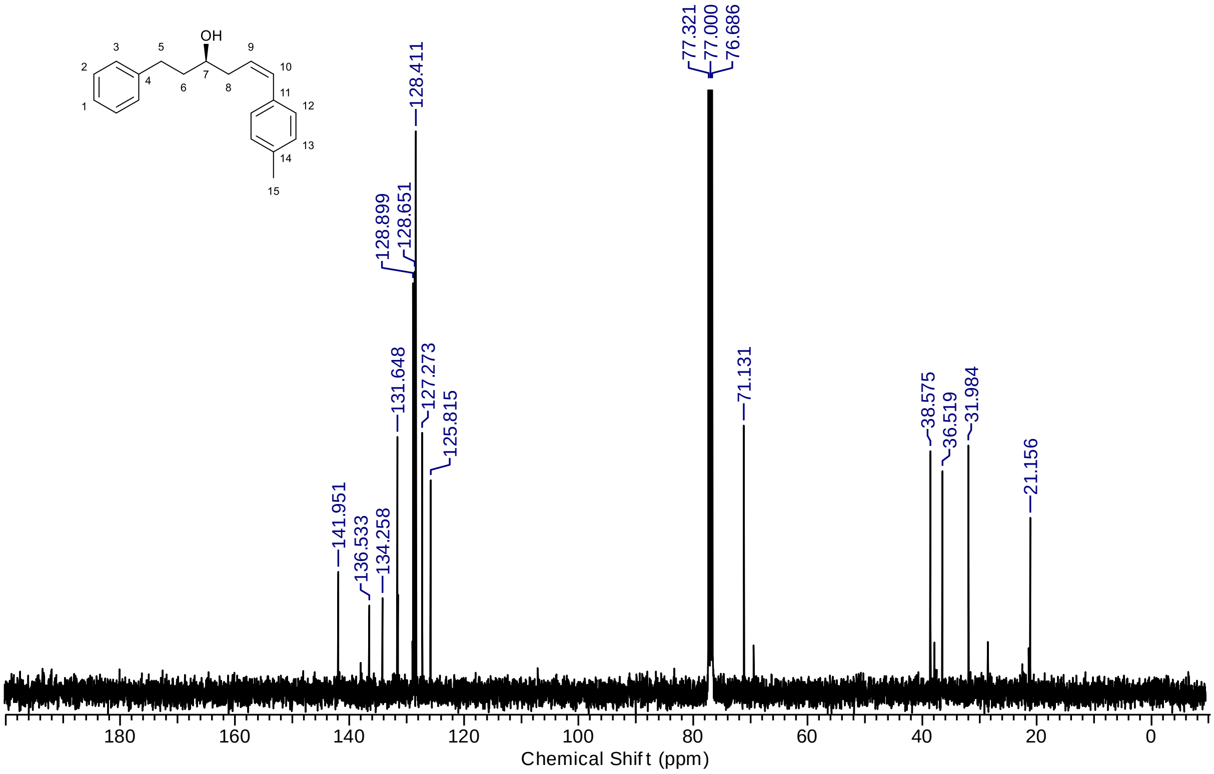

(*R*,*Z*)-6-(4-methoxyphenyl)-1-phenylhex-5-en-3-ol (11c)

^{1}H-NMR (400 MHz, $CDCl_3$):

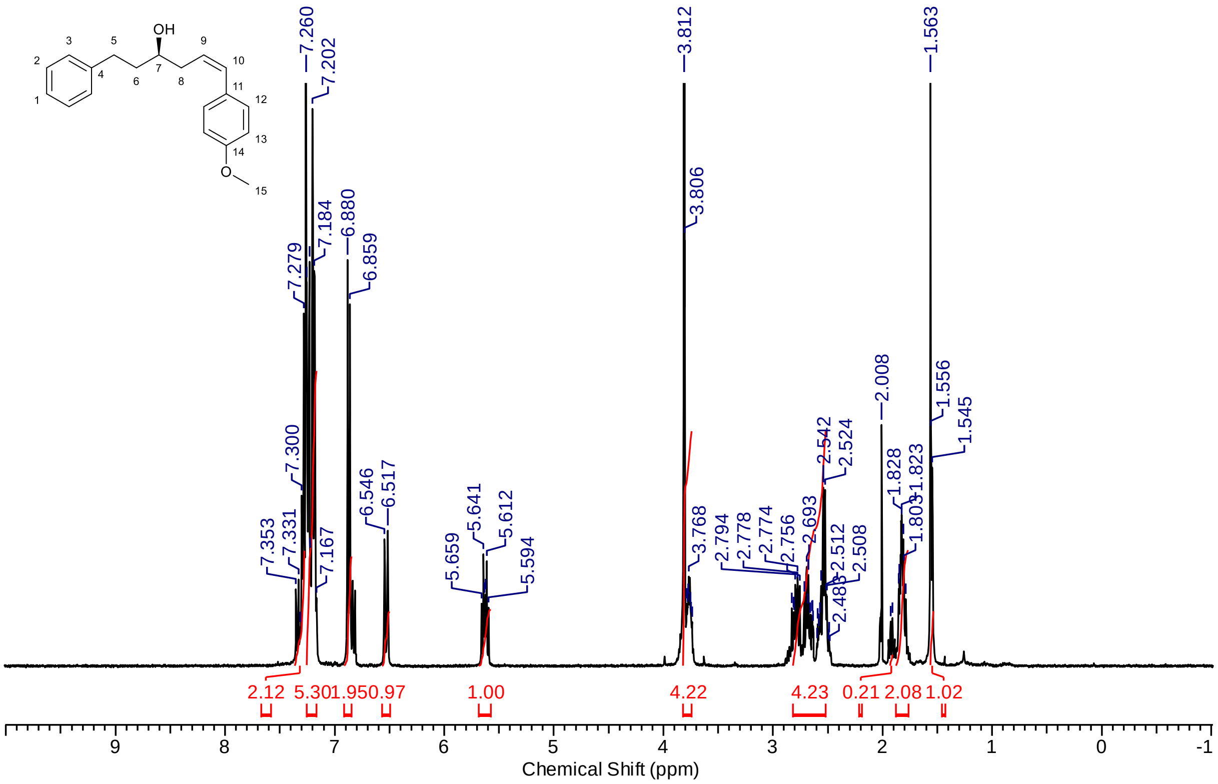

^{13}C-NMR (100 MHz, $CDCl_3$):

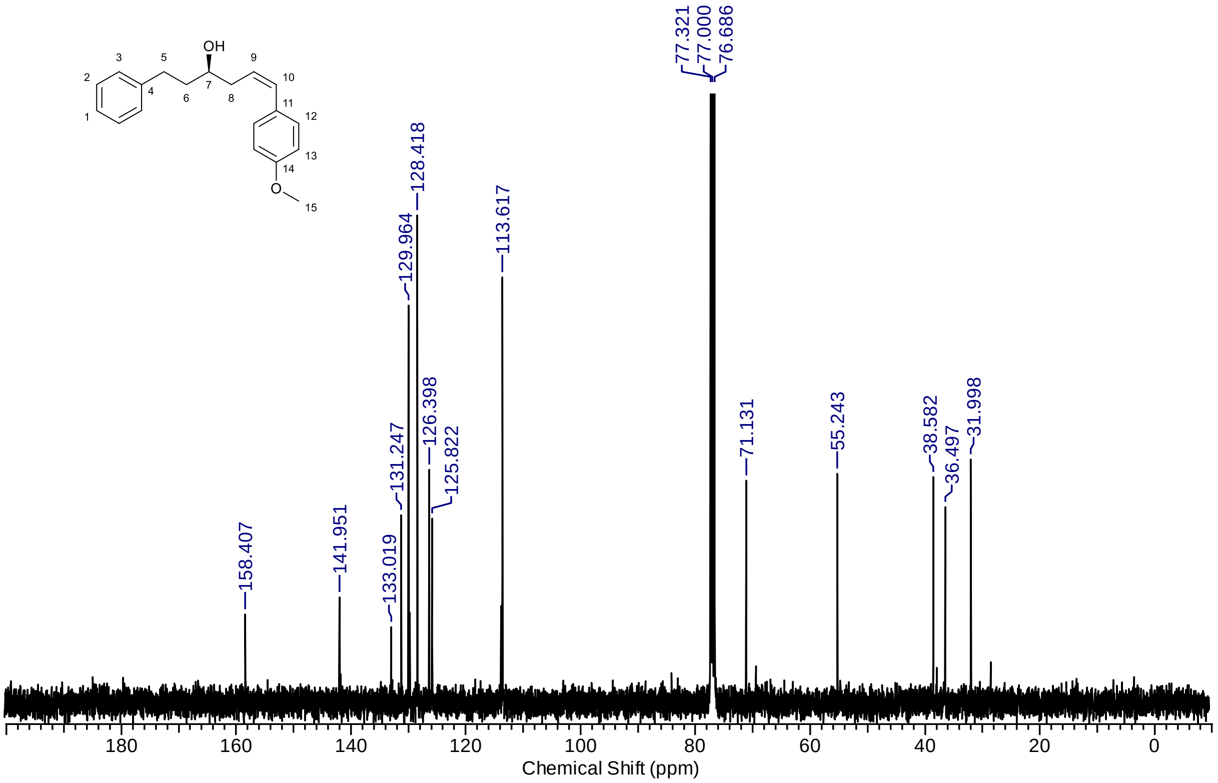

(*R*,*Z*)-6-(4-Fluorophenyl)-1-phenylhex-5-en-3-ol (11d)

^{1}H-NMR (400 MHz, $CDCl_3$):

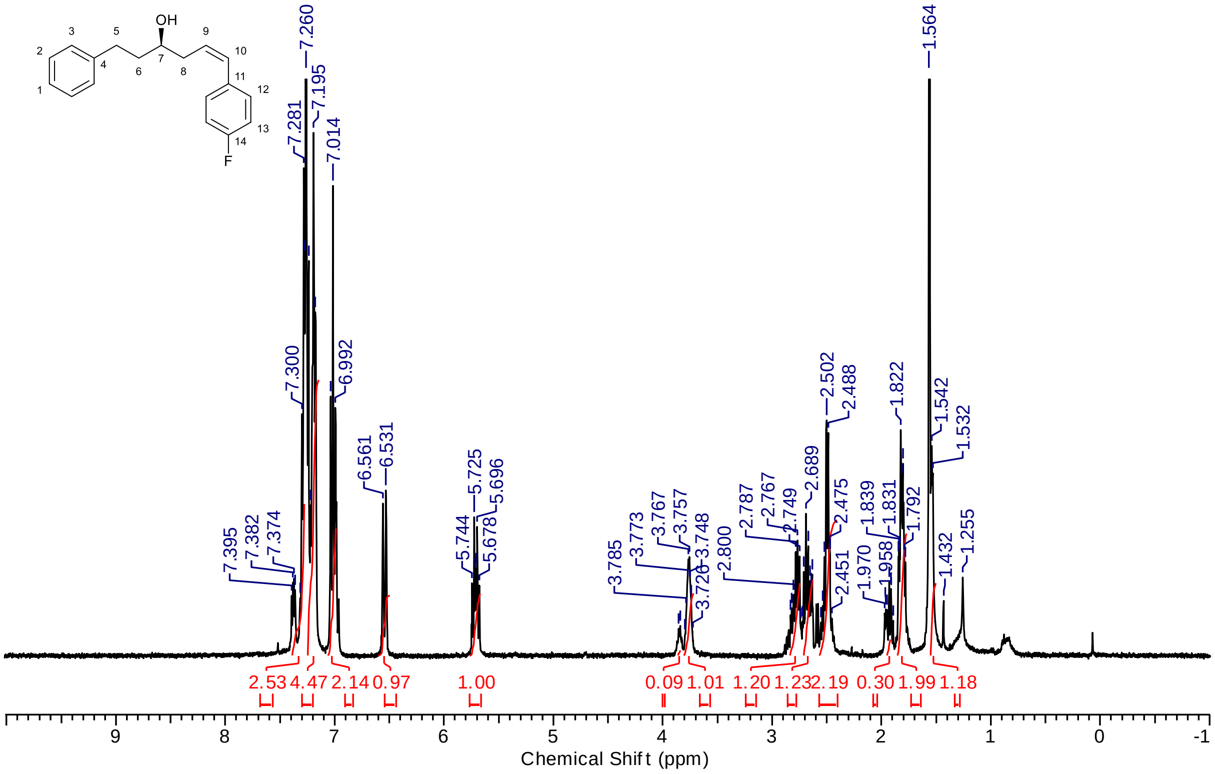

^{13}C-NMR (100 MHz, $CDCl_3$):

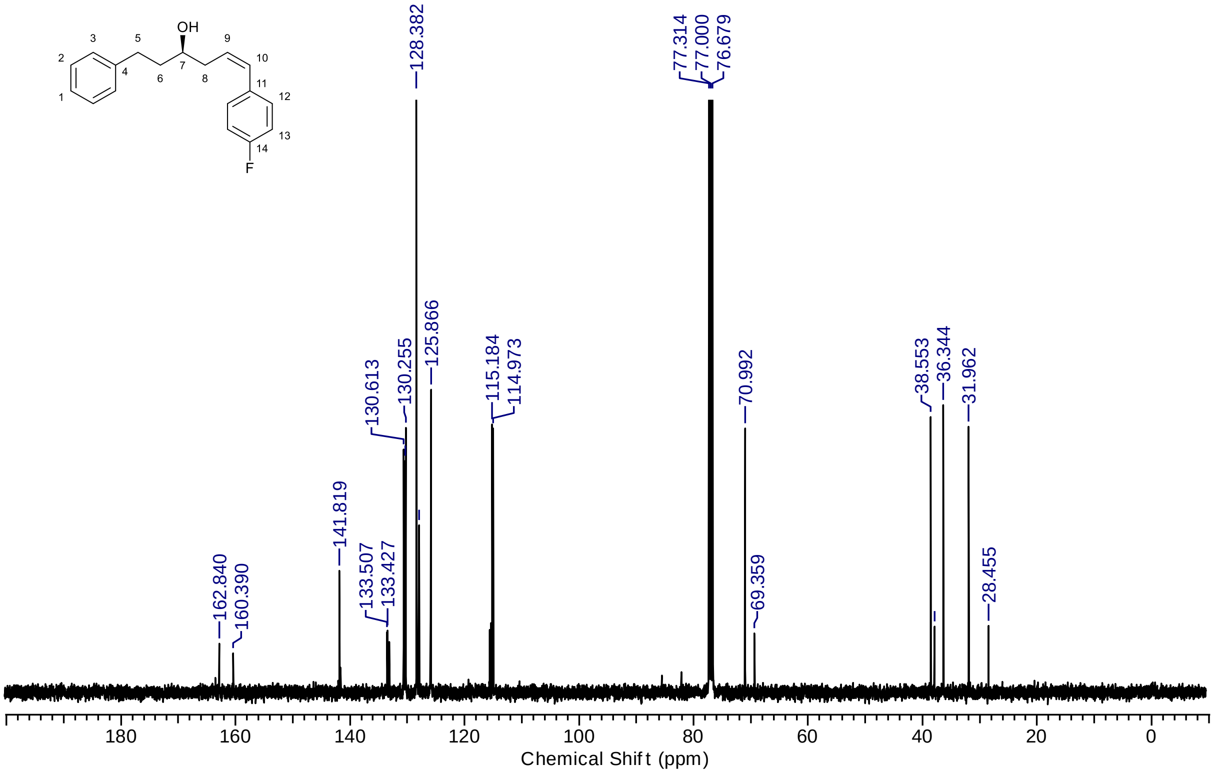

^{19}F-NMR (400 MHz, $CDCl_3$):

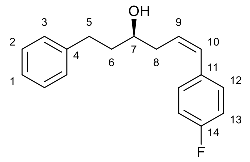

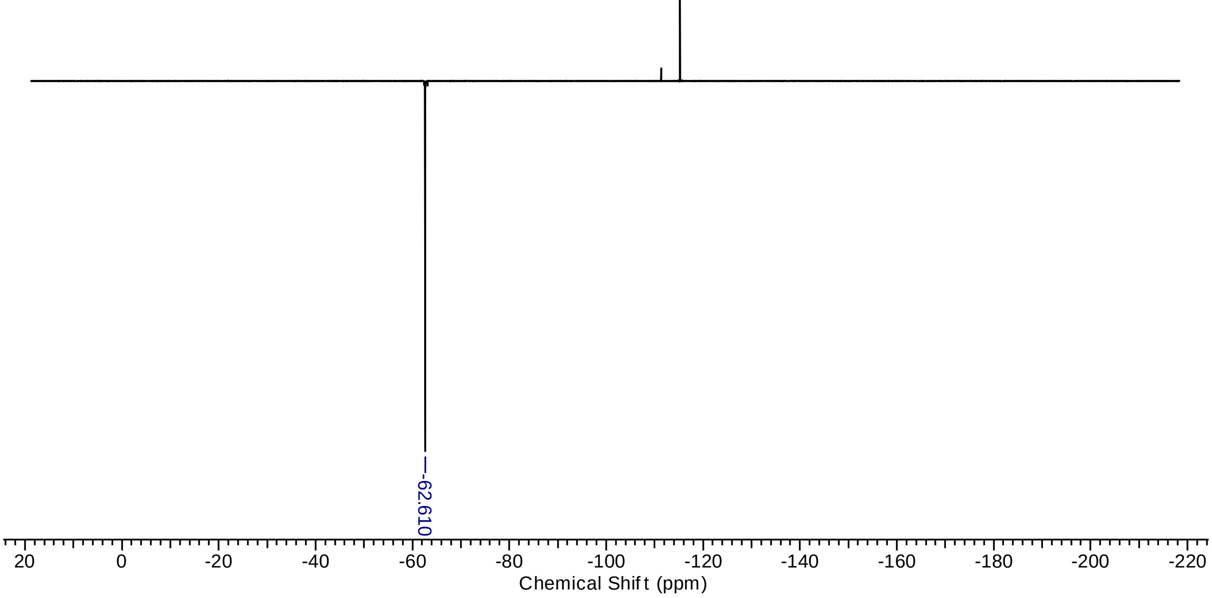

(4*R*,5*R*)-4,5-Dicyclohexyl-2-((*R*,*E*)-5-iodo-1-phenyl-6-(*p*-tolyl)hex-5-en-3-yl)-1,3,2-dioxaborolane (12b) and (4*R*,5*R*)-4,5-dicyclohexyl-2-((*R*,*E*)-6-iodo-1-phenyl-6-(*p*-tolyl)hex-5-en-3-yl)-1,3,2-dioxaborolane (13b)

^{1}H-NMR (400 MHz, $CDCl_3$):

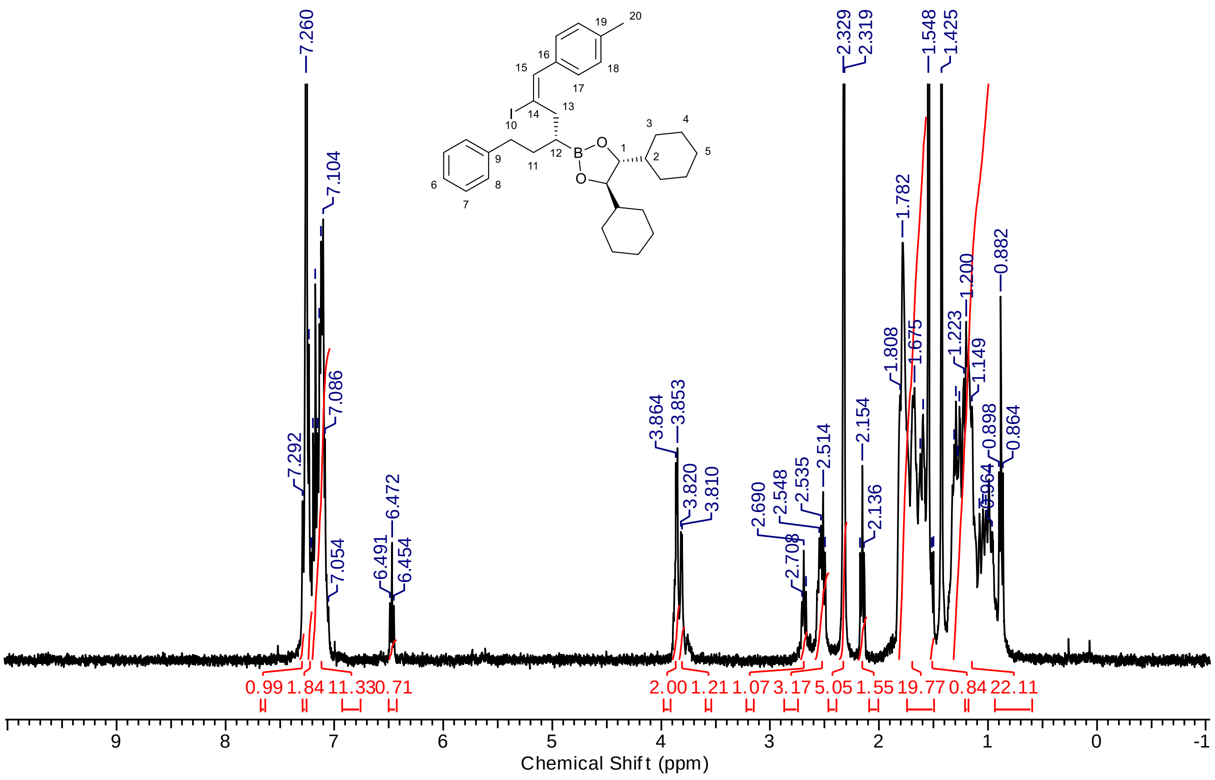

^{13}C-NMR (100 MHz, $CDCl_3$):

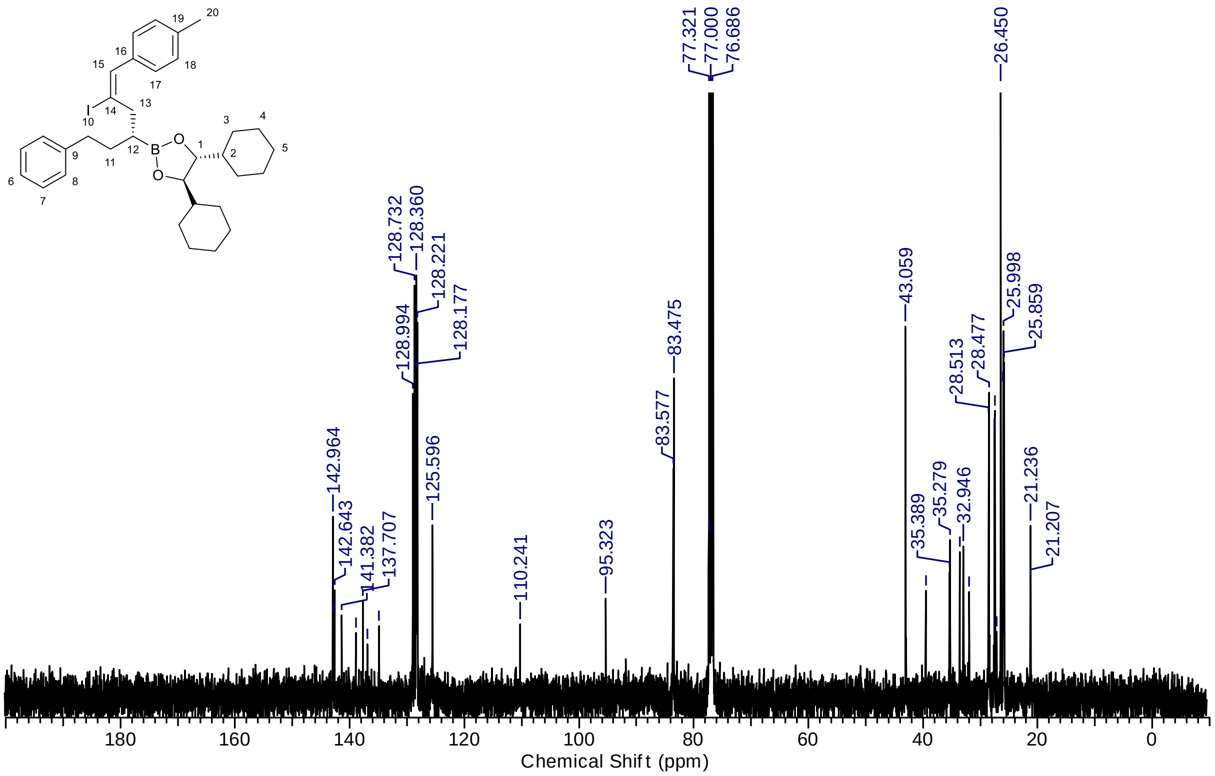

References

1 A. Krasovskiy and P. Knochel, *Synthesis* , 2006, 890–891.

2 S. Nave, R. P. Sonawane, T. G. Elford and V. K. Aggarwal, *J. Am. Chem. Soc.*, 2010, **132**, 17096–17098.

3 T. G. Elford, S. Nave, R. P. Sonawane and V. K. Aggarwal, *J. Am. Chem. Soc.*, 2011, **133**, 16798–16801.

4 C. Cai and A. Vasella, *Helv. Chim. Acta*, 1995, **78**, 732–757.

5 V. Navickas, C. Rink and M. E. Maier, *Synlett*, 2011, 191–194.

6 K. Nakatani, A. Okamoto, T. Matsuno and I. Saito, *J. Am. Chem. Soc.*, 1998, **120**, 11219–11225.

7 E. M. Carreira and J. Du Bois, *J. Am. Chem. Soc.*, 1995, **117**, 8106–8125.

8 G. A. Molander and S. R. Wisniewski, *J. Am. Chem. Soc.*, 2012, **134**, 16856.

9 C. Drescher and R. Brückner, *Org. Lett.*, 2021, **23**, 6194–6199.
